# Neuronal lipofuscinosis caused by Kufs disease/CLN4 DNAJC5 mutations but not by a CSPα/DNAJC5 deficiency

**DOI:** 10.1126/sciadv.ads3393

**Published:** 2025-05-21

**Authors:** Santiago López-Begines, Nozha Borjini, Ángela Lavado-Roldán, Cristina Mesa-Cruz, Fabiola Mavillard, Vera I. Wiersma, Fátima Rubio-Pastor, Emanuela Tumini, Carmen Paradela-Leal, María L. Chiclana-Valcárcel, Carolina Aguado, Rafael Luján, Wiep Scheper, José L. Nieto-González, Rafael Fernández-Chacón

**Affiliations:** ^1^ Instituto de Biomedicina de Sevilla (IBiS, Hospital Universitario Virgen del Rocío/CSIC/Universidad de Sevilla), Departamento de Fisiología Médica y Biofísica, Facultad de Medicina, and CIBERNED ISCIII, Seville, Spain.; ^2^ Synaptic Structure Laboratory, Instituto de Investigación en Discapacidades Neurológicas (IDINE), Departamento de Ciencias Médicas, Facultad de Medicina, Universidad Castilla-La Mancha, Albacete, Spain.; ^3^ Amsterdam University Medical Centers-location Vrije Univeristeit, Department of Human Genetics; Center for Neurogenomics and Cognitive Research, Department of Functional Genomics, Vrije Universiteit and Amsterdam Neuroscience Neurodegeneration, Amsterdam, Netherlands.

## Abstract

Kufs disease/CLN4 is an autosomal dominant neurodegenerative disorder caused by unknown mechanisms through Leu^115^Arg and Leu^116^Δ mutations in the DNAJC5 gene that encodes the synaptic vesicle co-chaperone cysteine string protein α (CSPα/DNAJC5). To investigate the disease mechanisms in vivo, we generated three independent mouse lines overexpressing different versions of CSPα/DNAJC5 under the neuron-specific Thy1 promoter: wild-type (WT), Leu^115^Arg, and Leu^116^Δ. Mice expressing mutant Leu^115^Arg CSPα/DNAJC5 are viable but develop motor deficits. As described in patients with Kufs disease, we observed the pathological lipofuscinosis and intracellular structures resembling granular osmiophilic deposits (GRODs) in the mutant but not in the WT transgenic lines. Microglia engulf lipofuscin and lipofuscin-containing neurons. Notably, conventional or conditional knockout mice lacking CSPα/DNAJC5 did not exhibit any signs of increased lipofuscinosis or GRODs. Our novel mouse models provide a valuable tool to investigate the molecular mechanisms underlying Kufs disease/CLN4. DNAJC5 mutations cause neuronal lipofuscinosis through a cell-autonomous gain of a pathological function of CSPα/DNAJC5.

## Teaser

Pathological function of a mutated molecular co-chaperone causes adult-onset autosomal dominant neuronal ceroid lipofuscinosis.

## INTRODUCTION

Neuronal ceroid lipofuscinosis are inherited neurodegenerative diseases that normally appear in children and young individuals, encompassing thirteen distinct genetic diseases, CLN1-14 (*1*). The patients generally suffer from severe neurological symptoms such as seizures, dementia, blindness, and motor disorders that lead to premature death (*1*). Typically, those mutations are loss-of-function mutations in genes that encode a variety of proteins involved in lysosomal function, such as proteins involved in the trafficking of lysosomes, lysosomal integral membrane proteins, and lysosomal enzymes (*2*–*4*). Autosomal-dominant adult-onset neuronal ceroid lipofuscinosis (CLN4), originally known as Kufs disease, is a severe form of neuronal ceroid lipofuscinosis with a symptomatic onset between 25 and 46 years and characterized by generalized seizures, movement disorders, cognitive deterioration, and progressive dementia (*5*–*9*). The neuronal accumulation of autofluorescent material and the ultrastructural detection of granular osmiophilic deposits (GRODs) are the major neuropathological hallmarks of the disease (*5*, *6*). Mutations in *DNAJC5* that encodes the synaptic co-chaperone cysteine string protein α (CSPα/DNAJC5) cause Kufs disease/CLN4 (*10*–*15*).

CSPα/DNAJC5 belongs to the heat shock protein 40 (Hsp40) family of molecular co-chaperones, and it is bound to the external surface of the synaptic vesicle through a string of palmitoylated cysteines. CSPα/DNAJC5 operates through a chaperone complex that includes heat shock cognate 70 (Hsc70) and small glutamine-rich tetratricopeptide-containing protein-A (SGTA) (*16*). Such a trimeric complex acts as a chaperone for the soluble *N*-ethylmaleimide–sensitive factor attachment protein (SNAP) receptor (SNARE) complex, and it is essential to maintain the stability of SNAP25 at synapses (*17*). Neurodegeneration and early lethality occur in *Drosophila* and mice without CSPα/DNAJC5 (*18*, *19*). Synaptic degeneration induced by absence of CSPα/DNAJC5 is associated with the deficit of SNAP25, but the molecular mechanisms are not well understood yet (*17*). Beyond its causative role in Kufs disease/CLN4, CSPα/DNAJC5 has been associated with mechanisms related to other neurodegenerative diseases such as (i) Parkinson’s disease, through the cooperation with α-synuclein as a SNARE complex chaperone (*20*), and (ii) as a mediator of prion-like propagation of key proteins involved in neurodegeneration such as tau, α-synuclein, and transactive response DNA binding protein 43 kDa (TDP-43) (*21*–*25*). In any case, the molecular mechanisms by which the mutant versions of CSPα/DNAJC5 cause Kufs disease/CLN4 are not well understood yet. It has been demonstrated that the two mutations causing Kufs disease/CLN4, Leu^115^Arg and Leu^116^Δ, localized at the cysteine string domain, interfere with normal CSPα/DNAJC5 palmitoylation (*13*, *26*, *27*) and lead to the formation of molecular accumulations. Several scenarios have been considered to explain the pathological mechanisms associated with these accumulations, which include loss- and/or gain-of-function mechanisms. The most generally accepted scenario, a dominant-negative loss-of-function mechanism, considers that the CSPα/DNAJC5 mutant forms act by sequestering and, therefore, suppressing the levels and the function of the endogenous wild-type (WT) form of CSPα/DNAJC5 (*10*, *27*–*29*). Two observations support that view: (i) the reduced levels of CSPα/DNAJC5 detected by immunohistochemistry in patients (*13*) and (ii) the co-oligomerization between WT and mutant forms of CSPα/DNAJC5 (*27*, *29*). In addition, CSPα/DNAJC5 heterozygous knockout (KO) mice are essentially normal, whereas CSPα/DNAJC5 KO mice die early several weeks after birth (*18*, *30*). Translating that scenario to humans, a potential effect of lowering gene dose in heterozygous patients would not be pathological in contrast to a severe loss of CSPα/DNAJC5 via a dominant-negative mechanism (*7*). The gain-of-function scenarios consider different possibilities: (i) the mutant accumulations might cause cellular toxicity by recruiting and sequestering other, so far unknown, key cellular proteins (*27*); (ii) the mutant forms themselves maintain their functional co-chaperone properties, but they undergo a gain of function evidenced by an increased tendency to form oligomers (*31*); and (iii) the pathological phenotypes are due to a hypermorphic mutation that has a toxic effect mediated by an increasing, so far unknown, activity of CSPα/DNAJC5 mutant forms (*32*). Most of the results supporting gain-of-function phenotypes are based on studies derived from human samples or cell culture studies, except a recent study in a *Drosophila* model in which the genetic reduction of endogenous levels of CSPα/DNAJC5 ameliorated the pathological phenotype induced by CSPα/DNAJC5 mutant forms (*32*). Accordingly, the neurotoxicity of the mutants may be at least, in part, due to an impairment of lysosomal function by CSPα/DNAJC5 accumulations (*31*, *32*) that accumulate on prelysosomal endosomes (*32*). Another pathological mechanism proposed for Kufs disease/CLN4 is an aberrant change in the palmitoylation of lysosomal and synaptic proteins caused by a decreased enzymatic activity of the depalmitoylating enzyme palmitoyl-protein thioesterase (PPT1/CLN1) (*28*). In any case, although the investigation of pathological mechanisms and development of therapeutical approaches for others CLNs have enormously benefited from mammalian animal models, genetic models in mammals to investigate Kufs disease/CLN4 mechanisms are now not available.

Here, we have generated independent transgenic mouse lines that express in neurons CSPα/DNAJC5: WT, Leu^115^Arg, or Leu^116^Δ. We have observed that the transgenic mice expressing the mutant forms clearly develop the typical autofluorescent punctate staining characteristic of lipofuscinosis and GROD-like structures previously described in patients. In addition, we detected lipofuscin engulfing by microglia in mutant mice. In contrast, we did not find lipofuscinosis in CSPα/DNAJC5-WT transgenic mice. In addition, lipofuscinosis turned out to be absent in conventional CSPα/DNAJC5 KO mice, and, furthermore, it was not detected in aged conditional CSPα/DNAJC5 KO lacking the protein specifically in glutamatergic neurons. Our results indicate that the lack of CSPα/DNAJC5 does not cause lipofuscinosis in mice, whereas the presence of pathological mutant forms in neurons causes lipofuscinosis by a cell-autonomous mechanism. We conclude that the main pathological mechanism driving Kufs disease/CLN4 is likely mediated by an aberrant gain of function of CSPα/DNAJC5 mutants that leads to pathological interactions with so far unknown proteins and not by a dominant-negative mechanism suppressing the function of endogenous normal CSPα/DNAJC5.

## RESULTS

### Expression of CLN4 CSPα/DNAJC5 mutant forms in transgenic mouse brains

**Fig. 1.**
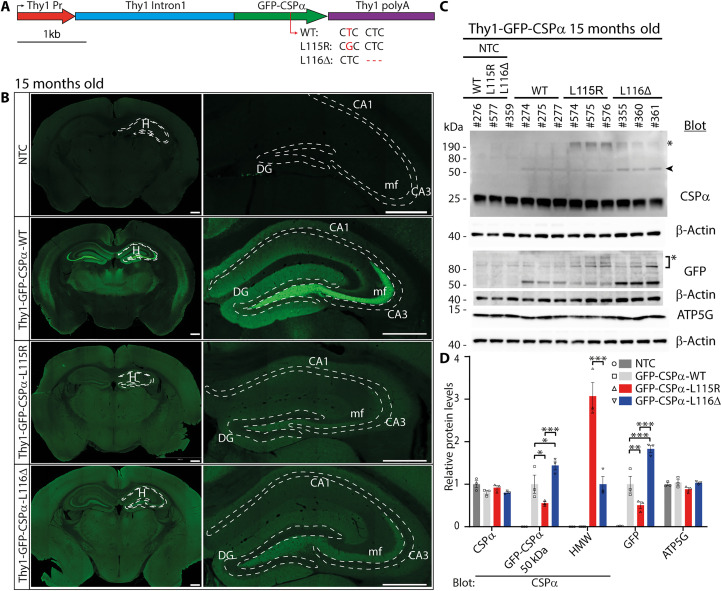
Transgenic expression of WT and CLN4 mutant forms of CSPα/DNAJC5 in mouse brain. (**A**) Genetic strategy to drive the neuronal expression of green fluorescent protein (GFP)–CSPα–WT and CLN4 mutants GFP-CSPα-L115R and GFP-CSPα-L116Δ under the neuron-specific promoter Thy1. (**B**) Representative epifluorescence images of GFP immunostaining demonstrate widely distributed expression of all transgenes in brain in 15-month-old mice. General stronger transgene expression of GFP-CSPα-WT especially evident at hippocampal mossy fibers. No signal is detected in non-transgenic control (NTC) mice. Scale bars, 500 μm. H, hippocampus; CA1 and CA3, Hippocampal cornu Ammonis regions 1 and 3; DG, Dentate gyrus; mf, mossy fibers. (**C**) Transgenic proteins detected by Western blot of hippocampal extracts from 15-month-old mice. Numbers indicate mouse ID number. Top blot: Endogenous CSPα/DNAJC5 is detected in all samples, while a band corresponding to GFP-tagged CSPα/DNAJC5 (arrowhead, 50 kDa) appears in transgenic samples but not in NTC. High molecular weight species [asterisk (*)] are detected in mutant transgenic samples, especially in L115R mutant. GFP signal is only detected in transgenic samples. Nonspecific band due to GFP antibody [asterisk (*)]. (**D**) Levels of selected hippocampal proteins. Relative protein levels normalized to non-transgenic mouse lines, except for GFP quantification that was normalized to GFP levels of the GFP-CSPα-WT transgenic line. Two-way analysis of variance (ANOVA) with Tukey’s post hoc test (*P < 0.05; **P < 0.01; ***P < 0.001). Quantitative data are available in table S1.

Because CLN4 is an autosomal-dominant disease, we considered to drive the expression of CLN4 CSPα/DNAJC5 mutant forms in neurons over a WT background as a potentially useful strategy to generate CLN4 animal models. We used the Thy1 promoter to induce the transgenic expression in neurons (Fig. 1A). The whole-genome sequencing provided details of the number of transgene copies and insertion sites for every mouse line. Two transgene copies were inserted in tandem in the line green fluorescent protein (GFP)–CSPα–L115R by replacing 15 kb of genomic DNA, while only one copy was inserted in the line GFP-CSPα-WT. The line GFP-CSPα-L116Δ also presented one single copy associated to a 25-Mb genomic inversion (fig. S1). Apparently, the insertion of the transgenes did not disrupt any known gene. In addition, six of the eight genes located at the surrounding areas of the transgenes insertions areas were genes not involved in the function of the adult central nervous systems: (i) *Gm33852* and *Gm31086* are predicted long noncoding RNAs according to Mouse Genome Informatics (MGI) database; (ii) *Gm13555* is a predicted pseudogene detected at low levels in the nervous system of the mouse embryo and in newborns, but not in the adult according to MGI database; and (iii) *Or5t16*, *Or8h10*, and *Or5t17* encode olfactory receptors. The other two genes, *Tank* and *Scl4a10*, are expressed in the adult nervous system. *Tank* encodes tumor necrosis factor receptor–associated factor family member–associated nuclear factor κB activator, a scaffolding protein that acts as a negative regulator of proinflammatory cytokine production induced by Toll-like receptor signaling. *Tank* KO mice often suffer from fatal immune complex-mediated renal failure, and they might have an enhancement in anxiety-related behavior towards novel stimuli (*33*, *34*). *Scl4a10* encodes a Na^+^-coupled Cl-HCO_3_^−^ exchanger expressed in choroid plexus epithelial cells and in neurons and its disruption markedly reduces brain ventricle volume and protects against fatal epileptic seizures in mice (*35*). The 25-Mb inversion associated with the Thy1-GFP-CSPα-L116Δ transgene insertion did not include *Tank1* and did not disrupt *Scl4a10* that was fully included within the inverted genomic segment (fig. S1D). The expression of the GFP-CSPα-WT, GFP-CSPα-L115R, and GFP-CSPα-L116Δ transgenes was detected in multiple regions of the brain, and we selected three transgenic mouse lines with comparable expression patterns in hippocampus and cortex (Fig. 1B). We studied transgene expression patterns in 8- and 15-month-old mice (Fig. 1 and fig. S2) to find similar results at both ages. The expression of the GFP-CSPα-WT transgene turned out to be better detectable than the expression of the CLN4-mutant forms, especially when compared with the expression of CSPα-L115R (Fig. 1B). This was, for example, evident at the hippocampal mossy fibers (Fig. 1B). To further investigate protein expression levels, we analyzed by immunoblot the levels of endogenous CSPα/DNAJC5 and transgenic proteins (Fig. 1C). The levels of endogenous CSPα/DNAJC5 were similar in the three transgenic lines and in the non-transgenic controls (NTCs; Fig. 1C). We did not observe differences in the levels of the C-subunit of mitochondrial adenosine triphosphate synthase (ATP5G), which is a characteristic of lipofuscin bodies (*36*–*38*). The expression levels of the transgenic proteins, however, varied among the three lines, with GFP-CSPα-L115R having the lowest and GFP-CSPα-L116Δ having the highest expression. In CLN4 mutants, especially in the L115R mutant, we found the presence of high molecular weight oligomers of CSPα/DNAJC5, likely corresponding to the CSPα/DNAJC5 accumulations previously described (Fig. 1C and fig. S2) (*26*, *27*, *29*, *31*, *32*).

### Neuronal lipofuscinosis in CLN4 transgenic mice

**Fig. 2.**
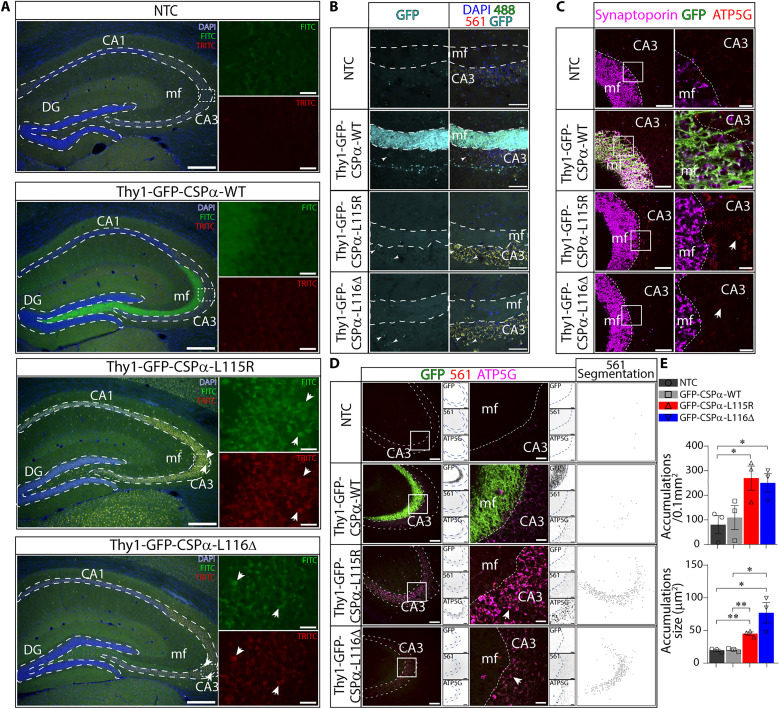
Neuronal lipofuscinosis in Thy1-GFP-CSPα-L115R and Thy1-GFP-CSPα-L116Δ transgenic mice. (**A**) Representative merged epifluorescence images of mouse hippocampal slices from NTC and GFP-CSPα-WT, GFP-CSPα-L115R, and GFP-CSPα-L116Δ transgenic mice at 15 months old stained with 4′,6-diamidino-2-phenylindole (DAPI) (blue). Using the fluorescein isothiocyanate (FITC) filter set, the overlapping fluorescence signals (green) coming from GFP fluorescence and autofluorescence are collected. By using the tetramethylrhodamine isothiocyanate (TRITC) filter set, only autofluorescence signal is collected (red and yellow). Autofluorescence puncta are evident in transgenic mice expressing CLN4 CSPα/DNAJC5 mutations, especially at the pyramidal cell layers (arrows). Scale bars, 200 μm. (**B**) Merged confocal images of mossy fibers at the CA3 region in hippocampal slices stained with antibodies against GFP (cyan) and DAPI (blue). Autofluorescence signal (488- and 561-nm laser channels) (yellow) is evident in pyramidal neurons at the CA3 region in CLN4 mutants. Scale bars, 50 μm. (**C**) Confocal images from hippocampal sections stained with anti-ATP5G (red), anti-GFP (green), and anti-synaptoporin (magenta) antibodies. GFP only detected in GFP-CSPα-WT mice at mossy fibers. ATP5G clearly detected at CA3 postsynaptic cells in GFP-CSPα-L115R mice. Scale bars, (left) 50 μm and (right) 10 μm. (**D**) As in (C) plus the collection of autofluorescence through 561-nm laser channel. Autofluorescence and ATP5G signal detected in CLN4 mutants. Small square panels located at the right side of every individual panel, respectively, display, from top to bottom: the GFP, the 561-nm laser channel, and the ATP5G signals. White panels at the right show images resulting after mask segmentation of 561-nm laser channel used for quantification of autofluorescent spots (see Material and Methods). Scale bars, (left) 100 μm and (right) 10 μm. (**E**) Significant differences are observed in accumulations size and density. Means ± SEM. *n* = 3 animal/genotype. *P < 0.05; **P < 0.01, unpaired *t* test. Quantitative data available in table S1.

Lipofuscinosis is the major hallmark in the brain of patients with Kufs disease/CLN4, which is classically found as punctate structures of autofluorescent storage material (AFSM) (*5*, *7*, *36*). We focused on hippocampal slices to investigate with fluorescence microscopy whether the transgenic expression of CLN4 mutants induced the appearance of AFSM (Fig. 2). AFSM is normally excited between 320 and 480 nm with an emission spectrum between 460 and 630 nm (*36*). We used the fluorescein isothiocyanate (FITC) and tetramethylrhodamine isothiocyanate (TRITC) filter sets to examine fluorescence from hippocampal slices. Using the FITC filter set, we detected the prominent green fluorescence of GFP at mossy fibers in GFP-CSPα-WT and a weaker general signal in GFP-CSPα-L115R and GFP-CSPα-L116Δ transgenics (Fig. 2A). Only in CLN4 mutants, a punctate pattern of autofluorescence was evident being especially strong in the GFP-CSPα-L115R transgenics at the CA3 region (Fig. 2A). Such a punctate pattern was also evident using the TRITC filter (Fig. 2A), suggesting that those structures could be AFSM. We next proceeded to a closer examination at the CA3 region labeling with anti-GFP antibodies (Fig. 2, B and C). In the CLN4 mutants, in which the GFP signal was nevertheless very low (Fig. 2B), the punctate ATP5G staining was clearly detected at the soma of CA3 pyramidal neurons as perinuclear structures (Fig. 2C) but not at the mossy fiber terminals stained with antibodies against the synaptic vesicle protein synaptoporin (Fig. 2C). Some of those punctate structures co-localized with the vestigial GFP staining in the GFP-CSPα-L115R transgenic mice (Fig. 2B, arrowheads). To further investigate whether those structures corresponded with AFSM, we labeled the slices with antibodies against ATP5G and found again a clear signal in the CA3 region, especially in the GFP-CSPα-L115R transgenic mice (Fig. 2D). The ATP5G signal was absent at the mossy fibers in all the mouse lines, including the GFP-CSPα-WT line (Fig. 2D). The localization of ATP5G (Fig. 2D) was highly correlated with the autofluorescence signal detected through the 561-laser line channel [Pearson’s correlation coefficient (*r*) of 0.61; see table S1]. This was consistent with the notion that ATP5G, likely with other mitochondrial proteins, yields the autofluorescence originated from AFSM.

Notably, the prominent GFP signal in mossy fibers in the GFP-CSPα-WT transgenic did not translate into obvious autofluorescence in the mossy fiber terminals or in the soma of CA3 pyramidal neurons. In addition, we could not detect any substantial autofluorescence in the non-transgenic mice used as negative controls. The signs of lipofuscinosis were more evident in the GFP-CSPα-L115R than in the GFP-CSPα-L116Δ transgenic mice and in mice older than 4 months, with the highest accumulation in mice aged 15 months old (fig. S3). In addition, to avoid any confounding effect of fluorescence signals, we demonstrated the existence of ATP5G accumulations by immunohistochemistry and bright field microscopy (fig. S4).

Next, to investigate the origin of autofluorescence, we searched for the presence of granulovacuolar degeneration bodies (GVBs) that are neuron-selective lysosomal structures characterized by the accumulation of a distinctive subset of proteins within a dense core, typically induced by tau pathology (*39*). We investigated whether these structures might contribute to the pathological changes associated with CLN4 mutations. Sections were immunolabeled with antibodies against casein kinase 1 δ (CK1δ) and phosphorylated protein kinase R–like endoplasmic reticulum kinase (pPERK) that, as previously reported, are useful markers to detect GVBs in the human brain, as well as in mouse brain and neuronal cultures (*39*). Neither CK1δ nor pPERK immunolabeling showed GVBs in any of the genotypes (fig. S5). Furthermore, immunolabeling with an antibody against the lysosomal and GVB membrane marker lysosomal integral membrane protein-2 did not reveal clear differences between genotypes (fig. S5). Therefore, the punctate autofluorescence phenotype of mutant GFP-CSPα mice was not reflected by an obvious lysosomal phenotype or related to the presence of GVBs. We concluded that CSPα/DNAJC5 CLN4 mutations do not induce hippocampal GVB formation. To further search for lysosomal alterations, we measured the activity and the protein levels of the lysosomal aspartic-type protease cathepsin D. This enzyme is involved in lysosomal-mediated degradation of unfolded or oxidized protein aggregates associated to neurodegenerative diseases (*40*) and slightly increased in CLN4 (*28*), but we did not find any significant differences between mutant mice and controls (fig. S6). In addition, we used antibodies against α-synuclein (fig. S7) and hyperphosphorylated tau (fig. S8) to label hippocampal slices and identify aggregates commonly associated with neurodegeneration (*41*–*43*), but the staining pattern appeared identical across all genotypes. Similarly, the staining with antibodies against caspase3, which we used to search for signs of cell death, also showed no variation among the genotypes (fig. S9).

Together, these observations suggest that the autofluorescence is specifically detected only in CLN4 mutant transgenic mice, even under a rather low level of transgenic protein expression compared to the levels of endogenous CSPα/DNAJC5. Autofluorescence likely corresponds to age-dependent pathological lipofuscinosis at the neuronal soma with accumulation of a mitochondrial marker, rather than lysosomal proteins. To further investigate the origin of the autofluorescence, we next used electron microscopy to explore ultrastructural intracellular details.

### GROD-like structures in neurons of CLN4 transgenic mice

**Fig. 3.**
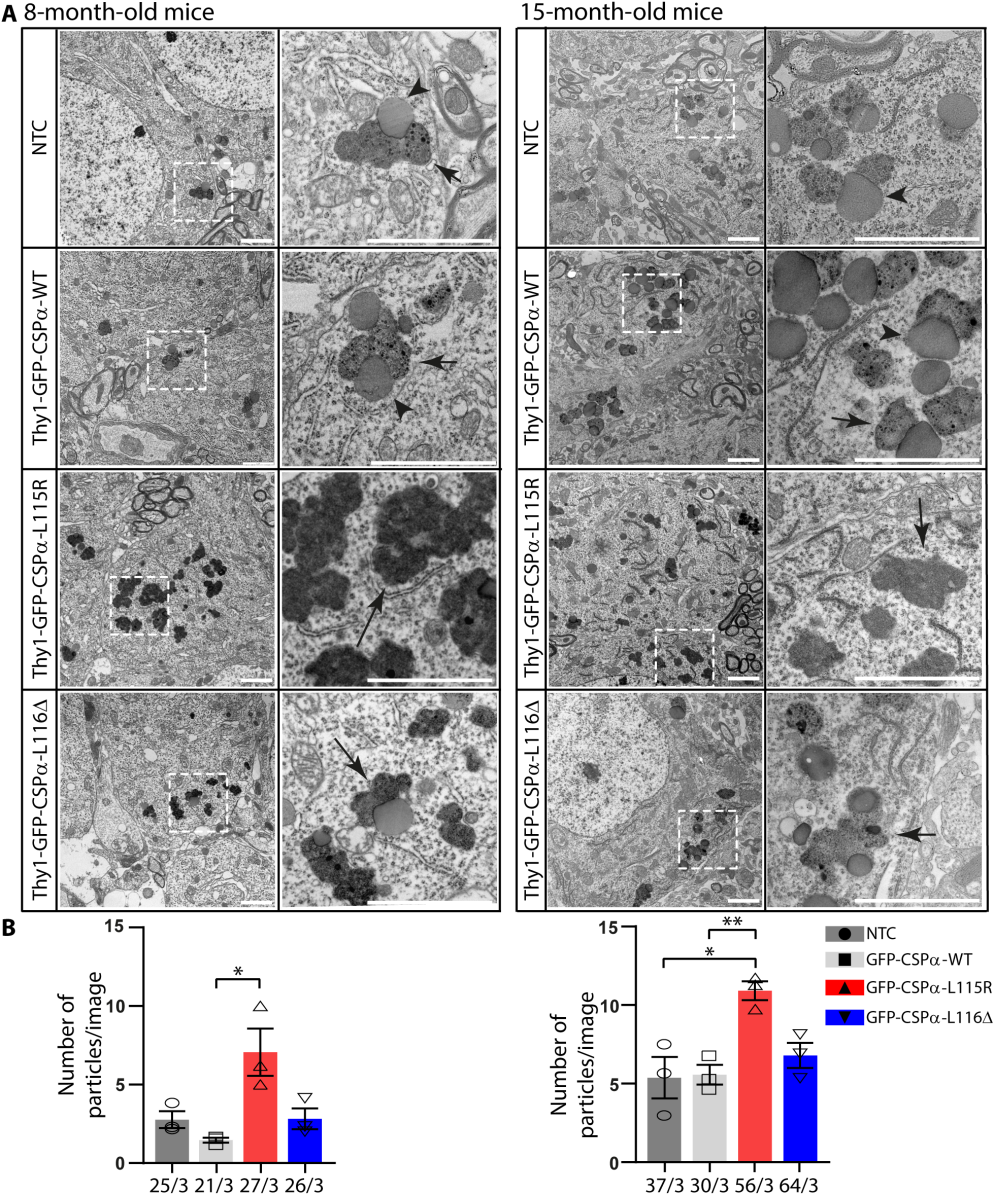
GROD-like structures in the somata of CA3 pyramidal neurons of CLN4 transgenic mice at 8 and 15 months age. (**A**) Transmission electron microscopy analysis reveals normal lipofuscin characterized by lipid droplets associated to rather clear structures with dark puncta (short arrows) in control mice (NTC and Thy1-GFP-CSPα-WT) in contrast to dark GROD-like structures (long arrows) found in mutant mice (Thy1-GFP-CSPα-L115R and Thy1-GFP-CSPα-L116Δ). Pictures at right columns are magnification of selected areas at the left columns. (**B**) Lipofuscin and GROD-like structures were all quantified and plotted as particles per image revealing an increased number in the GFP-CSPα-L115R mutant at both ages studied (8 months on the left and 15 months on the right). Three animals were studied per genotype. *P < 0.05; **P < 0.01, unpaired *t* test. Scale bars, 2 μm.

We examined the soma of hippocampal pyramidal neurons at the CA3 region in 8- and 15-month-old mice of different phenotypes (Fig. 3A). In 8 months, old NTC mice, we found cytosolic round compound structures generally composed by a translucent sphere, likely a lipid droplet, attached to darker membrane-bound structures filled with a moderated electron dense material including linear profiles and some very dark dots (Fig. 3A, left). The same type of structures was found in samples from the GFP-CSPα-WT mice. In contrast, we did not find those structures in GFP-CSPα-L115R mice. Instead, the structures that we found in these mutant mice were much more electron dense, apparently formed by accumulated material and with less lipid droplets associated to them. High–electron density structures may indicate a high concentration of macromolecules or higher binding of heavy metals such as osmium. The structures strongly resembled the GRODs found in patients with Kufs disease/CLN4 (*5*, *6*, *13*, *14*, *44*), so we named them GROD-like structures. One possibility is that the structures found in the mutants were not pathological inclusions, but they reflected, instead, the advanced stage of an otherwise physiological age-dependent accumulation of lipofuscin that should be found in aged control mice. To further investigate this possibility, we analyzed aged 15-month-old mice (Fig. 3A, right). The results were similar to those found in younger 8-month-old mice. We could not find GROD-like structures in NTC and GFP-CSPα-WT transgenic aged mice in contrast to the evident presence in GFP-CSPα-L115R transgenic mice at both ages (Fig. 3A, right). This observation suggests that GROD-like structures do not appear because of physiological aging in control mice, and they are instead a specific feature of the CLN4 transgenic mutant mice. Although the GROD-like structures were most evident in GFP-CSPα-L115R mice at both ages studied, they were also detected in GFP-CSPα-L116Δ mice but to a lesser extent (Fig. 3A). The number of lipofuscin particles, including GROD-like structures, was significantly higher in GFP-CSPα-L115R transgenic mice compared to that in controls, but not in GFP-CSPα-L116Δ transgenic mice (Fig. 3B).

### Microglia engulfing of lipofuscin in CLN4 mutant mice

**Fig. 4.**
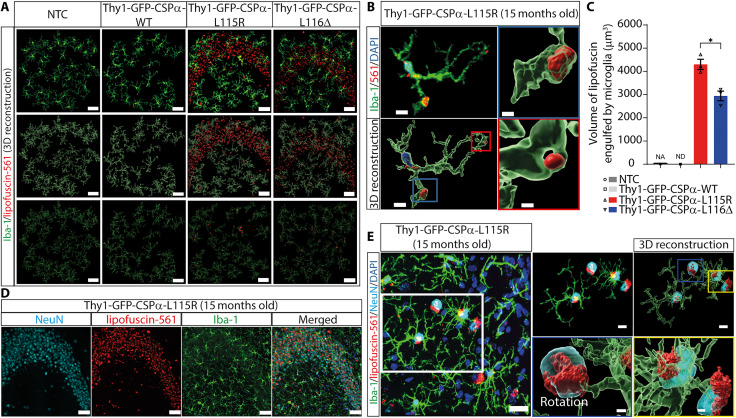
Microglia engulf lipofuscin and neurons in Thy1-GFP-CSPα-L115R and Thy1-GFP-CSPα-L116Δ transgenic mice. (**A**) First row: Representative images of immunostaining of microglia (Iba-1, green) and lipofuscin (red, autofluorescence at 561 nm using the TRITC filter) at the CA3 region of Thy1-GFP-CSPα-L115R of 15 months old mutant mice. Second row: Imaris three-dimensional (3D) reconstruction. Third row: Imaris 3D reconstruction of lipofuscin engulfed by microglia. Scale bars, 50 μm. (**B**) Imaris 3D reconstruction of one microglia cell of Thy1-GFP-CSPα-L115R mutant mice at 15 months old showing engulfed lipofuscin particles by microglia. Scale bars, 8 and 2 μm (red and blue boxes). (**C**) Quantification of volume of lipofuscin engulfed by microglia in control (NTC) and GFP-CSPα-WT, GFP-CSPα-L115R, and GFP-CSPα-L116Δ transgenic mice. Data were presented as means ± SEM; two-way ANOVA with Tukey’s post hoc test *P < 0.05, *n* = 3 mice per group, three images per mouse. At least 25 microglia cells per mouse were analyzed to get the processes length of microglia data. NA, not applicable; ND, non-detectable. (**D**) Immunostaining of microglia (Iba-1, green), lipofuscin (red, autofluorescence at 561 nm), neurons (NeuN, cyan) and nuclei (DAPI, blue) of 15 months old Thy1-GFP-CSPα-L115R mice. Scale bars, 50 μm. (**E**) Representative images of Imaris 3D reconstruction showing the close interaction between microglia and neurons with lipofuscin (blue square) and neurons with lipofuscin that is engulfed by microglia (yellow square) in Thy1-GFP-CSPα-L115R mice. Scale bars, 20 and 10 μm (white box) and 1 μm (blue and yellow boxes). Part of the dataset used to generate (A) and (C) (15 month-old mice data) was also used for figs. S12 and S13. NTC and Thy1-GFP-CSPα-WT dataset (both from 15-month-old mice) are the same in this figure and in fig. S12.

Because microglia alterations have been previously reported in tissue of CLN4 (*45*) and in mouse models of NCLs (*46*–*49*), we investigated the status of microglia in CLN4 mutant transgenic mice. Microglia are continuously extending and retracting their processes to survey the brain (*50*, *51*) and target damaged cellular structures to envelop them, a process normally associated with changes in microglia morphology (*52*, *53*). We used antibodies against the ionized calcium-binding adapter molecule 1 (Iba1) to study microglia with confocal microscopy at the CA3 region of GFP-CSPα-WT, GFP-CSPα-L115R, and GFP-CSPα-L116Δ transgenic and non-transgenic mice (Fig. 4). Microglia were equally abundant in CLN4 mutant and control but surrounded by autofluorescence storage material in CLN4 mutant mice (Fig. 4A). We used Imaris three-dimensional (3D) reconstruction to quantify the morphological properties of microglia, focusing on (i) the ramification of their processes and the size of cell bodies (Fig. 4A) and to (ii) the spatial relationship between microglia and lipofuscin visualized as autofluorescence storage material (Fig. 4, A and B). First, we measured the area of the cell body, the total area occupied by microglia, and the processes length per cell in GFP-CSPα-L115R (fig. S10) and GFP-CSPα-L116Δ mice (fig. S11) and compared them with the values obtained from GFP-CSPα-WT and NTCs (both 15 months of age) at different ages (1, 4, 8, and 15 months of age). We found only a moderate increase in cell body size in microglia of GFP-CSPα-L115R mice and a decrease in the length of the microglial processes of GFP-CSPα-L115R and GFP-CSPα-L116Δ mice (figs. S10 and S11).

These morphological changes are somehow like those occurring in the interleukin 1β–releasing microglia (*52*, *53*). In addition, we observed that, although most of autofluorescence storage material was located around microglia (Fig. 4A and figs. S12 and S13), there were detectable autofluorescence particles within or engulfed by microglial processes in CLN4 mutants (Fig. 4B), being more prominent in GFP-CSPα-L115R than in GFP-CSPα-L116Δ mice at 8 and 15 months of age (figs. S12 and S13).

**Fig. 5.**
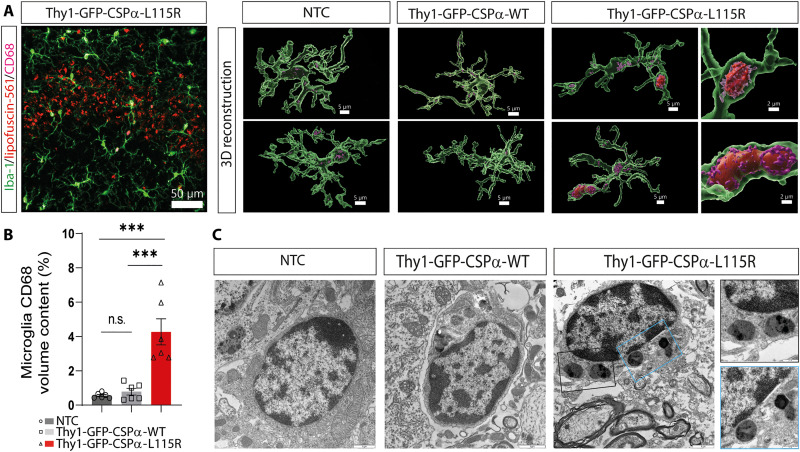
Microglial phagocytosis of lipofuscin in Thy1-GFP-CSPα-L115R at 15 months. (**A**) Immunofluorescence staining in the CA3 region of the hippocampus for Thy1-GFP-CSPα-L115R mice at 15 months of age. Microglia are labeled with Iba1 (green), lipofuscin autofluorescence is detected in red (using the TRITC filter), and the CD68 phagocytic marker is in magenta. Scale bar, 50 μm. The rightmost column of (A) displays representative images of 3D reconstructions using Imaris software, showcasing microglia together with CD68 and lipofuscin in control (NTC), Thy1-GFP-CSPα-WT, and Thy1-GFP-CSPα-L115R mice at 15 months. Scale bars, 5 μm. The zoom-in images highlight microglial activation in Thy1-GFP-CSPα-L115R mice. Scale bars, 2 μm. (**B**) Quantification of the percentage of microglial CD68 volume content, revealing a significant increase in CD68 content in Thy1-GFP-CSPα-L115R mice, compared to controls. (**C**) Representative electron microscopy images of microglia. Notably, microglia in Thy1-GFP-CSPα-L115R mice exhibit a darker appearance and phagocytosed lipofuscin particles. Data were presented as means ± SEM; one-way ANOVA with Tukey’s post hoc test (***P < 0.001). *n* = 3 mice per group, two images per mouse.

**Fig. 6.**
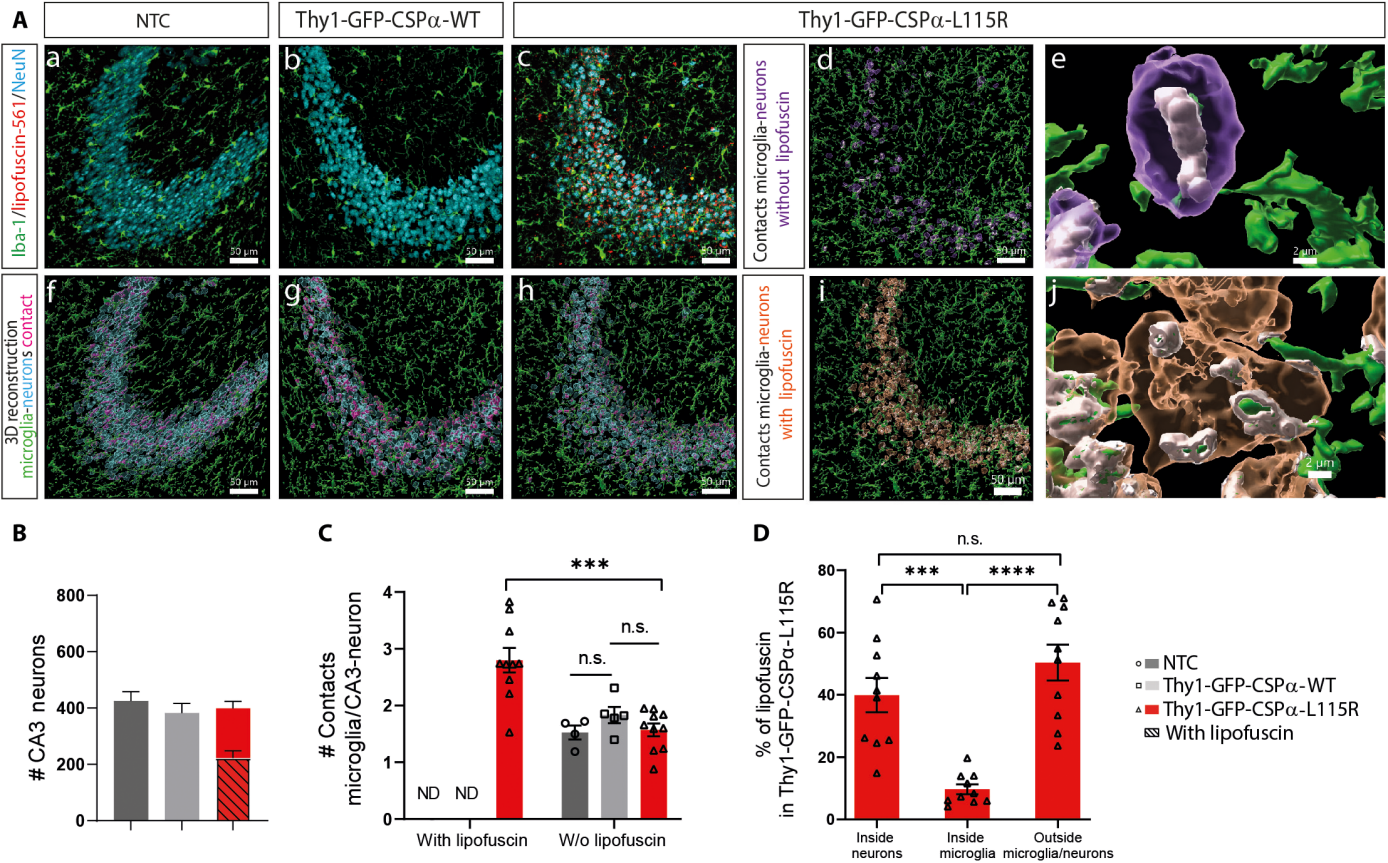
Microglia-neuron interaction in Thy1-GFP-CSPα-L115R transgenic mice at 15 months. (**A**) Microglia (Iba-1, green), neuron (NeuN, cyan), and lipofuscin (red, autofluorescence at 561 nm using the TRITC filter) immunofluorescence staining at the CA3 region was conducted to investigate microglia-neuron interactions in 15-month-old mice. In the first row, (a), (b), and (c) illustrate control (NTC), Thy1-GFP-CSPα-WT, and Thy1-GFP-CSPα-L115R mice, respectively. In the second row, (d), (e), and (f) present 3D reconstructions, with microglia and neurons. In Thy1-GFP-CSPα-L115R mice, two neuronal subtypes were observed: neurons without lipofuscin deposits (d) and neurons with lipofuscin (i). Scale bars, 50 μm. Zoom-in reconstructions (e and j) highlight the microglia-neuron contacts in both neuronal subtypes. Scale bars, 2 μm. (**B**) The number of CA3 neurons in control (NTC), Thy1-GFP-CSPα-WT, and Thy1-GFP-CSPα-L115R mice at 15 months was quantified. (**C**) The number of contacts between microglia and CA3 neurons was quantified, distinguishing between neurons with and without lipofuscin deposits. (**D**) The percentage of lipofuscin deposits inside neurons, inside microglia, and outside both cell types was determined in the Thy1 GFP-CSPα-L115R mice. Data were presented as means ± SEM; two-way ANOVA with Tukey’s post hoc test (B and C) and one-way ANOVA with Tukey’s post hoc test (D) (***P < 0.001; ****P < 0.0001). n.s., not significant. *n* = 5 mice, two images per mouse for Thy1-GFP-CSPα-L115R, *n* = 5 mice for Thy1-GFP-CSPα-WT, and *n* = 4 mice for NTC. ND, non-detectable.

Because lipofuscin was not detectable in control mice, we could not see microglia engulfing events either. We could not distinguish whether the autofluorescent material engulfed by microglia corresponded to lipofuscin released to the extracellular space or corresponded, instead, to lipofuscin contained within a neuron. In any case, upon labeling neurons with anti-NeuN, we observed microglia embracing neuronal bodies containing lipofuscin, so we concluded that microglia could at least engulf neurons containing lipofuscin, as previously reported (*54*, *55*). We used antibodies against CD68 to stain phagocytic microglia and found increased microglial CD68 content in Thy1-GFP-CSPα-L115R associated with lipofuscinosis uptake that was also evident with electron microscopy (Fig. 5 and fig. S14). At the CA3 region, the number of neurons in Thy1-GFP-CSPα-L115R mice was the same as in controls (Fig. 6, A and B) but ~60% of them contained lipofuscin deposits and microglia preferentially interacted with these neurons (Fig. 6C). Besides being internalized in neurons (~40%) and microglia (~12%), a remarkable amount of lipofuscin (~48%) was located either in other cell types or at the extracellular space (Fig. 6D). We also observed obvious signs of astrocytic reactivity associated with lipofuscinosis (fig. S15) but did not detect any obvious changes in myelinization (fig. S16).

Together, our data reveal that the transgenic expression of mutant forms of CSPα/DNAJC5 in neurons is enough to cause neuronal lipofuscinosis in mice that is similar to the pathological characteristics of Kufs disease/CLN4 in humans (*5*, *6*). These findings identify our mice as unique in vivo models to investigate the molecular basis of lipofuscinosis in Kufs disease/CLN4. A key question arising is which is the mechanism underlying the genesis of lipofuscinosis. CSPα/DNAJC5-mutated forms might induce the pathology by (i) a loss-of-function mechanism as a secondary decrease in the levels of endogenous non-mutated CSPα/DNAJC5, (ii) a hypermorphic gain-of-function mechanism as previously shown in *Drosophila* (*32*), and (iii) a mechanism by which the CSPα/DNAJC5-mutated forms would sequester specific proteins required for an as of yet unknown biochemical pathway that prevents pathological lipofuscinosis. These scenarios do not necessarily exclude one another, and we proceeded to investigate which one of them could be the culprit for pathological lipofuscinosis. Within this context, we characterized the neurological phenotype of the mutant mice.

### Lower mobility and loss of coordination in CLN4 mutant mice

**Fig. 7.**
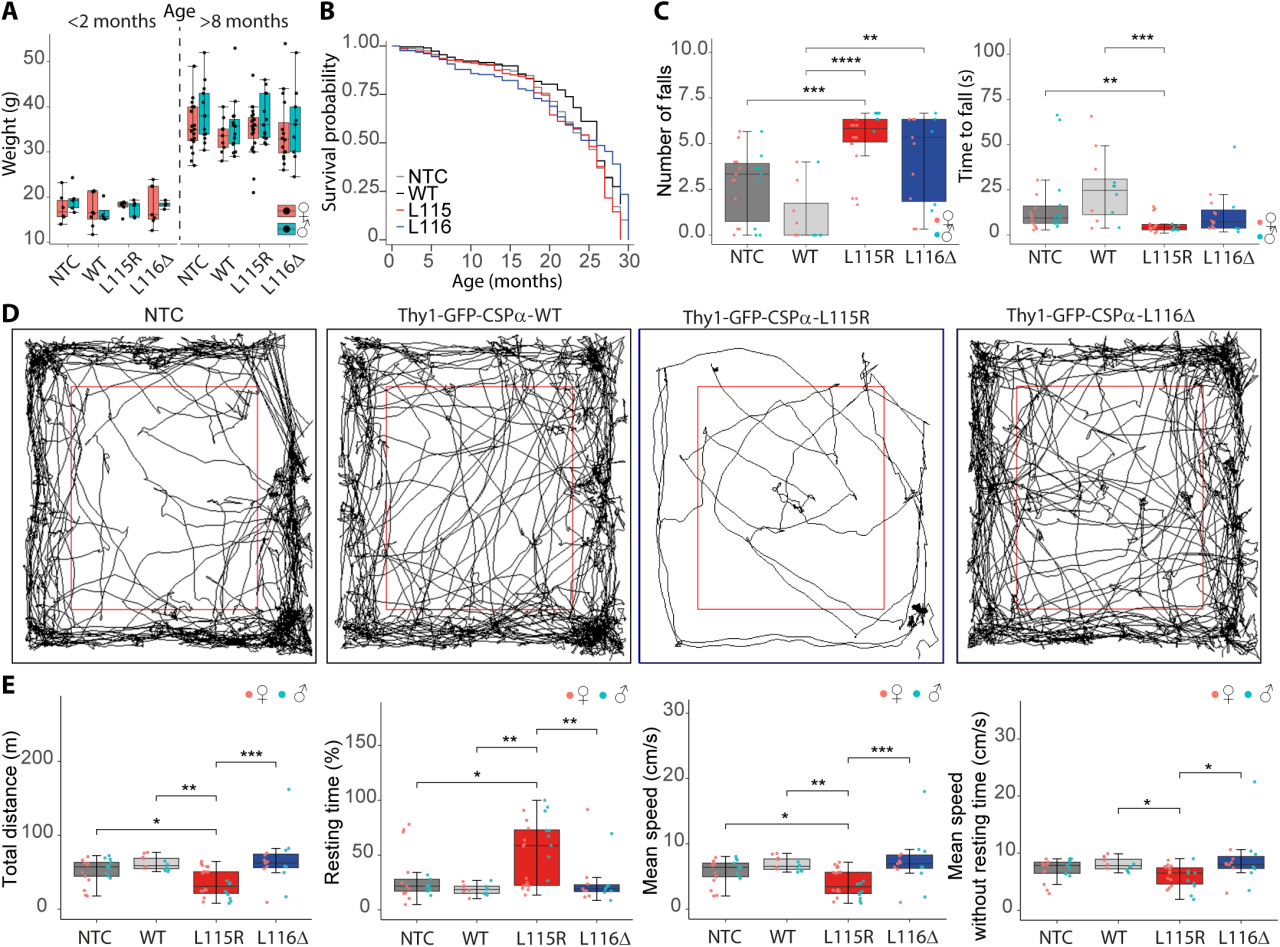
Body weight, survival, and motor phenotype of Thy1-GFP-CSPα transgenic mice. (**A**) Body weights of NTC, Thy1-GFP-CSPα-WT (WT), Thy1-GFP-CSPα-L115R (L115R), and Thy1-GFP-CSPα-L116Δ (L116Δ) mice were assessed in young (<2 months) and older (>8 months) groups. No significant weight differences were observed across genotypes. Sample sizes: young female (NTC, *n* = 6; WT, *n* = 8; L115R, *n* = 7; L116∆, *n* = 7), young male (NTC, *n* = 5; WT, *n* = 4; L115R, *n* = 5; L116∆, *n* = 4), old female (NTC, *n* = 19; WT, *n* = 9; L115R, *n* = 23; L116∆, *n* = 16), and old male (NTC, *n* = 13; WT, *n* = 15; L115R, *n* = 13; L116∆, *n* = 9). (**B**) Survival probability, shown by Kaplan-Meier plots, did not differ significantly among groups (log-rank test with Bonferroni correction). Sample sizes: female (NTC, *n* = 79; WT, *n* = 21; L115R, *n* = 33; L116∆, *n* = 43) and male (NTC, *n* = 55; WT, *n* = 25; L115R, *n* = 37; L116∆, *n* = 34). (**C**) Motor function, measured by rotarod, showed reduced performance in L115R mice, with a higher number of falls compared to NTC and WT (*P* < 0.001 for NTC versus L115R; *P* < 0.0001 for WT versus L115R). Time to fall was also shorter in L115R. Sample sizes: female (NTC, *n* = 12; WT, *n* = 6; L115R, *n* = 15; L116∆, *n* = 10) and male (NTC, *n* = 10; WT, *n* = 6; L115R, *n* = 9; L116∆, *n* = 6). (**D**) Representative open-field tracks indicative of lower mobility in L115R mice. (**E**) Open-field analysis indicated motor deficits in L115R and L116Δ mice, with shorter total distance, longer rest times, and slower speeds compared to NTC and WT. Statistical tests: ANOVA or Kruskal-Wallis with post hoc corrections based on normality (Shapiro-Wilk test). Sample sizes: female (NTC, *n* = 11; WT, *n* = 6; L115R, *n* = 15; L116∆, *n* = 8) and male (NTC, *n* = 10; WT, *n* = 6; L115R, *n* = 9; L116∆, *n* = 7). *P < 0.05; **P < 0.01; ***P < 0.001; ****P < 0.0001.

The CLN4 mutant mice were fertile and did not show any obvious sign of deterioration with age, as for example, a loss of body weight (Fig. 7A) or a decreased survival (Fig. 7B and fig. S17), which was similar in mutant and control mice. To investigate whether the CLN4 mutant mice might have any neurological phenotype, we assessed their performance in the rotarod and analyzed the open-field activity of every mouse line in aged (10 to 15 months of age) animals. The rotarod test was useful to evaluate motor coordination and balance. Mice were placed on a rotating rod that incrementally increased its speed (see Materials and Methods). The number of falls during the first minute was increased in GFP-CSPα-L115R and GFP-CSPα-L116Δ compared to that in GFP-CSPα-WT and NTC mice, while the time before falling off the rod was shorter in GFP-CSPα-L115R mice (Fig. 7C). These data revealed a defect in motor coordination in CLN4 mutant mice that was especially evident in the GFP-CSPα-L115R mice [movie S1 (GFP-CSPα-WT) and movie S2 (GFP-CSPα-L115R)]. Upon falling from the rotarod, GFP-CSPα-WT mice moved to explore the surroundings in contrast to GFP-CSPα-L115R mice that stop moving after falling. Next, to analyze mobility, we monitored free activity in the open-field arena (Fig. 7D) and found decreased motor activity in GFP-CSPα-L115R mice revealed by a shorter total walked distance, a lower mean speed, and a longer resting time, which were especially evident in comparison with the GFP-CSPα-WT mice (Fig. 7E and figs. S18 to S22). Decreased time in the center of the arena and increased time spent close to the wall (thigmotaxis) might be an index of “anxiety-like” behavior (*56*); however, in that respect, we could not detect any differences between mutant and control mice, except that the GFP-CSPα-L115R male mice spent longer time in the periphery and shorter time in the center in comparison to GFP-CSPα-L115R female mice (fig. S22). Nevertheless, because these mice turned out to have lower mobility, we cannot conclude that the preferential permanence in one area is attributable only to a bona fide anxiety-like behavior. In any case, the GFP-CSPα-L115R mice bears a significant motor phenotype that could be triggered by a circuit dysfunction in a brain area involved in motor control in which lipofuscinosis has been found such as the substantia nigra, the striatum, or the cerebellum (fig. S23).

### Long-term genetic removal of CSPα/DNAJC5 does not cause lipofuscinosis

**Fig. 8.**
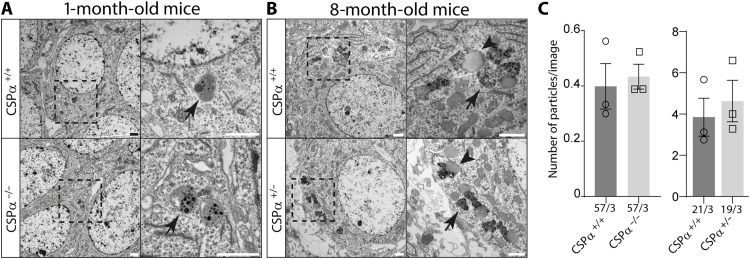
Normal lipofuscin but not GROD-like structures in conventional CSPα/DNAJC5 KO and heterozygous mice. (**A**) Transmission electron microscopy analysis reveals that only normal lipofuscin is rarely found at young CSPα/DNAJC5 KO and WT mice at 1-month of age. (**B**) Normal lipofuscin, but not GROD-like structures, detected at 8-month-old CSPα/DNAJC5 heterozygous mice. (**C**) The number of lipofuscin particles per image in both groups is like control mice; however, the numbers detected in the CSPα/DNAJC5 KO and their controls at 1-month of age. Numbers at the bottom of graph bars indicate number of images/number of mice. Quantitative data are available in table S1.

**Fig. 9.**
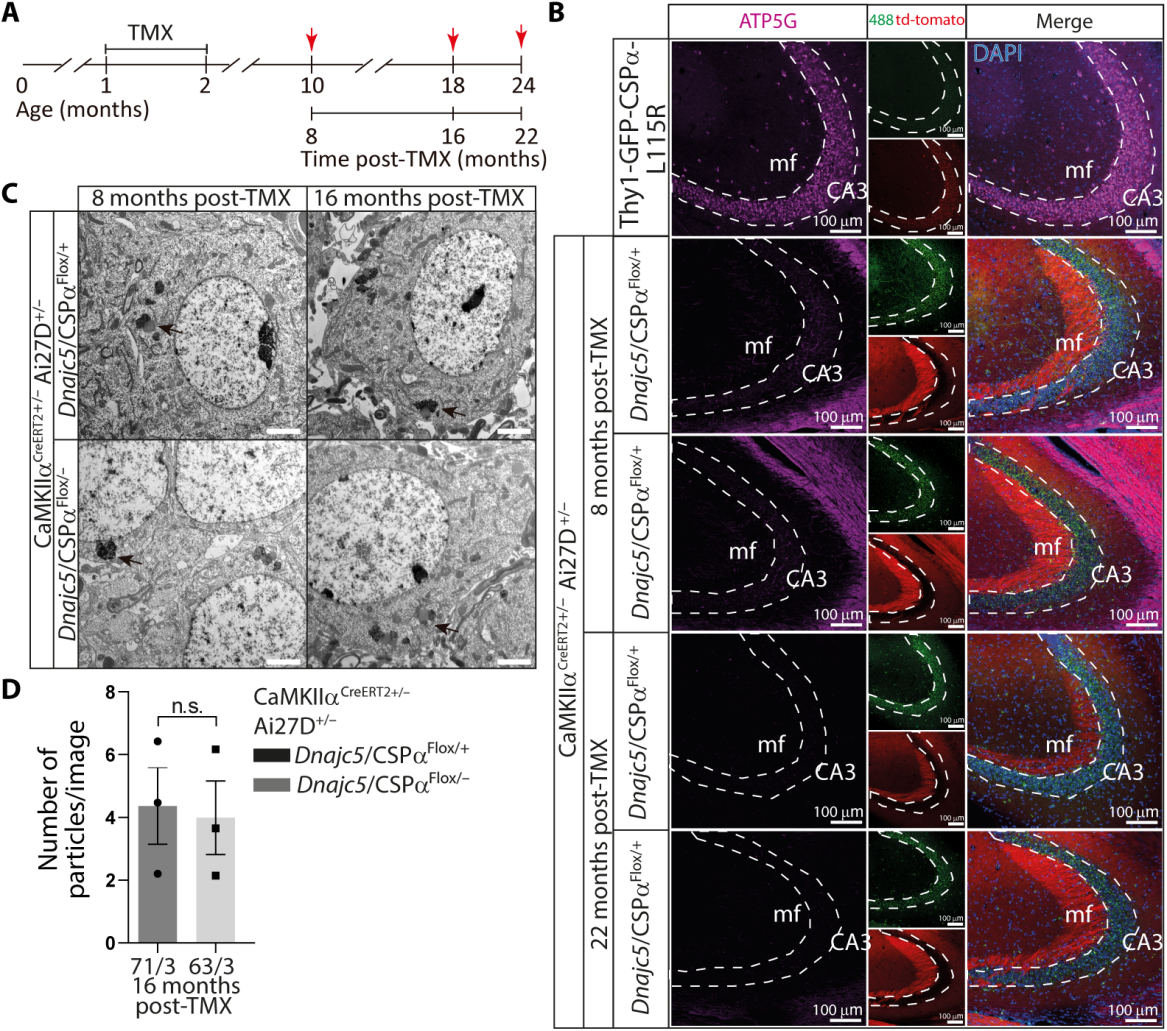
Long-term removal of CSPα/DNAJC5 from glutamatergic neurons does not induce pathological lipofuscinosis. (**A**) Chronogram of the strategy used for the genetic removal of CSPα/DNAJC5 in control (CaMKIIα^CreERT2^:Ai27D:Dnajc5^flox/+^) and experimental (CaMKIIα^CreERT2^:Ai27D:*Dnajc5*^flox/−^) mice. Brains were harvested after feeding 1- or 2-month-old mice with tamoxifen (TMX) (red arrows) for 30 days. Brains of mice fed at 1 month of age were collected at 8-, 16-, and 22-months post-TMX. Brains of mice fed at 2 months of age and collected 8 months later (i.e., at 11 months of age) were used exclusively for electron microscopy analysis. (**B**) Thy1-GFP-CSPα-L115R mice display pathological lipofuscinosis at CA3 pyramidal neurons as demonstrated by the immunolabeling with anti-ATP5G antibodies (magenta) in contrast to mice lacking CSPα/DNAJC5 in hippocampal glutamatergic neurons (CaMKIIα^CreERT2^:Ai27D:*Dnajc5*^flox/−^) and their controls (CaMKIIα^CreERT2^:Ai27D:*Dnajc5*^flox/+^), in which anti-ATP5G staining is negative. GFP is detected in green and the reporter Ai27D (channelrhodopsin 2 fused to td-tomato) in red. Scale bars, 100 μm. (**C**) Transmission electron microscopy analysis only detects normal lipofuscinosis in experimental and control mice at 8 and 16 months after TMX treatment. Scale bars, 2 μm. (**D**) The number of lipofuscin particles per image is similar among the experimental and the respective control mice studied at 8- and 16-months post-TMX. Numbers at the bottom of graph bars indicate number of images/number of mice. Quantitative data are available in table S1.

If lipofuscinosis were induced upon a decrease of CSPα/DNAJC5 levels, then its presence should be evident in the neurons of CSPα/DNAJC5 KO mice. These mice suffer from early lethality and barely reach 1 month of postnatal age, so the oldest mice we could use for this study were 30 days old mice to be compared with CSPα/DNAJC5 WT control littermates. As with the transgenic mice, we used electron microscopy to analyze the somata of pyramidal neurons at the hippocampal CA3 region (Fig. 8). Neither in CSPα/DNAJC5 KO nor in littermate control mice we detected any GROD-like structure. We only detected membrane-bound structures filled with moderate electron dense content including linear profiles and dark spots (Fig. 8, A and C). They were like those found in 8-month-old and 15-month-old NTCs and GFP-CSPα-WT transgenic mice but without lipid droplets attached. Those observations were solid enough to conclude that the mere removal of CSPα/DNAJC5 does not translate into the immediate generation of lipofuscinosis. Nevertheless, those observations did not rule out that a partial, but long term, reduction of CSPα/DNAJC5 levels in 8- and 15-month-old transgenic mutant mice could be the cause of lipofuscinosis. To further explore this possibility, we examined 8-month-old heterozygous CSPα/DNAJC5 KO and WT littermates to investigate whether the gene dosage reduction of CSPα/DNAJC5 constitutively found in heterozygous mice (fig. S24) was enough to build up lipofuscinosis. The electron microscopy analysis, however, only detected the same ultrastructural features previously found in NTCs and GFP-CSPα-WT transgenic mice without any evidence of GROD-like structures (Fig. 8, B and C). Still these results could be explained whether the decrease of ~35% in CSPα/DNAJC5 levels in the heterozygous mice (fig. S24) was not as strong as in the transgenic mutants. To definitely check whether a long-term severe reduction of CSPα/DNAJC5 in adulthood would be enough to induce lipofuscinosis, we searched for signs of lipofuscinosis in CaMKIIα^CreERT2^Ai27D:*Dnajc5*^flox/−^ conditional KO mice by comparing them to CaMKIIα^CreERT2^:Ai27D:*Dnajc5*^flox/+^ littermate control mice (*57*–*59*). The CaMKIIα^CreERT2^:Ai27D:*Dnajc5*^flox/−^ conditional KO mice fed with tamoxifen (TMX) (Fig. 9A) were viable and did not show an obvious increase in morbidity and only an increase in mortality at old ages (61% of CaMKIIα^CreERT2^:Ai27D:*Dnajc5*^flox/−^ survived up to 17 months). The analysis of protein expression in hippocampal sections using fluorescently labeled antibodies clearly demonstrated the absence of CSPα/DNAJC5 at the mossy fibers and at the Schaffer collaterals in 3-month-old mice 1 month after the TMX diet was completed (fig. S25). In addition, the neurons in which Cre-recombinase was active were identified as red fluorescent neurons because of the expression of the reporter td-tomato. Therefore, these mice were ideal to investigate whether lipofuscinosis appears associated to the long-term absence of CSPα/DNAJC5 during adulthood and during aging. We investigated cohorts of mice at 8, 16, and 22 months after the TMX diet was removed (Fig. 9). As expected, CSPα/DNAJC5 was, in general, absent from hippocampal glutamatergic neurons of CaMKIIα^CreERT2^:Ai27D:*Dnajc5*^flox/−^ (fig. S25). We stained hippocampal sections with antibodies against the lipofuscinosis marker ATP5G and did not find any difference between CaMKIIα^CreERT2^:Ai27D:*Dnajc5*^flox/−^ conditional KO mice and controls (Fig. 9B). Furthermore, we examined the CA3 hippocampal region with transmission electron microscopy and did not find any sign of pathological lipofuscinosis such as GROD-like structures (Fig. 9, C and D). Curiously, we found sparse CSPα/DNAJC5 staining in the mossy fibers of aged CaMKIIα^CreERT2^:Ai27D:*Dnajc5*^flox/−^ mice that, in contrast, was not present in younger 3-month-old mice (fig. S25). We interpreted that the presence of CSPα/DNAJC5 came from the mossy-fiber terminals of new granule cells born by adult neurogenesis after TMX discontinuation. As expected, CSPα/DNAJC5 was absent from other neurons that do not undergo adult neurogenesis, such as the CA3 pyramidal neurons, on which our analysis was focused (fig. S25). Together, our results indicate that the absence of CSPα/DNAJC5 by itself does not cause the pathological lipofuscinosis that, on the other hand, is clearly observed in the transgenic mice expressing the mutant forms of CSPα/DNAJC5 causing Kufs disease/CLN4 in humans.

### Lipofuscinosis persists upon lowering CSPα/DNAJC5 levels in transgenic mice

**Fig. 10.**
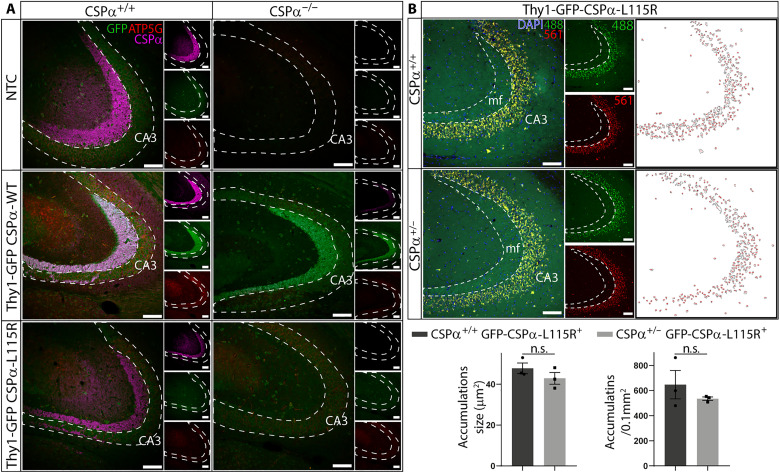
Pathological lipofuscinosis persists upon reducing the endogenous CSPα/DNAJC5 gene dosage. (**A**) One-month-old mice expressing the WT (Thy1-GFP-CSPα-WT) and L115R (Thy1-GFP-CSPα-L115R) transgenic versions of CSPα/DNAJC5 in control and CSPα/DNAJC5 KO backgrounds do not show a prominent staining with anti-ATP5G antibodies (red) at the hippocampal CA3 region. CSPα/DNAJC5 (magenta) is detected in controls, but it is absent in CSPα/DNAJC5 KO mice. Anti-GFP signal detected in green. Scale bars, 100 μm. (**B**) Pathological lipofuscin is detected as a prominent autofluorescent overlapping signal between the signals coming from the 488-nm laser channel (green) and from the 561-nm laser channel (red) at the hippocampal CA3 region of Thy1-GFP-CSPα-L115R under CSPα/DNAJC5 WT and heterozygous backgrounds. Anti-GFP signal detected in green and DAPI in blue. Scale bars, 100 μm. White panels at the right show images resulting after mask segmentation of 561-nm laser channel used for quantification of autofluorescent spots (see Material and Methods). No significant differences (unpaired *t* test) are observed in accumulations size and density. Means ± SEM, *n* = 3 animal per genotype. Quantitative data are available in table S1.

In *Drosophila*, the transgenic overexpression of L115R and L116Δ mutations causes lipofuscinosis accompanied by a prominent neurological phenotype (*32*). The phenotype is ameliorated when the mutation is expressed on a CSPα/DNAJC5 hypomorphic genetic background in which the genetic dose of endogenous WT CSPα/DNAJC5 is significantly decreased. To test that notion in mammals, we bred heterozygous CSPα/DNAJC5 KO mice, which present a reduction in CSPα/DNAJC5 levels (fig. S24), against the GFP-CSPα-L115R transgenic mice. Upon sequential breeding, we generated GFP-CSPα-L115R transgenic mice that were either homozygous or heterozygous CSPα/DNAJC5 KO mice that were studied and compared to their CSPα-L115R transgenic littermates on a CSPα/DNAJC5 WT genetic background (Fig. 10A). As previously explained, the early lethality of CSPα/DNAJC5 KO mice allowed studies only in 1 month old mice that were, at the same time, GFP-CSPα-L115R transgenic and CSPα/DNAJC5 KO mice. In any case, we could not detect any signs of lipofuscinosis in those mice, and the findings were similar to our observations in GFP-CSPα-L115R transgenic mice with a CSPα/DNAJC5 WT background at 1 month of age (fig. S3). The failure to detect lipofuscinosis in these mice, is likely due to the young age of the mice because lipofuscinosis only appears in older mutant CSPα/DNAJC5 transgenic mice, usually beyond 4 months of age (fig. S3). Because of that, we next examined 8 months old GFP-CSPα-L115R transgenic CSPα KO heterozygous mice. As expected, we could detect lipofuscinosis in every GFP-CSPα-L115R transgenic mouse examined. Nevertheless, among those mice, we could not find any differences between CSPα/DNAJC5 KO heterozygous and CSPα/DNAJC5 WT control mice (Fig. 10B). These results indicate that, at least in 8-month-old mice, a genetic dosage reduction of CSPα/DNAJC5 does not ameliorate pathological lipofuscinosis. Overall, our results indicate that neuronal lipofuscinosis does not occur due to a loss-of-function mechanism, but rather because CLN4 mutations transform CSPα/DNAJC5 in a protein with anomalous properties that likely interferes with a cellular process through pathological protein-protein interactions.

## DISCUSSION

We have found that transgenic mice expressing the mutations in *DNAJC5* found in patients (L115R and L116Δ) develop neuronal lipofuscinosis with the pathological hallmarks observed in patients with Kufs disease/CLN4 and that this lipofuscinosis is not due to the lack of CSPα/DNAJC5. Autosomal dominant Kufs disease/CLN4 is an adult-onset neuronal ceroid lipofuscinosis characterized by a progressing devastating neurodegenerative symptomatology affecting individuals after the third decade of life (*7*–*9*). The primary cause of the disease, mutations in the DNAJC5 gene, is well established; however, the underlying cellular and molecular pathological mechanisms remain elusive. Because mutations in heterozygosis cause the disease, we used conventional pronuclear injections to drive the expression of the WT and mutant versions of *Dnajc5* fused to GFP on a genetic background expressing normal levels of endogenous CSPα/DNAJC5. We used the neuronal specific promoter Thy1 that normally produces a variegated expression in neuronal subpopulations with a moderate expression level ideal to avoid aberrant unphysiological effects caused by excessive overexpression (*60*). The transgenic mice were viable and, especially the L115R mutants, suffered from deficits in mobility and coordination compared to controls (Fig. 7 and figs. S18 to S22). We focused our attention on the hippocampus and the cerebral cortex as brain areas in which the transgene expression was detected by GFP fluorescence or by immunofluorescence using antibodies against GFP (Fig. 1). The transgenic WT version of CSPα/DNAJC5 turned out to have a much stronger hippocampal expression, especially in the mossy fibers, compared with the mutant versions, which, in general, were expressed at lower levels (Fig. 1B). These differences may reflect that the mutant proteins display either low stability and/or undergo conformational changes or accumulation that decreases GFP fluorescence and/or masks the detection by antibodies. Supporting this notion, it has been reported that palmitoylated monomers of CSPα/DNAJC5 mutants are short-lived compared to WT CSPα/DNAJC5, suggesting that the mutants could have a faster rate of depalmitoylation and/or they are consumed faster in a time-dependent manner into high molecular weight accumulations (*61*). The transgenic protein levels detected in hippocampus by Western blot were not lower in the mutants compared to the WT transgenic version, yet the levels of all transgenic proteins were much lower than the levels of endogenous CSPα/DNAJC5 (Fig. 1C). Notably, the mutant proteins induced high molecular weight accumulations that were especially evident in the L115R mutant (Fig. 1C). The accumulations contained at least the GFP-fused mutant proteins; however, we do not know whether they co-accumulated with the endogenous CSPα /DNAJC5. This biochemical alteration, which was not observed for the WT transgenic protein, is consistent with the previously described increased tendency of the mutant forms of CSPα/DNAJC5 to accumulate (*26*, *27*, *29*, *31*, *32*). Because a major hallmark of lipofuscinosis is the presence of autofluorescence (*44*), we searched the hippocampus for AFSM and found a remarkable accumulation of punctate autofluorescent structures in the soma of CA3 pyramidal neurons in both mutants, being most pronounced in the L115R mutant (Fig. 2). Two aspects of this observation are remarkable: (i) We did not observe these structures in the NTC mice, and, in addition, the overexpression of the CSPα/DNAJC5-WT transgene did not generate those structures, indicating that the autofluorescence specifically occurred as a result of expression of CSPα/DNAJC5 mutant versions and not by the overexpression of normal CSPα /DNAJC5; and (ii) the apparently low levels of L115R-CSPα and L116Δ-CSPα proteins detected by immunofluorescence at the CA3 were, nevertheless, high enough to build up autofluorescence, reinforcing the notion that the pathogenic effect is rather precisely mediated by the mutations and not because of a nonspecific consequence of higher expression levels that, in any case, is much lower that the endogenous levels of CSPα /DNAJC5. However, we cannot rule out that the fusion to GFP might have a co-adjuvant effect enhancing the specific pathological effect of the mutations. In addition, we found that the autofluorescent structures were stained with antibodies against ATP5G (also known as mitochondrial ATP synthase subunit C), a protein typically used as lipofuscinosis marker (*62*, *63*). The electron microscopy analysis revealed the existence of GROD-like structures in the CA3 pyramidal cell soma of mutant mice with the same characteristics of GRODs previously described in brain samples of patients with Kufs disease/CLN4 (*6*, *13*). In NTC mice and in transgenic mice expressing the CSPα/DNAJC5 WT version, we observed different structures, which were less electron dense, sometimes with internal linear membranous arrays and often associated to a lipid vacuole likely corresponding to physiological age-dependent lipofuscin (Fig. 3). Lipid vacuoles were sometimes found in the L116Δ mutant, but not in the L115R mutant (Fig. 3). This is consistent with the human pathology of Kufs disease/CLN4 in which the typical clear component of adult lipofuscin is scanty (*6*, *8*). We could not detect the accumulation or the existence of GVBs (fig. S5), which are neuron-selective lysosomal structures associated with neurodegeneration and detected by lysosomal markers (*39*, *64*). The absence hereof suggests that the CSPα/DNAJC5 mutants do not cause the accumulation of lysosomal-related structures, although CSPα/DNAJC5 has been described to be in lysosomes and to be related to lysosomal function and dysfunction (*26*, *32*, *65*). In any case, the mutations might be interfering with lysosomal-mediated autophagy (*26*, *32*) and impairing the degradation of, for example, oxidized mitochondrial proteins that accumulate as autofluorescent stored material. On the other hand, several studies have reported that CSPα /DNAJC5 mediates the extracellular release of neurodegenerative-associated proteins (*21*–*24*), suggesting a general role for CSPα/DNAJC5 in misfolding-associated protein secretion (MAPS) (*66*). It has been proposed that the mutant versions of CSPα/DNAJC5 impair MAPS without interfering with microautophagy (*25*). According to that notion, the GROD-like structures that we have observed could be caused by a defect in MAPS, although further investigation is required to specifically test that notion in mice. We have detected moderate morphological changes in microglia, consistent with mildly increased microglia activation (figs. S10 and S11), as it has been also reported at the early stage of Kufs disease (*45*). Furthermore, we observed microglia engulfing lipofuscin and lipofuscin-containing neurons, which could reflect the physiological phagocytic activity of normal microglia on abnormal neurons as previously described (Figs. 4 to 6) (*54*, *55*). Our lipofuscinosis models in mice complement the existing *Drosophila* model (*32*). The models share key pathological features previously described in postmortem brain samples of human Kufs disease/CLN4 patients, such as the excessive formation of oligomers and the existence of electron dense structures detectable with electron microscopy (*6*, *8*, *27*, *29*, *31*, *61*), which, especially in mice, mirror the autofluorescence and the GRODs found in humans. In flies and mice, the L115R mutation has, in general, stronger effects than the L116Δ as was also previously observed in human brain samples and neuronal cultures (*27*, *28*, *31*, *61*). The phenotype in mutant flies is dose dependent (*32*). Flies expressing a single copy of the mutations showed higher levels of oligomers but did not suffer from neurodegeneration or any neurological phenotype which, in contrast, did appear upon the expression of two copies of the mutated gene (*32*). We cannot rule out that stronger phenotypes might also occur in mice upon increasing the mutant gene dosage. The stronger phenotype found in Thy1-GFP-CSPα-L115R mice could be also due to a gene dosage effect; however, we could not detect higher protein levels by Western blots (Fig. 1). In any case, our mice open unique opportunities to investigate the temporal cascade of molecular and cellular events that lead to neuronal lipofuscinosis and its relationship with other pathological alterations in vivo. An open question in the field is whether the Kufs disease/CLN4 phenotype is secondary to a loss of function of CSPα/DNAJC5 due to a dominant negative effect of the mutant forms that would be interfering with endogenous CSPα/DNAJC5 (*10*, *13*, *27*–*29*, *31*). We have investigated this notion studying several CSPα/DNAJC5 KO mouse lines. We concluded that the absence of CSPα/DNAJC5 by itself does not cause lipofuscinosis because we do not detect any sign of pathological lipofuscinosis using confocal and electron microscopy in several genetic mouse models such as the CSPα/DNAJC5 KO mice at 1 month of age and CSPα/DNAJC5 heterozygous KO mice at 8 months of age. However, those models might have limitations. On one hand, the conventional CSPα/DNAJC5 KO mice die very early when they are just 1 month old, so it could be argued that the mice are still too young to develop lipofuscinosis. On the other hand, although the heterozygous CSPα/DNAJC5 mice were studied during adulthood, it could be argued that the 35% reduction in CSPα/DNAJC5 is not enough to induce lipofuscinosis. However, we overcame those limitations using the CaMKIIα^CreERT2^:Ai27D:*Dnajc5*^flox^ mouse line in which CA3 pyramidal neurons, among other forebrain glutamatergic neurons, lack CSPα/DNAJC5 for up to 22 months without developing pathological lipofuscinosis. This observation strongly supports the notion that the mere elimination of CSPα/DNAJC5 in neurons is not enough to cause lipofuscinosis. The phenotype observed in *Drosophila* mutants is a hypermorphic gain-of-function phenotype that ameliorated upon a reduction in the endogenous levels of CSPα/DNAJC5 (*32*). To test that notion in mice, we examined lipofuscinosis in Thy1-GFP-CSPα-L115R transgenic mice in the homozygous and heterozygous CSPα/DNAJC5 KO background and found that lipofuscinosis persists even under a long-lasting (8 months) 35% reduction of endogenous CSPα/DNAJC5 levels, suggesting that the lipofuscinosis that we detected is not a consequence of gain of the normal function of the protein. We cannot rule out that hypermorphic phenotypes might appear in mice upon increasing the genetic dose of the mutant transgenes as it occurs in *Drosophila*. In addition, in our mice models, the promoter Thy1 drives the expression only in neurons but not in nonneuronal cells; however, because CSPα/DNAJC5 is also expressed at lower levels in microglia and astrocytes (*67*–*69*), it is conceivable that CLN4 human patients might suffer from glial dysfunction and stronger phenotypes might arise if both, neurons and glia, are dysfunctional (*70*). It has been proposed that, in CLN11, the neurological alterations arise only when different cell types, including neurons and glial cells, simultaneously lack progranulin (*71*–*73*). In that sense, the mice generated in the present study could perhaps develop a more severe phenotype if the mutant protein was additionally expressed in microglia and in case the expression in both neurons and microglia was necessary to unfold the full neurological phenotype*.* In any case, our findings indicate that the neuronal expression of CLN4 mutants is enough to cause neuronal lipofuscinosis that recapitulates human lipofuscinosis by a cell-autonomous mechanism in neurons. In addition, our transgenic models will be advantageous to dissect the specific contribution of neuronal and nonneuronal cell types, such as microglia, in the complex pathology of this disease.

In conclusion, we have developed novel mouse models to study the pathological neuronal lipofuscinosis observed in patients with Kufs disease/CLN4. Because using a variety of mouse models we reveal that the absence of CSPα/DNAJC5 cannot be stated as a primary cause of lipofuscinosis, we hypothesize that the mutant versions of CSPα/DNAJC5 might be interfering with key biochemical pathways such as proteostasis, likely caused by aberrant protein-protein interactions with, so far, unknown molecular partners. That would be a cell-autonomous mechanism occurring through the mutation-mediated gain of a novel, but pathological, function of CSPα/DNAJC5. The mice presented in this study open remarkable possibilities to further investigate the molecular mechanisms of Kufs disease/CLN4 and to test therapeutic strategies to revert pathological lipofuscinosis.

## MATERIALS AND METHODS

### Mice

All procedures involving animals were performed in accordance with the European Union Directive 2010/63/EU on the protection of animals used for scientific purposes and approved by the Committee of Animal Use for Research at the University of Seville. The animals were maintained in the university animal facilities (IBiS, CITIUS III-Oscar Pintado and Macarena campus) on a 12/12-hour light/dark cycle with unrestricted access to food and water.

### Generation of transgenic mice

GFP-CSPα fusion protein was excised from pEGFP-C1-CSPα with Sma I and Nhe I and cloned under control of Thy1 promoter into pThy1.2/pUC18 composed vector (*74*) in Hinc II (made blunt) and Nhe I. Mutant versions of CSPα/DNAJC5 cDNA (L115R and L116Δ) were produced by double polymerase chain reaction and cloned into Age I and Sac II sites of pThy1.2 vector. Primers used for cloning are described in table S4. For transgenic mice generation, expression cassette (8222 or 8219 base pairs) was excised with Not I and Pvu I sites, to remove bacterial sequences, purified from agarose gel and resuspended in 7.5 mM tris (pH7.4) and 0.2 mM EDTA. Pronuclear injections were carried out by “Centro de Producción y Experimentación Animal” at the University of Seville. Briefly, superovulated females of the mouse inbred strain Friend Virus B-type susceptibility NIH (FVB/N) were mated and after vaginal plug, pronuclear fertilized oocytes were extracted. DNA fragments were injected into male pronucleus and transferred into pseudopregnant FVB/N females. To identify the insertion sites of transgenes, genomic DNA was purified from the three transgenic lines (two mice per line) using the Monarch HWM DNA extraction kit for tissue (T3060L, New England Biolabs). Whole-genome sequencing was performed by the company Plasmidsaurus Inc. (Oregon, USA) using Oxford Nanopore technology. Genomic sequences are available at BioProject accession number PRJNA1174377. Sequences matching GFP were extracted from the whole-genome sequences and aligned with the mouse genome to determine the insertion sites. Conditional floxed mouse line *Dnajc5*^flox/flox^ was previously described (*65*). For Cre-recombinase induction, mice were fed a TMX-enriched diet (TAM400/CreER, TD.55125.I, Envigo) for 30 days to induce genetic removal of CSPα/DNAJC5. Mice were fed with TMX at 1 month of age, except the mice that were used for electron microscopy 8 months after TMX that were fed with TMX when they were 2 months old. Genotyping protocols are detailed in table S5.

### Immunohistochemistry and immunofluorescence

For brain fixation and collection, mice were anesthetized with 2% 2,2,2-tribromoethanol (T48402, Sigma-Aldrich). Mice were perfused with 1× phosphate-buffered saline (PBS) [137 mM NaCl, 2.7 mM KCl, 10 mM Na_2_HPO_4_, and 2 mM KH_2_PO_4_ (pH 7.4)] to eliminate blood from tissues and then with 4% paraformaldehyde (PFA) in PBS to fix tissues. Collected brains were postfixed in 4% PFA for 24 hours at 4°C and then cryoprotected in 30% sucrose in 1× PBS with 0.01% sodium azide. A Leica CM 1950 cryostat was used for serial sectioning of either sagittal or coronal planes of 40 μm thickness. Slices were stored at −20°C in 50% glycerol in 1× PBS until use. For those antibodies that need antigen-retrieval to recognize their targets, free-floating brain slices were incubated in 10 mM sodium citrate (pH 6) in PBS during 30 min at 80°C and then blocked with 2% nonfat milk and 0.3% Triton X-100 in PBS for 30 min. Then, slices were incubated in blocking solution with 3% fetal bovine serum and 0.3% Triton X-100 in PBS for 1 hour at room temperature. Incubation with primary antibodies (see table S2) was performed overnight at 4°C in the same blocking solution. Secondary antibodies were incubated in the same blocking solution in dark for 2 hours at room temperature (see table S3). Slices were then washed, mounted, and coverslipped with FluorSave Mounting Medium (Merck Millipore, no. 345789). In the case of immunohistochemistry, endogenous peroxidase was inactivated for 10 min in 8% methanol and 2.5% H_2_O_2_ in PBS. Biotinylated secondary antibody was used, and an Avidin-Biotin Complex (ABC) kit was used for amplification of signal (Vectastain Elite ABC Kit; Vector Laboratories, no. PK-6100). Peroxidase activity was revealed by 0.02% diaminobenzidine with 0.01% H_2_O_2_ and enhanced with 0.04% NiCl_2_ in phosphate buffer [10 mM Na_2_HPO_4_ and 2 mM KH_2_PO_4_ (pH 7.4)]. Reaction was stopped by washing in phosphate buffer. Last, sections were placed in gelatinized glass slides and coverslipped with Distyrene-Plasticizer-Xylene (DPX) mounting medium (Sigma-Aldrich). In the case of immunohistochemistry for GVB markers, endogenous peroxidase activity was quenched in 0.3% H_2_O_2_ in PBS for 12.5 min. Sections were incubated with blocking solution containing 5% normal goat serum (Gibco), 2.5% bovine serum albumin (BSA; Thermo Fisher Scientific), and 0.2% Triton X-100 (Thermo Fisher Scientific) in PBS for 1 hour at room temperature. Incubation with primary antibodies (see table S2) was performed overnight at 4°C in blocking solution. Sections were incubated with biotinylated secondary antibody (see table S2) diluted in blocking solution for 90 min at room temperature, followed by incubation with Vectastain ABC kit components (Vector Laboratories) (1:800 in blocking solution) for 90 min at room temperature. Each of these steps was followed by extensive washing in PBS (three times 10 min). Signal was developed using the chromogen DAB (DAB Substrate Kit, Vector Laboratories), and sections were washed in H_2_O (two times quickly, two times for 5 min). Sections were counterstained with hematoxylin (diluted 1:1 in Milli-Q water) for 30 s, extensively washed with Milli-Q water, and destained in acidic ethanol (HCl 1:100 diluted in 70% ethanol) for a few seconds. Sections were mounted on gelatin-coated microscope slides, dehydrated, and mounted using Quick-D mounting medium (Klinipath BV). Stained sections were imaged using a Leica DM2500 microscope with Leica MC170 HD camera. For microglia, astrocytes, neurons, and myelin immunostaining analysis, free-floating brain slices were first rehydrated and then incubated for 2 hours in PBS containing 0.3% Triton X-100, 10% normal donkey serum, and 1% BSA, followed by incubation with primary antibodies (see table S2) overnight at 4°C. After three washes in PBS, sections were incubated with secondary antibodies for 2 hours at room temperature.

### Acquisition and analysis of confocal images

Confocal images were acquired in a Nikon A1R+ confocal, a Leica TCS SP2 AOBS, or a Leica Stellaris 8 microscope using 40× and glycerol immersion 20×/63× objectives. Maximal *Z*-projection was done choosing the same number of planes for each staining. Quantification was done with the help of custom macros for Fiji (ImageJ) (available at https://github.com/SLopezBegines/ImageJ-Macros) to quantify size, density, and intensity of lipofuscin granules in CA3 hippocampus region. In brief, by using Analyze Particles tool, a specific region of interest (ROI) was done for every particle above of a threshold applied to a selected channel (usually 561-nm laser line channel) and with a size above of 10 μm^2^. Distribution of ROIs was saved (white panels), and the size, density of accumulations in CA3 area, and intensity of signal in each ROI in channel were quantified. Pearson’s *r* index for colocalization analysis was obtained using Coloc2 3.0.5 plugin from ImageJ. 3D reconstruction was performed using Bitplane Imaris ×64 9.6.0 image analysis software (Oxford Instruments, Concord, MA). Images were first subjected to background subtraction and then processed with the surface and filament module to rebuild microglia, cell bodies, microglia processes, lipofuscin particles, and neurons.

### Transmission electron microscopy

Brains were collected after anesthesia and sectioned at 200 μm thickness using vibratome (Leica VT1200S) immersed in ice cutting solution (222 mM sucrose, 11 mM glucose, 3 mM KCl, 1 mM NaH_2_PO_4_, 26 mM NaHCO_3_, 7 mM MgCl_2_, and 0.5 mM CaCl_2_ in ddH_2_O). Sections were then fixed in a 0.1 M sodium cacodylate (pH 7.4) and 2.5% glutaraldehyde solution for 2 hours at room temperature, followed by washing twice with 0.1 M cacodylate for 10 min. Sections were kept in 0.1 M cacodylate with 0.01% sodium azide at 4°C until use. For Spurr resin inclusion, samples were fixed in 1% OsO4, stained with 2% uranyl acetate, dehydrated in a gradient up to 100% acetone, and infiltrated in a gradient of acetone to Spurr resin. Ultrathin 70-nm sections were observed in a Zeiss Libra 120 transmission electron microscope. For quantification, the number of lipofuscin accumulations was quantified in each 2*kx* field, and the average number of accumulations per image and per mouse was used. Numbers describe the number of images and number of mice used.

### Immunoblotting and quantification

Fresh tissue was flash frozen in liquid nitrogen and kept at −80°C until use. Hippocampal samples were homogenized in homogenization buffer [50 mM tris-HCl (pH 7.4), 150 mM NaCl, 1% SDS, 5 mM EDTA, 1 mM β-glycerophosphate, 1 mM NaF, 1 mM phenylmethylsulfonyl fluoride, and one cOmplete protease inhibitor cocktail tablet; Merck Millipore, no. 11697498001) with blue pestles and then under rotation for 2 hours in the cold-room, followed by centrifugation at 16,000*g* for 30 min at 4°C. Protein content of soluble fractions was quantified using the Pierce BCA Protein Assay Kit (Thermo Fisher Scientific, 23227), following the manufacturer’s instructions, and normalized. Samples were boiled in Laemmli 1× [50 mM tris-HCl (pH 6.8), 10% glycerol, 2% SDS, 0.0067% bromophenol blue, and 0.1 M α-mercaptoethanol] and boiled at 95°C for 5 min. Samples were resolved in standard 12% tris-glycine polyacrylamide gels and transferred to polyvinylidene difluoride membranes. Protein signal was detected using enhanced chemiluminescence (Clarity Western ECL Substrate; Bio-Rad, 170-5060), and images were taken using the Chemidoc Touch Imaging System (Bio-Rad) and analyzed with Fiji (ImageJ). Data were analyzed using Microsoft Excel and GraphPad Prism 8.

### Cathepsin D enzymatic assay

Cathepsin D activity was measured in cell lysates using a fluorometric cathepsin D activity assay kit (Abcam, ab65302) according to manufacturer’s instructions. Briefly, whole hippocampi from 16 mice (4 for each condition) were snap frozen in liquid nitrogen and stored at −80°C until its processing. Samples were weighted and lysed by pestle homogenization sitting on ice in 20 μl per mg of protein of lysis buffer provided with the kit. Fluorescence was measured in duplicates 2 hours after substrate addition on a CLARIOstar Microplate Reader (BMG LabTech) at an excitation of 328 nm and emission of 460 nm. Cathepsin D activity was expressed by relative fluorescence units per milligram of protein. Protein concentration was determined by bicinchoninic acid assay. To obtain specific Cathepsin D enzyme activity, total Cathepsin D protein (Abcam, ab75852) levels were determined by immunoblotting and normalized to β-actin (Sigma-Aldrich, a2228) as a loading control.

### Behavioral analysis

The open-field test was conducted to evaluate locomotor activity in mice. The setting consisted of a square arena (45 cm by 45cm) with high, opaque walls to prevent escape and minimize external distractions. The floor included a central squared zone (40% of the total area), with the remaining space considered the peripheral zone. Before the test, mice were acclimated to the testing room for at least 30 min to reduce stress induced by environmental changes. Mice were individually placed in a corner of the arena and allowed to explore freely for a 15-min period. Activity was monitored by video tracking with SMART 3.0 software (Panlab). Data analysis reported detailed information about distance, speed, time, and location within the central and peripheral arena. Scripts for analysis are available at https://github.com/SLopezBegines/R_code. The rotarod test was used to assess motor coordination, balance, and motor learning. One day before testing, each mouse underwent a training session consisting of three trials on the rotarod (LE 8200, Panlab) at a constant speed (5 rpm) for a duration of 3 min per trial with resting intervals of 15 min. This session was conducted to familiarize the animals with the apparatus and reduce novelty effects during the actual test. The following day, mice were placed individually on the rotarod, with a rotation speed gradually increased in a stepwise manner (1 rpm every 5 s from 5 to 40 rpm for 3 min) until the mouse fell off the rod or clung to it. Each mouse underwent three trials, with a minimum rest period of 15 min between trials to avoid fatigue. The average latency to fall was calculated for each mouse across the three trials. Survival curves were based on the age of death of mice that were recorded as having died of natural causes in the mouse colony database.

### Statistical analysis

Details provided in the text and/or figure legends for all the experiments provided enough detail to enable a knowledgeable reader with access to the original data to verify the results. The values for *n*, *P*, and the specific statistical test performed for each experiment are included in the appropriate figure legend or in the main text.
