# Supplementary material for: Neuronal lipofuscinosis caused by Kufs disease/CLN4 DNAJC5 mutations but not by a CSPα/DNAJC5 deficiency
Source: Sci Adv. 2025 May 21;11(21):eads3393. doi: 10.1126/sciadv.ads3393 (PMC12094229; doi:10.1126/sciadv.ads3393)
Supplement: Supplementary file 1 — Figs. S1 to S25 Tables S2 to S5 Legend for table S1 Legends for movies S1 and S2 [file sciadv.ads3393_sm.pdf]

Science Advances
AAAS

Supplementary Materials for

Neuronal lipofuscinosis caused by Kufs disease/CLN4 DNAJC5 mutations but not by a CSPα/DNAJC5 deficiency

Santiago López-Begines *et al.*

Corresponding author: Rafael Fernández-Chacón, rfchacon@us.es

Sci. Adv. **11**, eads3393 (2025)
DOI: 10.1126/sciadv.ads3393

The PDF file includes:

Figs. S1 to S25
Tables S2 to S5
Legend for table S1
Legends for movies S1 and S2

Other Supplementary Material for this manuscript includes the following:

Table S1
Movies S1 and S2

SUPPLEMENTARY FIGURES

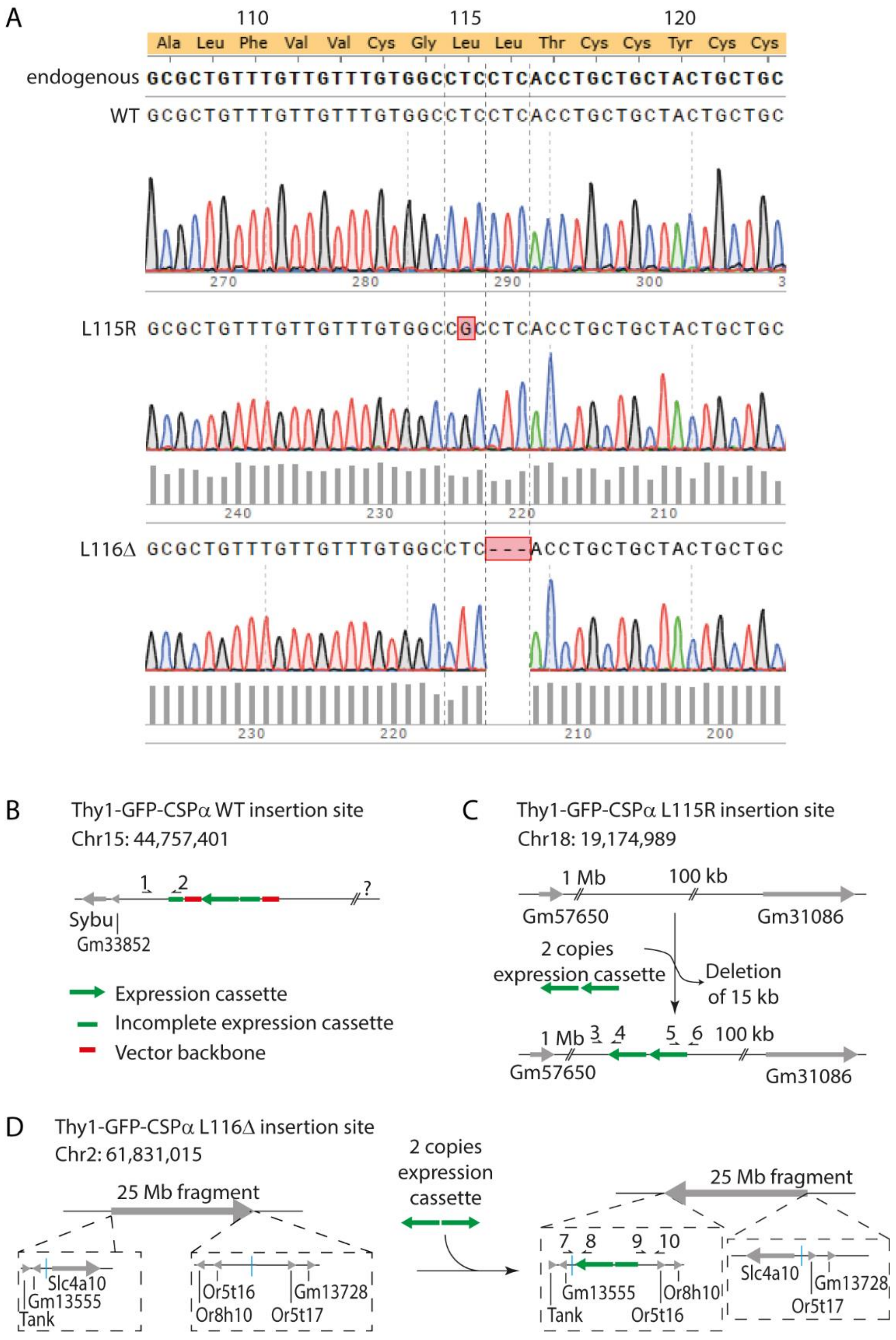

Fig. S1. Characterization of transgene insertion sites in the Thy1-GFP-CSPα-WT, Thy1-GFP-CSPα-L115R and Thy1-GFP-CSPα-L116Δ mouse lines. A. Electropherograms and sequence alignments reveal the specific point mutations (L115R and L116Δ) in the corresponding transgenes. **B, C** and **D.** Schematic representations of the genomic regions surrounding the transgene insertion sites. The maps depict the integration of the transgene into the mouse genome, with the full expression cassette (green arrow), incomplete expression cassette (green segment) and vector backbone (red segment). Chromosomal positions of the transgenes insertion within the mouse genome are indicated. Consensus sequencing identified one full copy and two incomplete copies of Thy1-GFP-CSPα-WT inserted in tandem and in antisense orientation into chromosome 15 (position 44,757,401), 57 kb downstream of the gene *Gm33852*. The exact 5' end insertion site remains however undetermined. For Thy1-GFP-CSPα-L115R, the insertion replaced 15 kb of genomic DNA with two tandem copies in the same orientation into chromosome 18 (position 19,174,989), 110 kb upstream of the gene Gm31086. In the case of Thy1-GFP-CSPα-L116Δ, the transgene caused a 25 Mb inversion on chromosome 2 (position 61,831,015) without disrupting any known genes. The inversion occurred between the *Or5t16* and *Scl4a10* genes, with one complete copy and one incomplete copy of the transgene inserted in tandem, 3 kb from the *Or5t16* gene and 30 kb from the *Gm13555* gene. PCR and sequencing of the PCR products confirmed the insertion sites, ensuring accurate mapping of the transgene integration within the mouse genome. The position of the primers used is indicated by small half arrows numbered from 1 to 10.

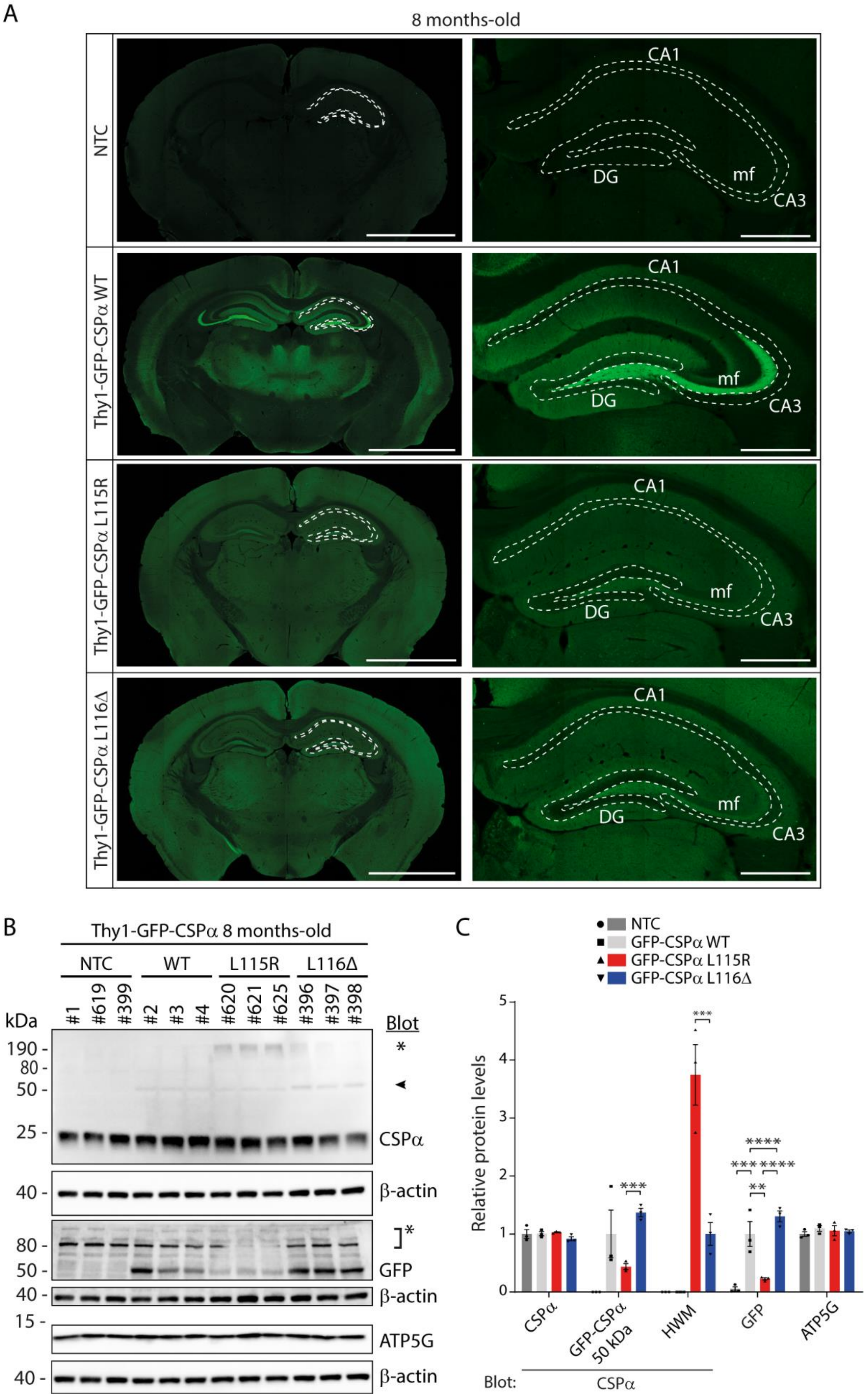
A
8 months-old
NTC
Thy1-GFP-CSPα WT
Thy1-GFP-CSPα L115R
Thy1-GFP-CSPα L116Δ
CA1
DG
mf
CA3
B
Thy1-GFP-CSPα 8 months-old
NTC
WT
L115R
L116Δ
#1 #619 #399 #2 #3 #4 #620 #621 #625 #396 #397 #398
kDa
Blot
190
80
50
25
40
15
CSPα
β-actin
GFP
ATP5G
C
NTC
GFP-CSPα WT
GFP-CSPα L115R
GFP-CSPα L116Δ
Relative protein levels
CSPα
GFP-CSPα 50 kDa
HWM
GFP
ATP5G
Blot:
CSPα

Fig. S2. Transgenic expression of wild-type and CLN4 mutant forms of CSPα/DNAJC5 in mouse brain at 8 months of age. A. Representative epifluorescence images of GFP immuno-staining demonstrate widely distributed expression of all transgenes in brain in 8 months old mice. General stronger transgene expression of GFP-CSPα WT especially evident at hippocampal mossy fibers. No signal is detected in non-transgenic control (NTC) mice. Scale bar: left, 2.5 mm; right, 500 µm. **B.** Transgenic proteins detected by western blot of hippocampal extracts from 8 months old mice. Numbers indicate mouse ID number. Upper blot, endogenous CSPα/DNAJC5 is detected in all samples while a band corresponding to GFP-tagged CSPα/DNAJC5 (arrowhead, 50 kDa) appears in transgenic samples but not in NTC. High molecular weight species (asterisk *) are detected in mutant transgenic samples, especially in the L115R mutant. GFP signal is only detected in transgenic samples. Non-specific band due to GFP antibody (asterisk *). β-actin used as loading control**. C.** Levels of selected hippocampal proteins. Relative protein levels normalized to non-transgenic mouse lines, except for GFP quantification that was normalized to GFP levels of the GFP-CSPα-WT transgenic line. Data are presented as mean ± SEM. Quantitative data available at Supplementary Table 1. Two-way ANOVA with Tukey's post hoc test (*P < 0.05; **P < 0.01; ***P < 0.001; ****P < 0.0001).

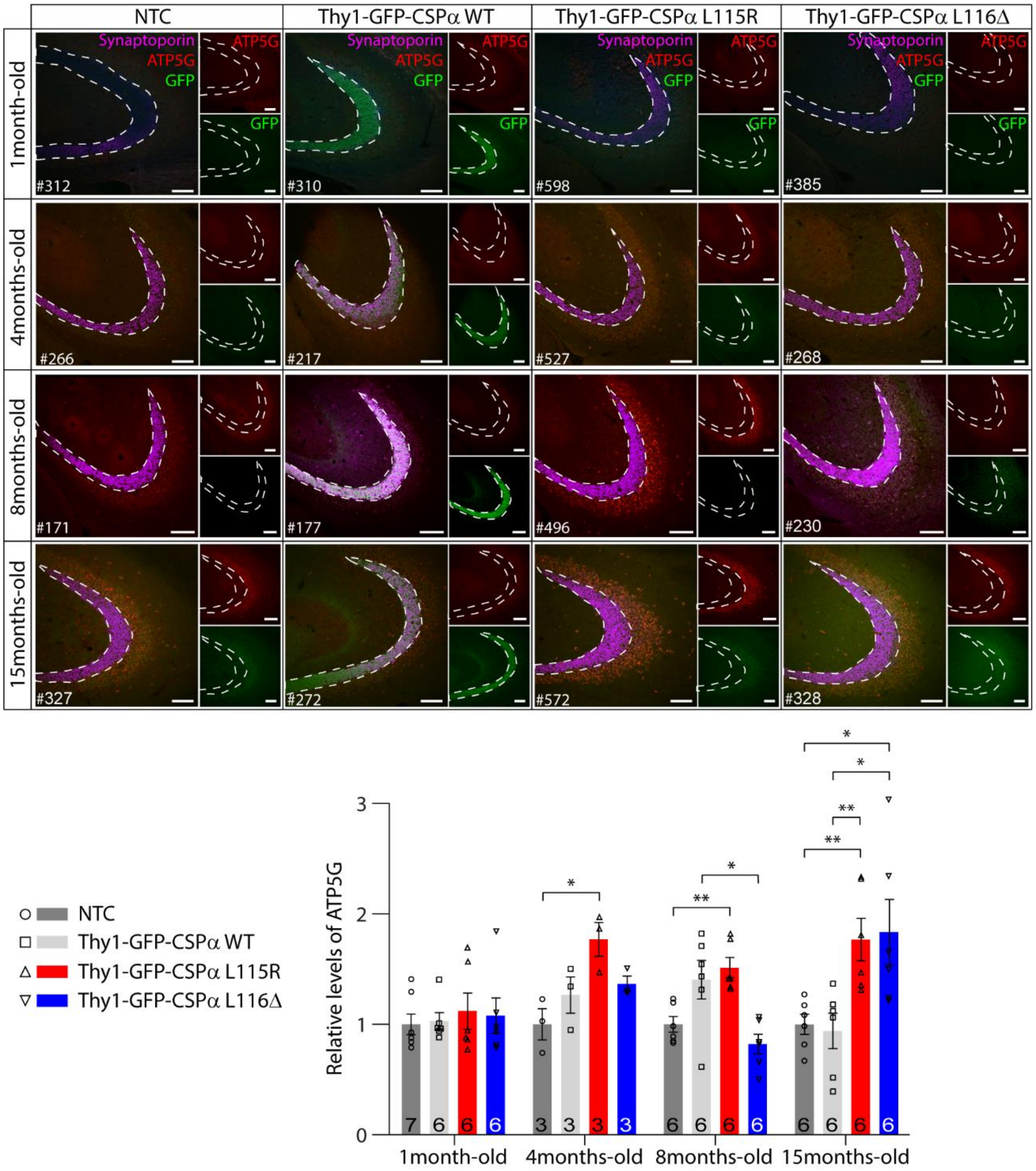

Fig. S3. Preferential detection of ATP5G signal at the CA3 region starts at 4 months of age in CLN4 mutant mice. Immunolabeling of pyramidal neurons at the CA3 hippocampal region with antibodies against ATP5G (red), GFP (green) and presynaptic mossy fiber with an antibody against synaptoporin (magenta) across four different genotypes (non-transgenic control (NTC), GFP-CSPα-WT, GFP-CSPα-L115R and GFP-CSPα-L116Δ) at different ages (1, 4, 8 and 15 months of age). Mouse identification numbers are shown in each image. Data are presented as mean ± SEM. Numbers at the base of the graph bars indicate the total number of images analyzed (from 3 mice per genotype). Scale bar 100 µm. Unpaired t-test *$P < 0.05$; **$P < 0.01$. Quantitative data available in Supplementary Table 1.

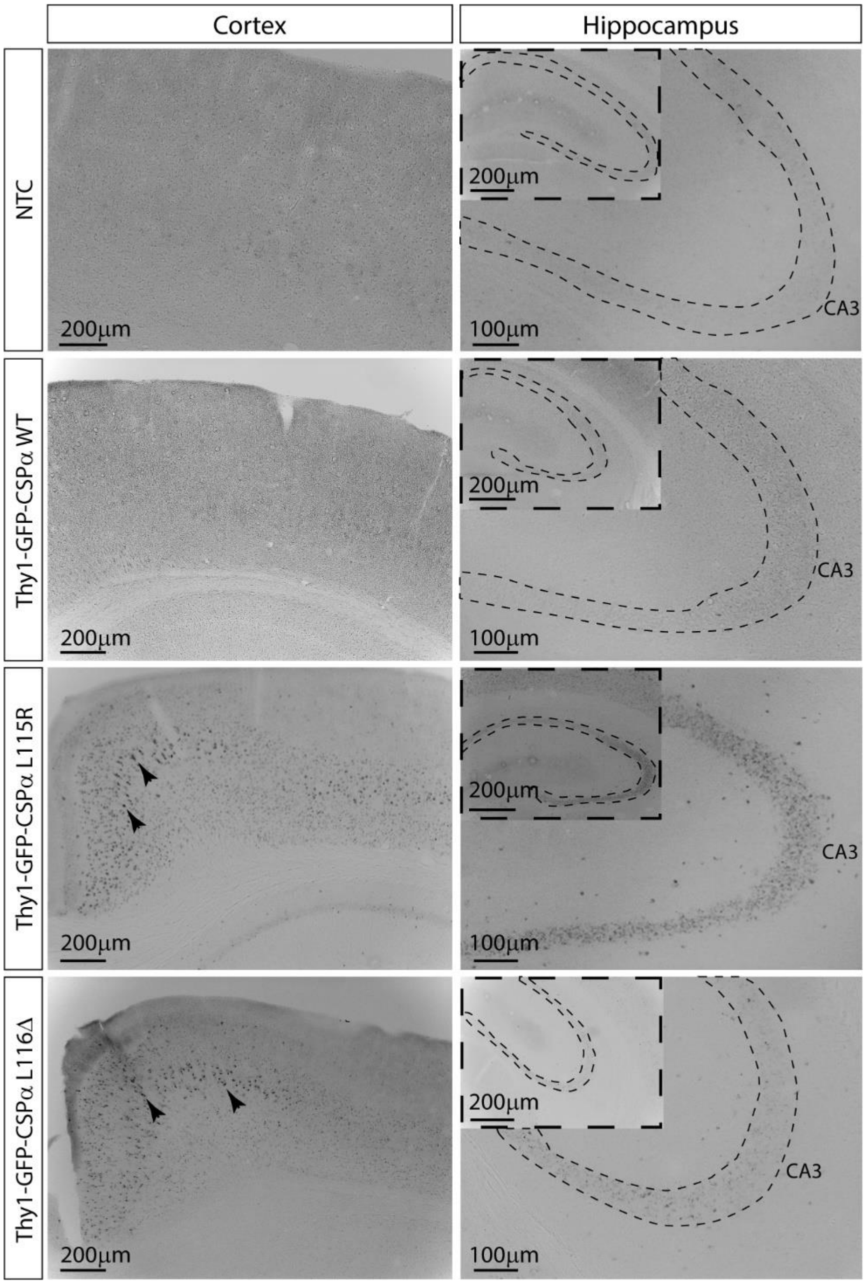

Fig. S4. Pathological lipofuscinosis visualized with bright field microscopy. Immunohistochemical labeling based on peroxidase-staining demonstrates the pathological accumulation of ATP5G at the CA3 region of the hippocampus and in the cortex of Thy1-GFP-CSPα-L115R and Thy1-GFP-CSPα-L116Δ mice, but not in control animals.

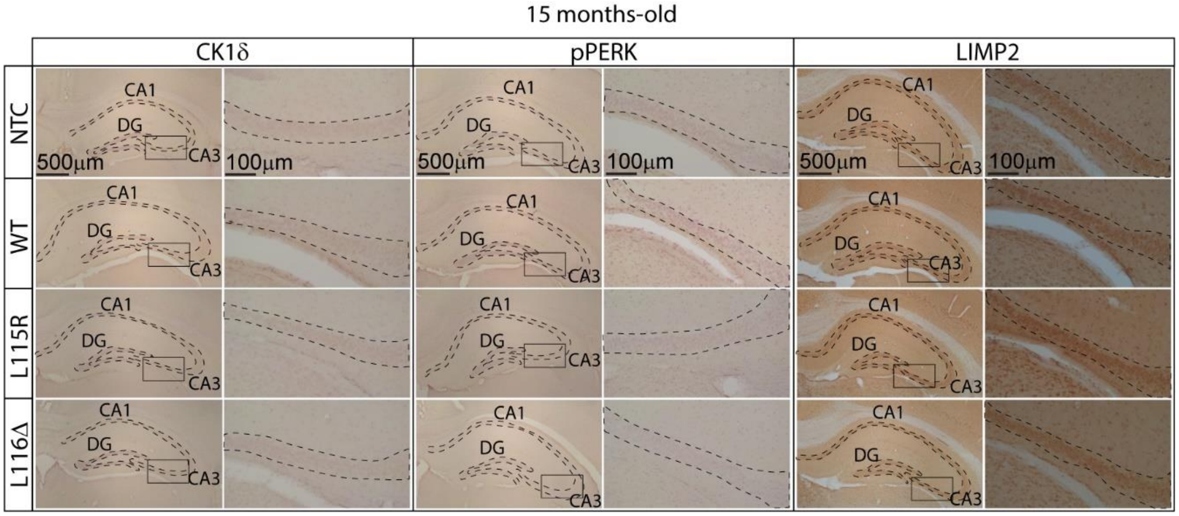

Fig. S5. CLN4-associated CSPα/DNAJC5 mutations do not lead to hippocampal granulovacuolar degeneration body (GVB) formation. Immunohistochemical labeling with antibodies against CK1δ, pPERK and LIMP2 of hippocampal sections of non-transgenic control (NTC), Thy1-GFP-CSPα-WT (WT), Thy1-GFP-CSPα-L115R (L115R) and Thy1-GFP-CSPα-L116Δ (L116Δ) in 15 months-old mice. No GVBs are found in mice from any of the genotypes, as shown by immunolabeling of CK1δ and pPERK antibodies that are used to detect GVBs in mice with tau pathology. No clear difference among the different genotypes is detected by immunolabeling with the lysosomal and GVB membrane marker LIMP2 . Nuclei are counterstained with hematoxylin in all images.

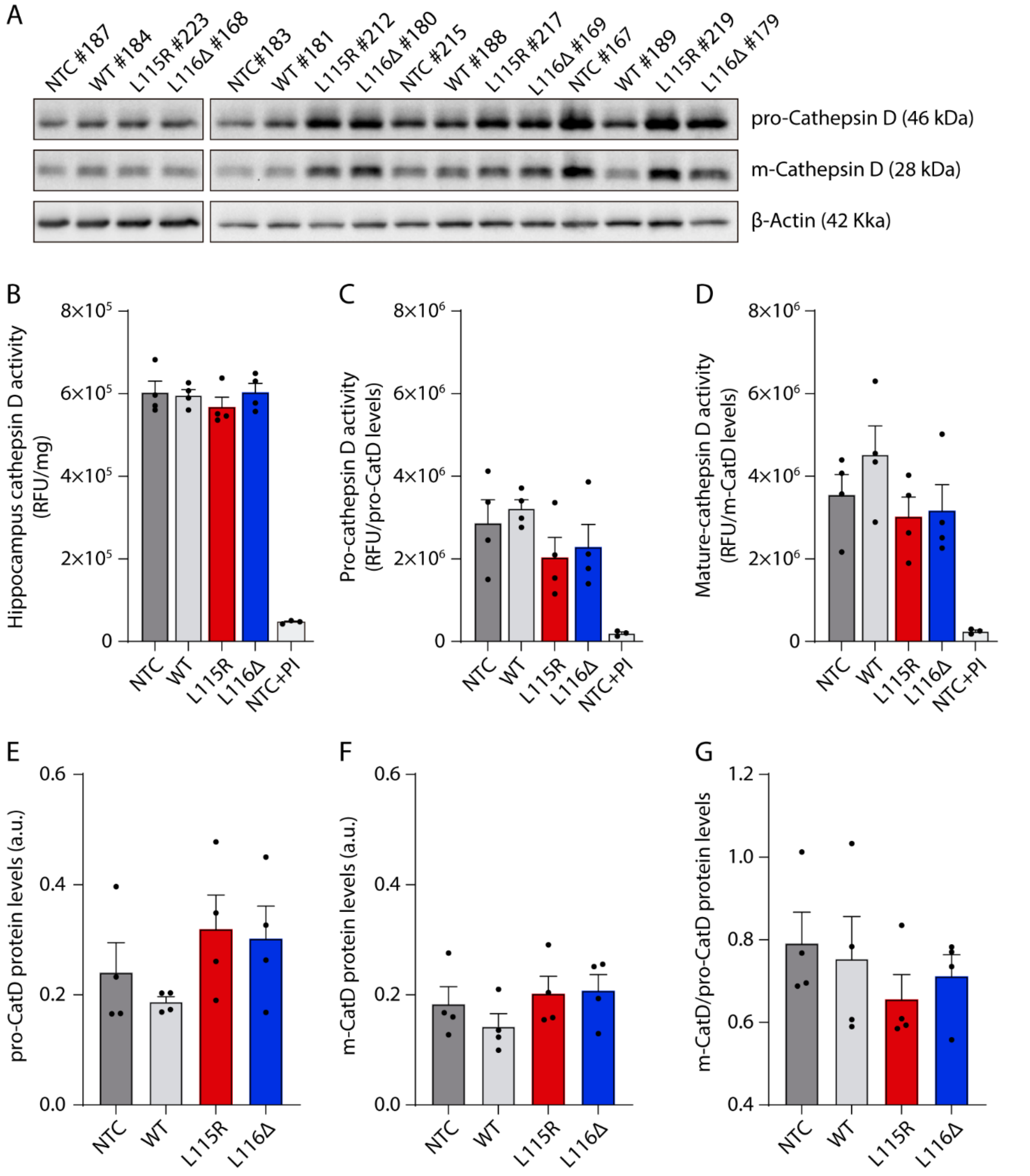

Figure S6. Cathepsin D activity and protein levels are normal in Thy1-GFP-CSPα-L115R and Thy1-GFP-CSPα-L116Δ transgenic mice. A. Western blot analysis of pro-cathepsin D and mature cathepsin D (m-cathepsin D) protein levels from hippocampal extracts of 14-16 months old mice analyzed in non-transgenic control (NTC), Thy1-GFP-CSPα-WT (WT), Thy1-GFP-CSPα-L115R (L115R) and Thy1-GFP-CSPα-L116Δ (L116Δ). β-actin used as loading control. **B.** Fluorometric assay for cathepsin D activity in the same hippocampal extracts. Relative fluorescence unit (RFU) per mg of protein calculated by normalizing the fluorescence signal to the amount of protein present in each sample. **C.**

Enzymatic activity data from panel B normalized to pro-cathepsin D protein levels. **D.** Enzymatic activity data from panel B normalized to m-cathepsin D protein levels. **E.** Pro-cathepsin D protein quantification normalized to β-actin protein levels. **F.** M-cathepsin D protein quantification normalized to β-actin protein levels. **G.** Ratio of m-cathepsin D/pro-cathepsin D protein levels. Data are presented as mean ± SEM from 4 different animals per group. As negative control for the cathepsin D activity, protease inhibitors (PI) were added to the NTC sample in **B**, **C** and **D**. Statistical analysis performed by unpaired t-test comparing each condition with the NTC group. No statistically significant differences were found ($P > 0.05$ for all comparisons).

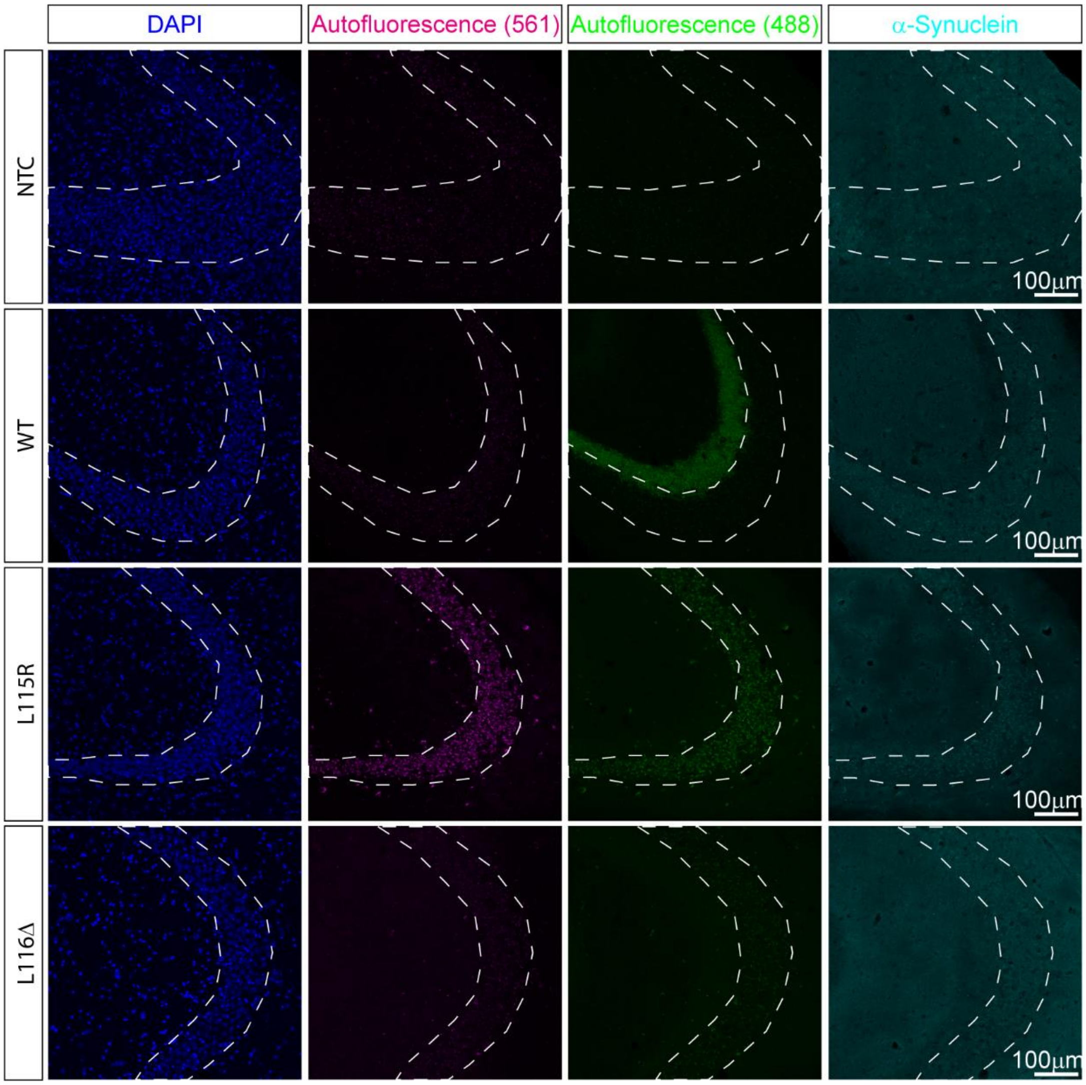

Figure S7. Normal intensity and distribution of α-synuclein at the CA3 hippocampal region of Thy1-GFP-CSPα-L115R and Thy1-GFP-CSPα-L116Δ transgenic mice. Representative merged confocal images of mouse hippocampal slices from non-transgenic control (NTC), GFP-CSPα-WT (WT), GFP-CSPα-L115R (L115R) and GFP-CSPα-L116Δ (L116Δ) in 12-15 months-old mice showing DAPI (blue), autofluorescence in 561 nm laser line (magenta), autofluorescence in 488 nm laser line (green) and immunolabeling of alpha-synuclein (cyan). Scale bar 100 µm. No significant changes observed in alpha-synuclein staining.

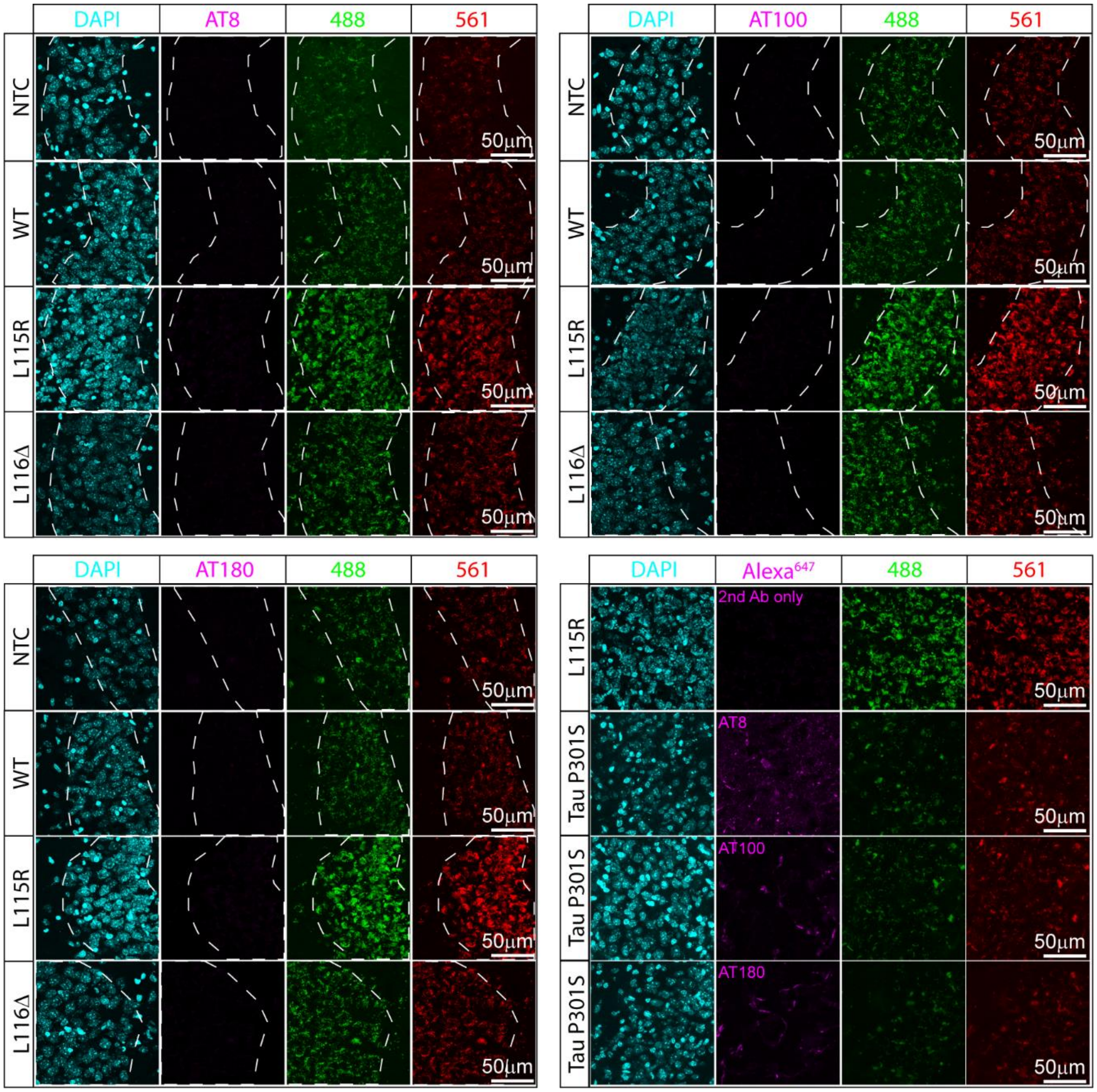

Figure S8. Absence of hyperphosphorylated tau in Thy1-GFP-CSPα-L115R and Thy1-GFP-CSPα-L116Δ transgenic mice. Representative confocal images of mouse hippocampal slices from non-transgenic control (NTC), Thy1-GFP-CSPα-WT (WT), Thy1-GFP-CSPα-L115R (L115R) and Thy1-GFP-CSPα-L116Δ (L116Δ) transgenic 12-15 months-old mice stained with DAPI (cyan), phospho-tau (antibodies AT8, AT100 and AT180, magenta), autofluorescence in 488 nm laser line (green) and autofluorescence in 561 nm laser line (red). Antibodies against different forms of phospho-tau reveal a clear signal in the brain of Tau P301S mutant mice used as positive control, however, there is not any detectable signal in CLN4 mutants and control mice Scale bar 50 μm.

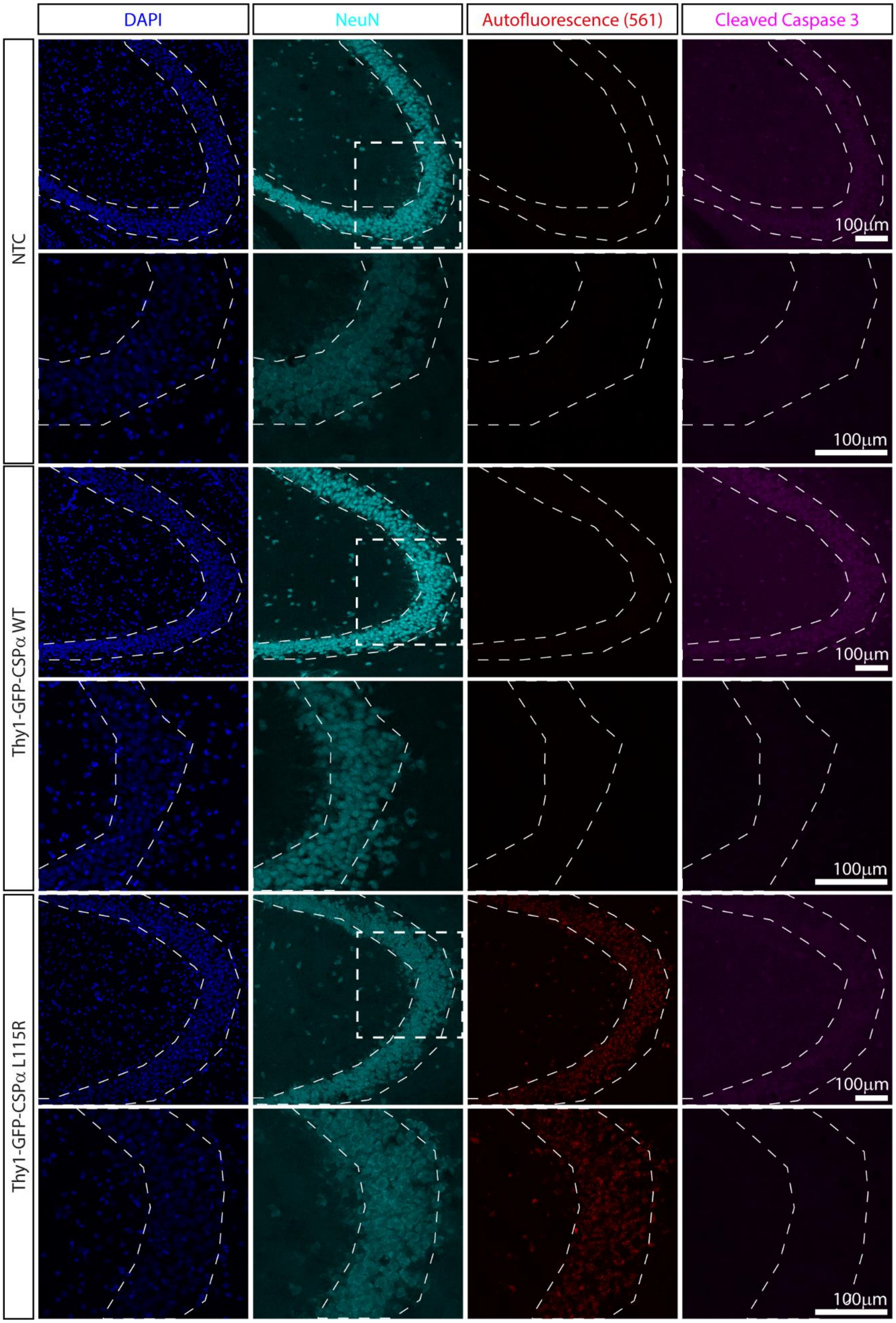
DAPI
NeuN
Autofluorescence (561)
Cleaved Caspase 3
NTC
Thy1-GFP-CSPα WT
Thy1-GFP-CSPα L115R
100µm
100µm
100µm
100µm
100µm
100µm

Figure S9. Lack of cell death signal in Thy1-GFP-CSPα-L115R transgenic mice. Representative merged confocal images of mouse hippocampal slices from non-transgenic control (NTC), GFP-CSPα-WT and GFP-CSPα-L115R 12-15 months old transgenic mice stained with DAPI (blue), NeuN marker (cyan), autofluorescence in the 561 nm laser line (red), and cleaved caspase-3 (magenta). Lipofuscinosis is evident only Thy1-GFP-CSPα-L115R, however, cleaved caspase-3 signal is absent consistent with a lack of cell death associated to lipofuscinosis.

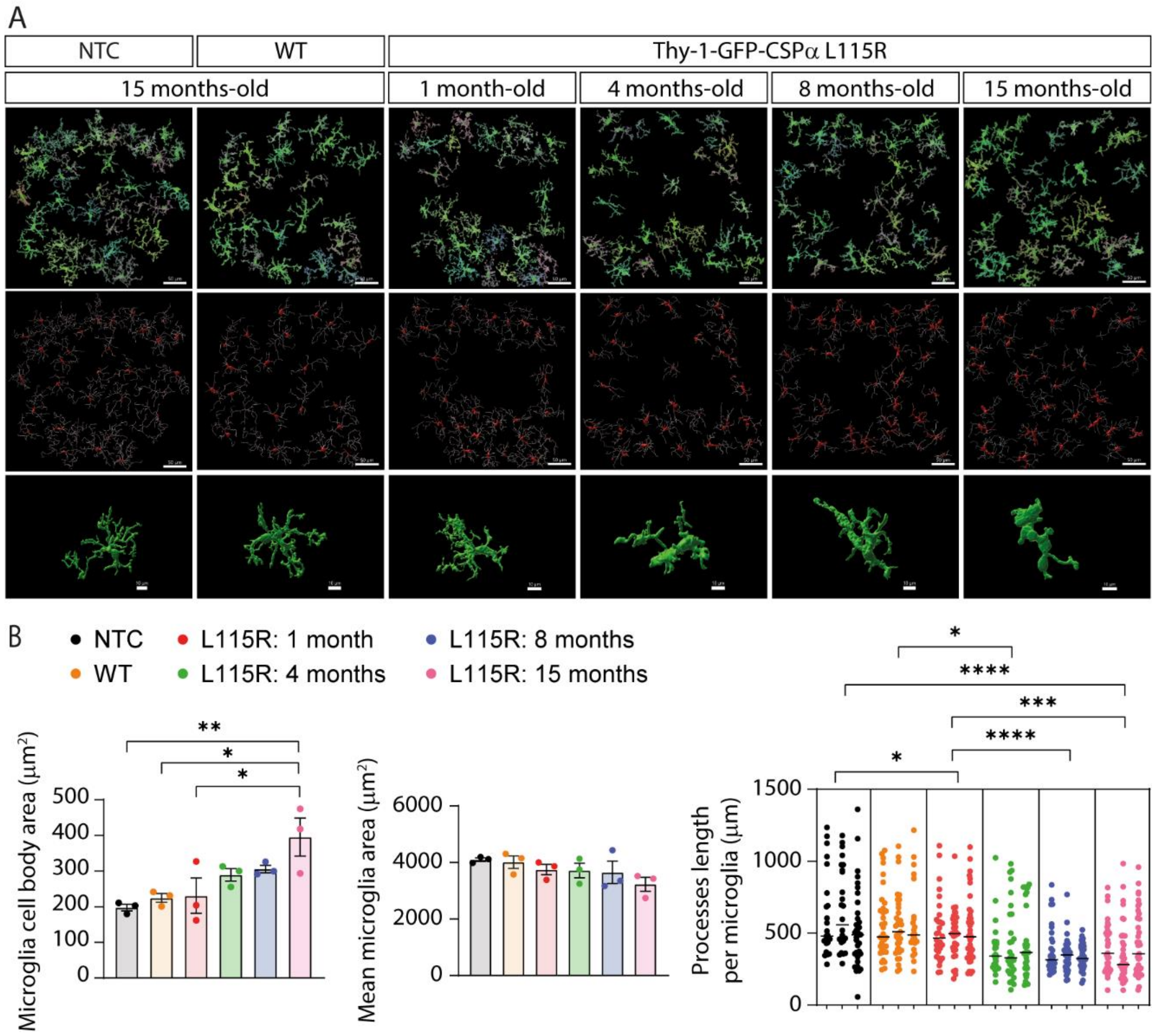

Figure S10. Morphological analysis of microglia in Thy1-GFP CSPα L115R transgenic mice. A. Representative images of immunostaining of the CA3 region microglia (Iba-1, green) in control (NTC), GFP-CSPα-WT and GFP-CSPα-L115R in 1, 4, 8 and 15 month-old mice together with IMARIS 3D Reconstruction for the analyzed cells. Scale bar 50 µm and 10 µm for the third row. **B.** Quantification of cell body, mean area and mean processes length of microglia in NTC, GFP-CSPα-WT and GFP-CSPα-L115R in 1, 4, 8 and 15 month-old mice were represented. Data were presented as mean ± SEM, Two-way ANOVA with Tukey's post hoc test (*P < 0.05, **P < 0.01, ****P < *0.0001)*, N = 3 mice/group, 3 images/mouse. At least 25 microglia cell were analyzed to get the processes length of microglia data.

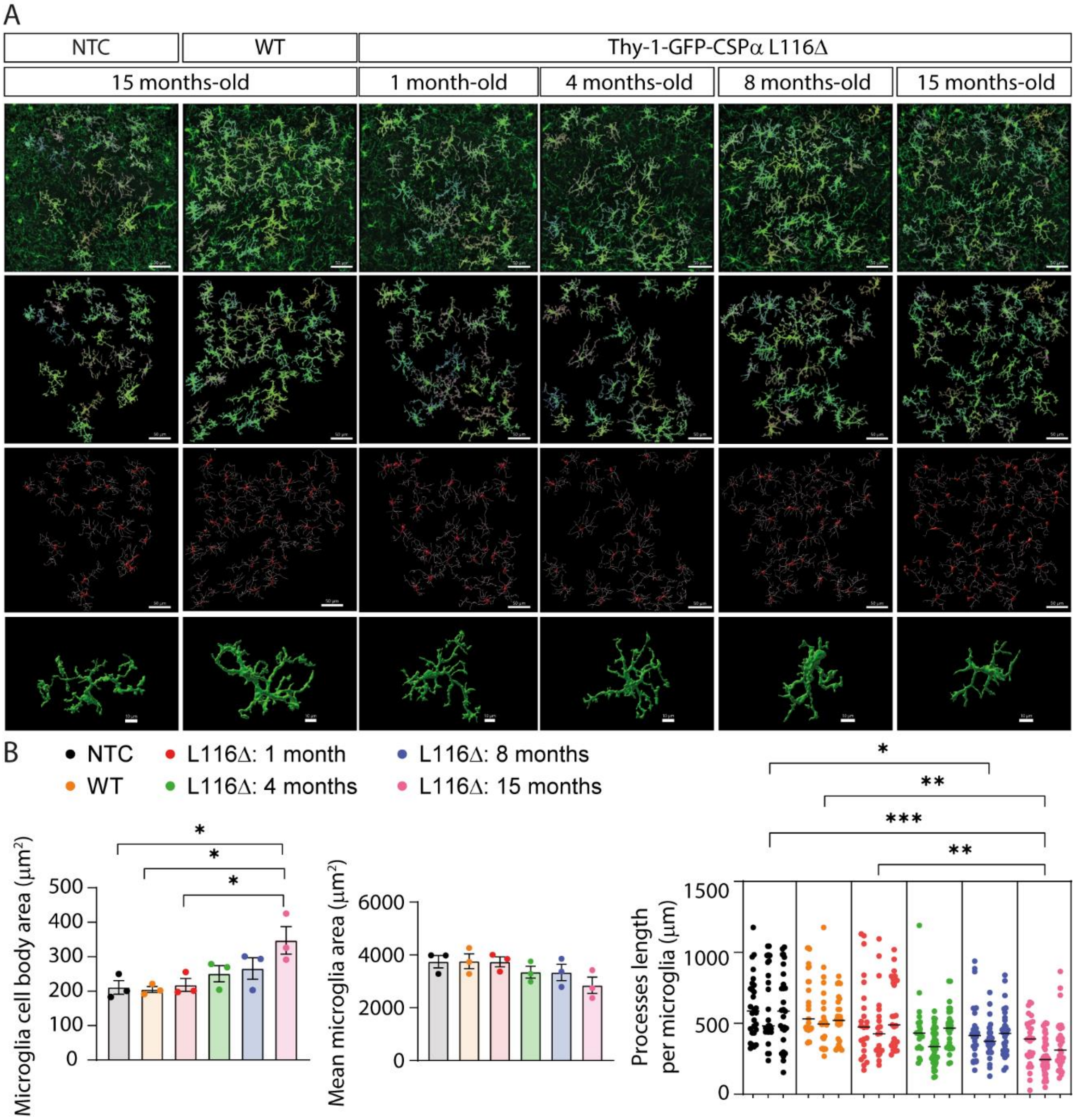

Figure S11. Morphological analysis of microglia in Thy1-GFP CSPα L116Δ transgenic mice. A. Representative confocal images of immunostaining of the CA3 region microglia (Iba-1, green) in control (NTC), GFP-CSPα-WT and GFP-CSPα- L116Δ in 1, 4, 8 and 15 month-old mice together with IMARIS 3D Reconstruction for the analyzed cells. Scale bar 50 µm and 10 µm for the fourth row. **B.** Quantification of cell body, mean area and mean processes length of microglia in NTC, GFP-CSPα-WT and GFP-CSPα- L116Δ in 1, 4, 8 and 15 month-old mice were represented. Data were presented as mean ± SEM, Two-way ANOVA with Tukey's post hoc test (*P < 0.05; **P < 0.01), N = 3 mice/group, 3 images/mouse. At least 20 microglia cell were analyzed to get the processes length of microglia data.

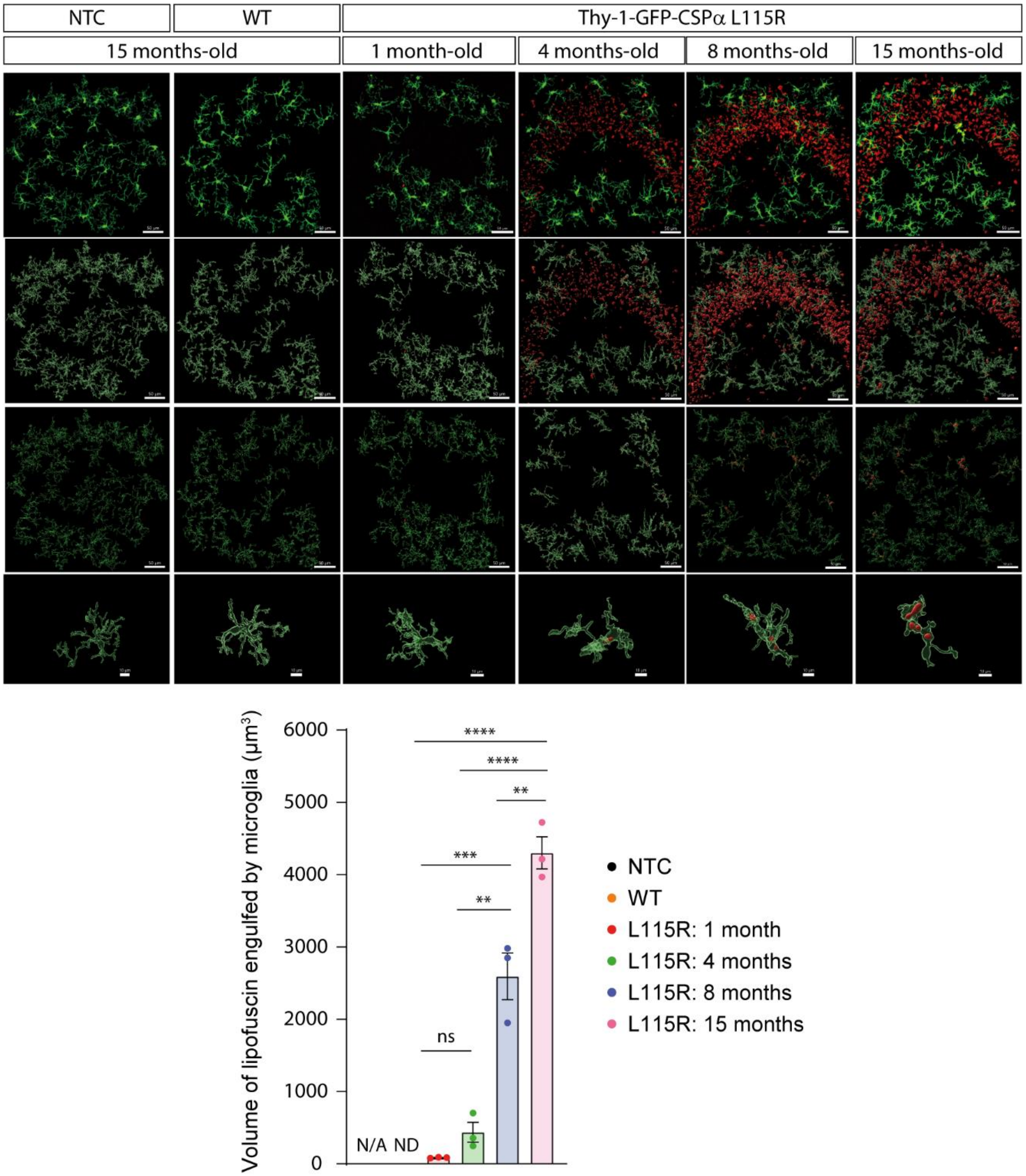

Figure S12. **Microglia engulf lipofuscin in Thy1-GFP CSPα-L115R transgenic mice. Upper panel.** First row*:* Representative confocal immunostaining images of microglia (Iba-1, green) and lipofuscin (red, autofluorescence, TRITC filter) at the CA3 region of control (NTC), GFP-CSPα-WT and Thy1-GFP CSPα L115R in 1, 4, 8 and 15 month-old mice. Second row: IMARIS 3D Reconstruction. Third row*:* IMARIS 3D Reconstruction of lipofuscin engulfed by microglia. Scale bar 50 μm and 10 μm for the fourth row. **Lower panel.** Quantification of volume of lipofuscin engulfed by microglia in control (NTC), GFP-CSPα-WT and GFP-CSPα-L115R at different time points (1, 4, 8 and 15 month-old). Data were presented as mean ± SEM, Two-way ANOVA with Tukey's post hoc test (***P* < 0.01; ****P* < 0.001; *****P < 0.0001), N* = 3 mice/group.

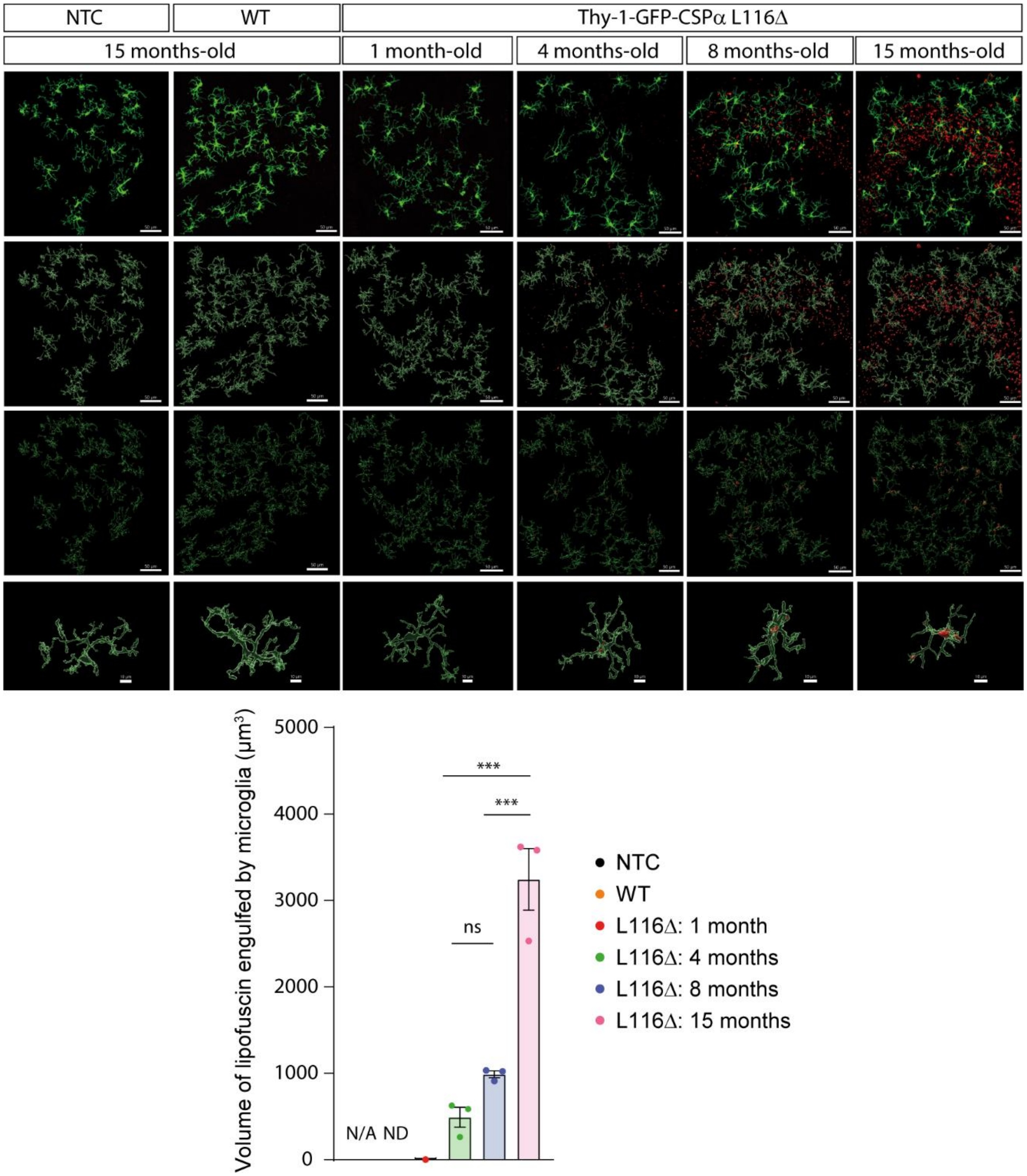

Figure S13. Microglia engulf lipofuscin in Thy1-GFP CSPα L116Δ transgenic mice. Upper panel. *First row:* Representative immunostaining images of microglia (Iba-1, green) and lipofuscin (red, autofluorescence at 561nm using the TRITC filter) at the CA3 region of control (NTC), GFP-CSPα-WT and Thy1-GFP CSPα L116Δ in 1, 4, 8 and 15 month-old mice. *Second row*: IMARIS 3D Reconstruction. *Third row:* IMARIS 3D Reconstruction of lipofuscin engulfed by microglia. Scale bar 50 μm and 10 μm for the fourth row. **Lower panel.** Quantification of volume of lipofuscin engulfed by microglia in control (NTC) and GFP-CSPα-WT, GFP-CSPα-L116*Δ* at 1, 4, 8 and 15 month-old. Data were presented as mean ± SEM, Two-way ANOVA with Tukey's post hoc test (****P* < 0.001), *N* = 3 mice/group.

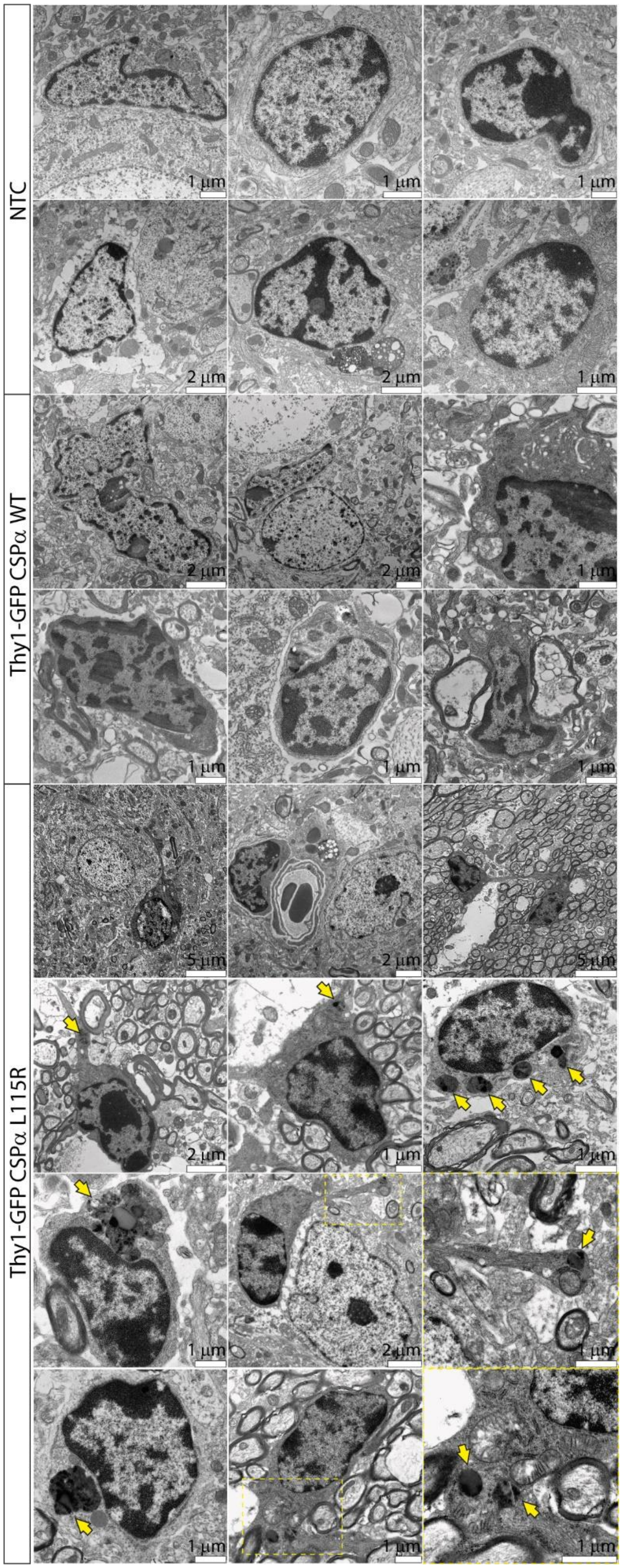

Figure S14. Electron microscopy images of microglia in aged Thy1-GFP-CSPα-L115R transgenic mice. Electron microscopy images of microglia cells in control (NTC), Thy-1-GFP-CSPα-WT, and Thy1-GFP-CSPα-L115R mice at 15 months of age, providing insights into their ultrastructural characteristics. Images are representative of microglia from three different mice per group.

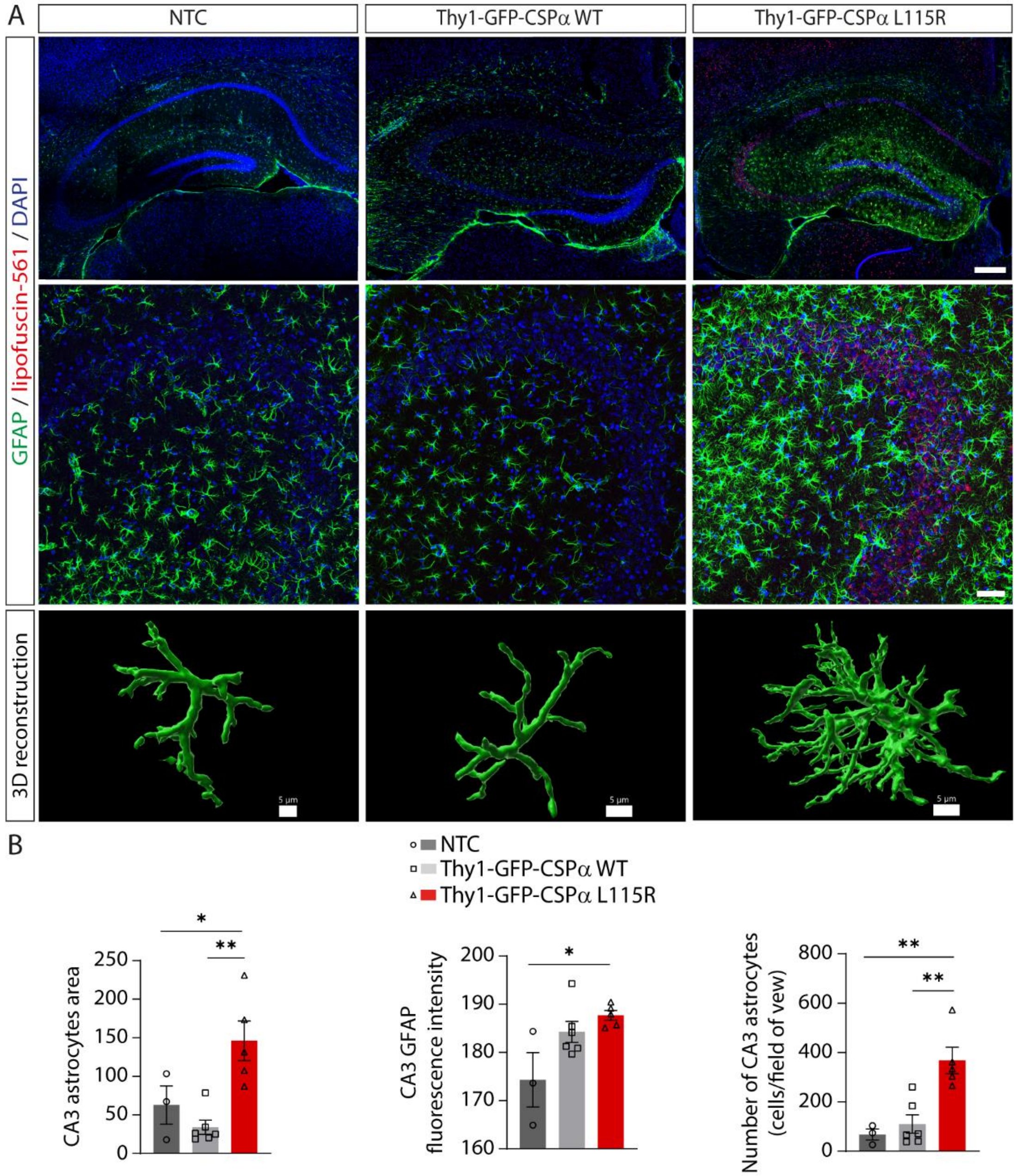

Figure S15. Enhanced astrocyte activation in Thy1-GFP-CSPα-L115R transgenic mice at 15 months of age. A. Representative images of immunostaining showing astrocytes using GFAP (green) and lipofuscin autofluorescence (red, detected at 561 nm using the TRITC filter) in the hippocampus. The first row displays the entire hippocampus, the second row focuses on the CA3 region, and the third row presents a representative 3D reconstruction of a single astrocyte cell, created using IMARIS software. The studied groups include non-transgenic control (NTC), Thy-1-GFP-CSPα-WT, and Thy1-GFP-CSPα-L115R mice at 15 months of age. **B**. Quantification of CA3 astrocytes was conducted to measure the area, fluorescence intensity, and the number of CA3 astrocytes per field of view. Scale bar: 50 μm, and 5μm for the third row. Data were presented as mean ± SEM, one-way ANOVA with Tukey's post hoc test (**P* < 0.05; ***P* < 0.01). N = 3-6 mice/group, and each data point represents the mean of 2 images/mouse.

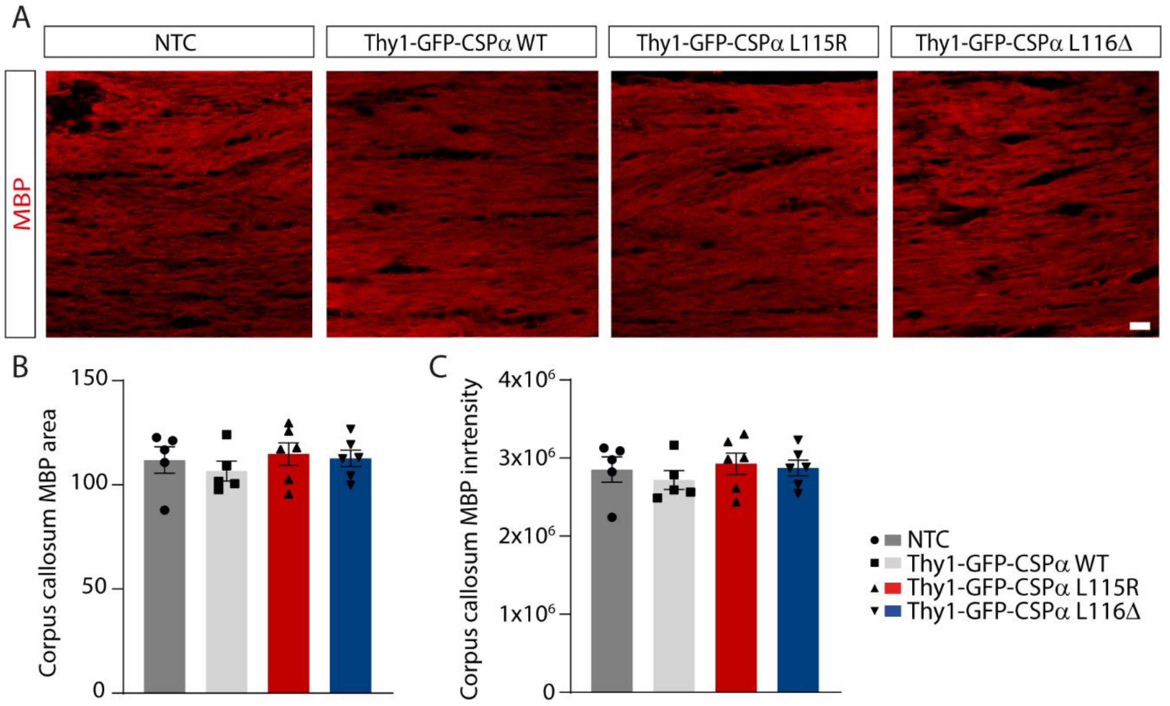

Figure S16. Normal myelination in Thy1-GFP-CSPα-L115R and Thy1-GFP-CSPα-L116Δ 15 months old transgenic mice. A. Immunofluorescence analyzing myelin basic protein (MBP) staining in the corpus callosum of 15-month-old mice in control (NTC), Thy1-GFP-CSPα-WT, Thy1-GFP-CSPα-L115R, and Thy1-GFP-CSPα-L116Δ mice. Quantitative analysis of MBP fluorescence intensity indicates no significant differences in MBP area (**B**) and intensity (**C**) among the different groups. Scale bar 10 μm. Data were presented as mean ± SEM, One-way ANOVA with Tukey's post hoc test. N = 3-4 mice/group, 1-2 images/mouse.

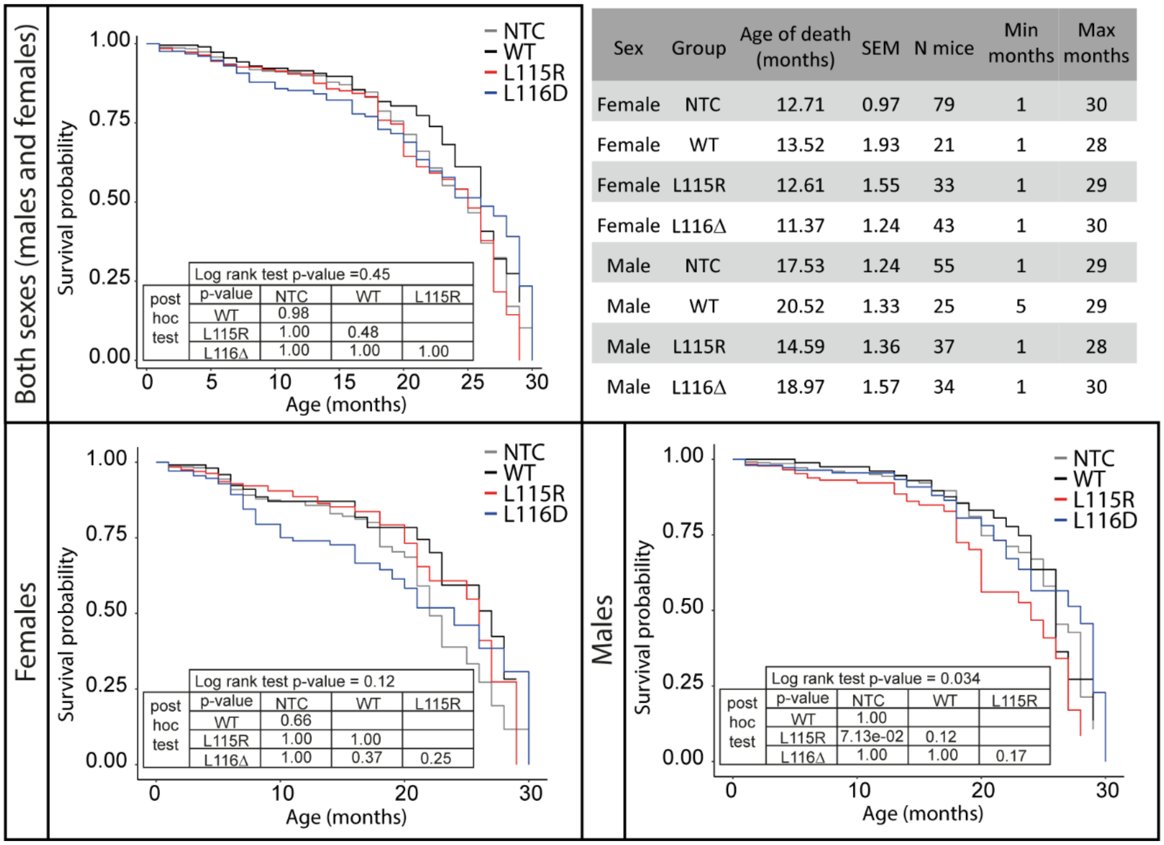

Sex	Group	Age of death (months)	SEM	N mice	Min months	Max months
Female	NTC	12.71	0.97	79	1	30
Female	WT	13.52	1.93	21	1	28
Female	L115R	12.61	1.55	33	1	29
Female	L116Δ	11.37	1.24	43	1	30
Male	NTC	17.53	1.24	55	1	29
Male	WT	20.52	1.33	25	5	29
Male	L115R	14.59	1.36	37	1	28
Male	L116Δ	18.97	1.57	34	1	30

Fig. S17. Survival probability analysis. Kaplan-Meier plot considering only the mice with annotated dates of natural death. No significant differences observed between different groups according to the log-rank test when mice of both sexes were analyzed. However, when female and male mice were independently analyzed, significant differences were detected in male mice in contrast to females mice. Thy1-GFP-CSPα-L115R mice had a lower survival probability compared to the other groups. The significant differences were observed using the log-rank test, followed by post-hoc pairwise analysis with Bonferroni correction. Differences were observed between males versus females in NTC and L116Δ (p-value for log-rank test NTC 0.00024, WT 0.42, L115R 0.52, L116Δ 0.0032).

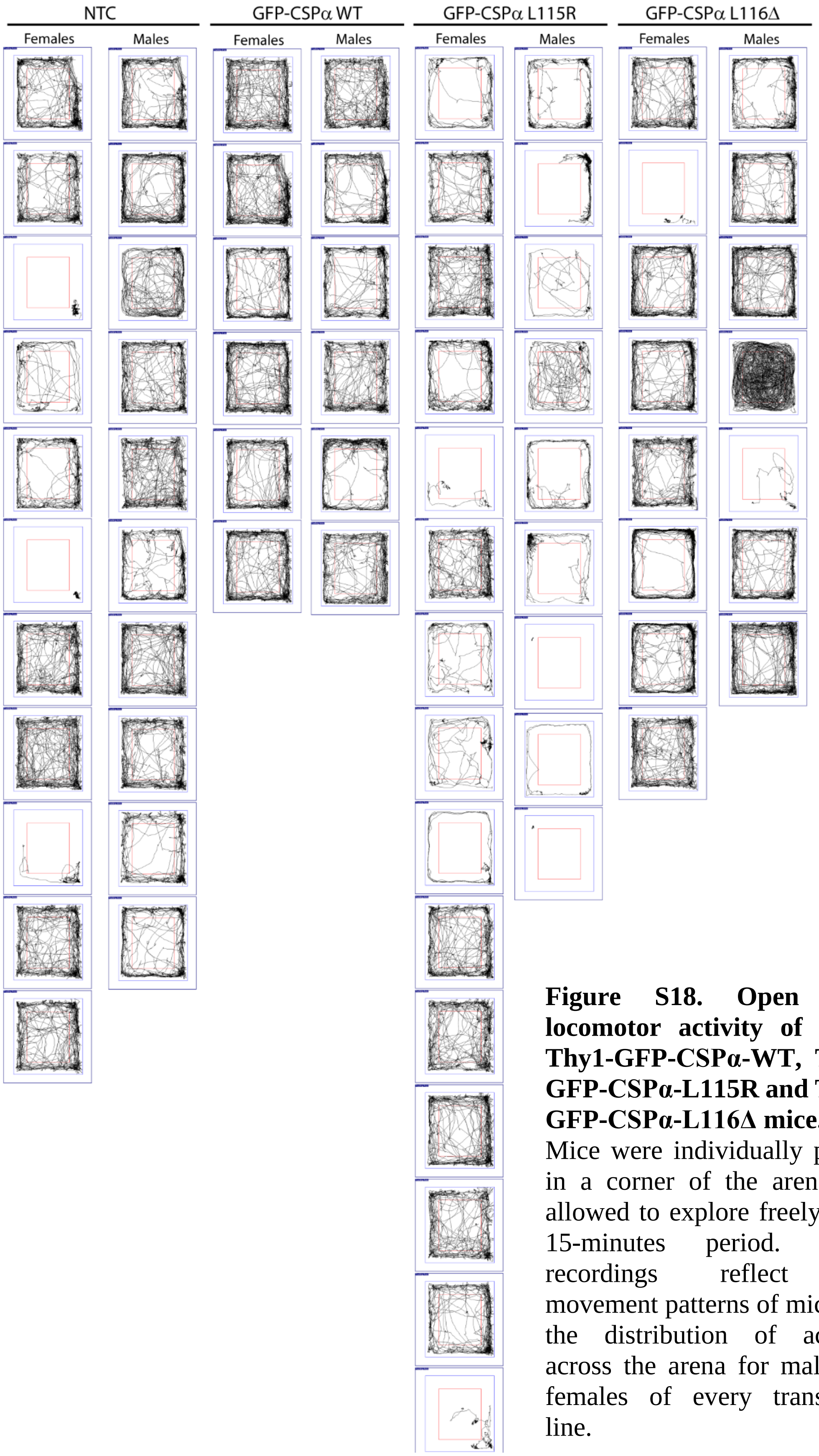

Figure S18. Open field locomotor activity of NTC, Thy1-GFP-CSPα-WT, Thy1-GFP-CSPα-L115R and Thy1-GFP-CSPα-L116Δ mice.
Mice were individually placed in a corner of the arena and allowed to explore freely for a 15-minutes period. Track recordings reflect the movement patterns of mice and the distribution of activity across the arena for male and females of every transgenic line.

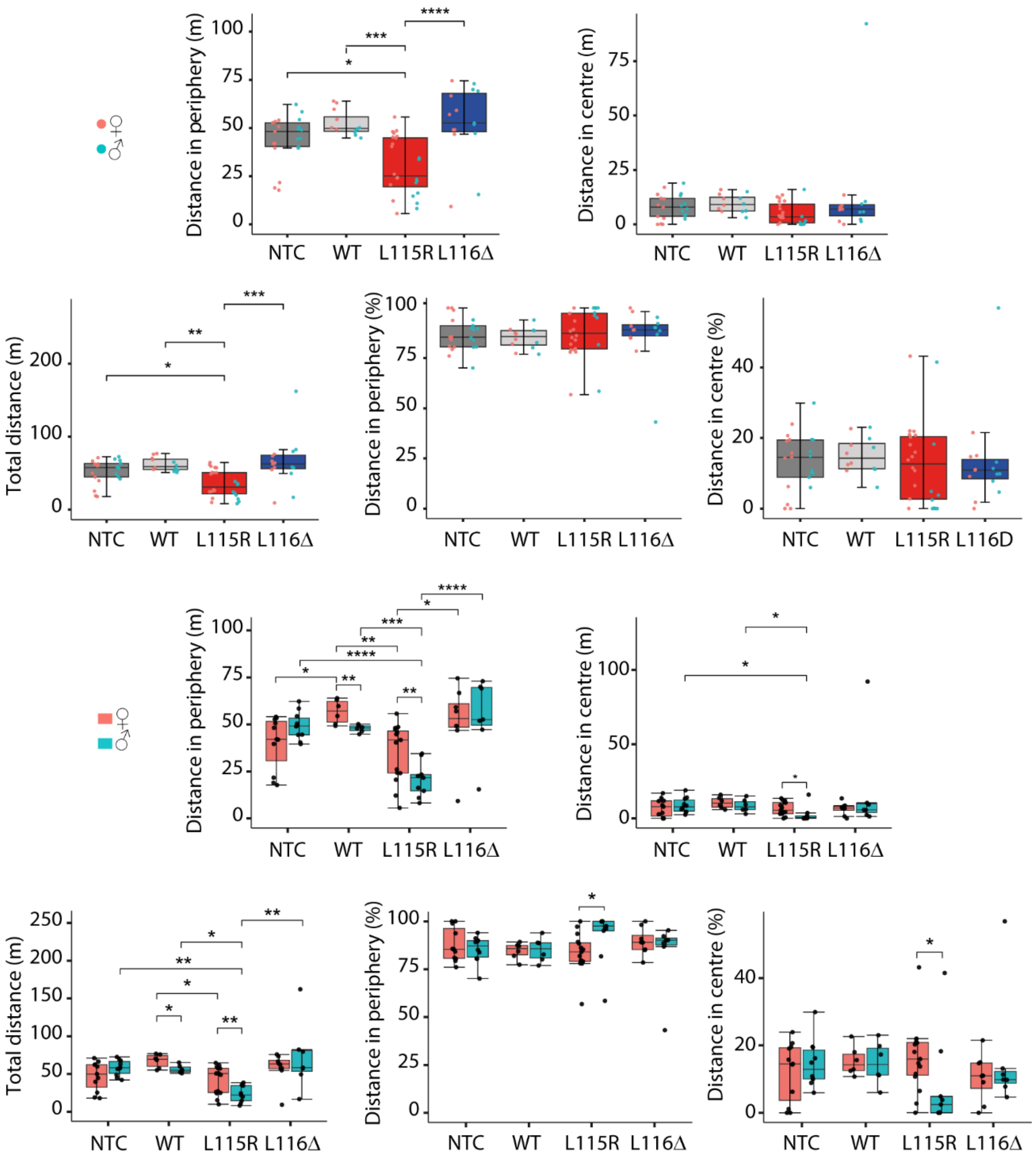

Figure S19. Distance traveled in the open field test by NTC, Thy1-GFP-CSPα-WT, Thy1-GFP-CSPα-L115R and Thy1-GFP-CSPα-L116Δ mouse lines. The figure illustrates the distance-related parameters measured in the open field test to evaluate locomotor activity in mice over a 15-minute period. **Upper rows:** combined results for both sexes. **Lower rows:** separated results for females and males. The most remarkable differences are in the distances travelled by the Thy1-GFP-CSPα-L115R that are shorter compared with other groups. This phenotype is stronger in male mice. Data are presented as mean ± SEM. Two-way ANOVA and Tukey's post-hoc test or Kruskal-Wallis and Dunn's post-hoc test (**P* < 0.05; ***P* < 0.01; ****P* < 0.001; *****P* < 0.0001). Upper panel displaying total distance is the same panel displayed in Fig 7E.

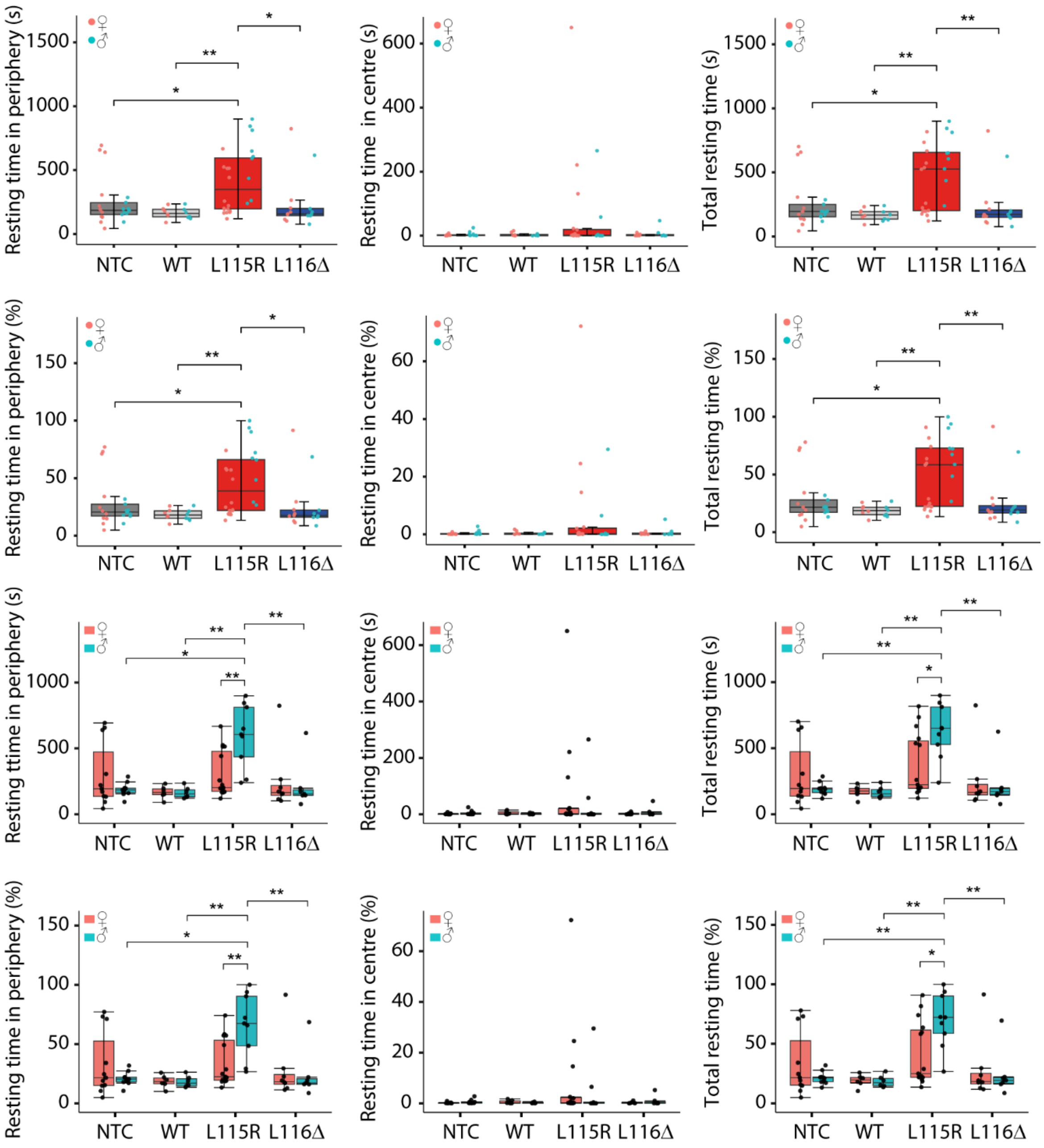

Figure S20. Resting time in the open field test for NTC, Thy1-GFP-CSPα-WT, Thy1-GFP-CSPα-L115R and Thy1-GFP-CSPα-L116Δ mouse lines. The figure illustrates the resting time-related parameters measured in the open field test to evaluate locomotor activity in mice over a 15-minute period. **Upper rows:** combined results for both sexes. **Lower rows:** separated results for females and males. The most remarkable differences are in the resting times of Thy1-GFP-CSPα-L115R that are longer compared with other groups. This phenotype is stronger in male mice. Data are presented as mean ± SEM. Data are presented as mean ± SEM. Two-way ANOVA and Tukey's post-hoc test or Kruskal-Wallis and Dunn's post-hoc test (**P* < 0.05; ***P* < 0.01; ****P* < 0.001; *****P* < 0.0001). Upper panel displaying total resting time is the same panel displayed in Fig 7E.

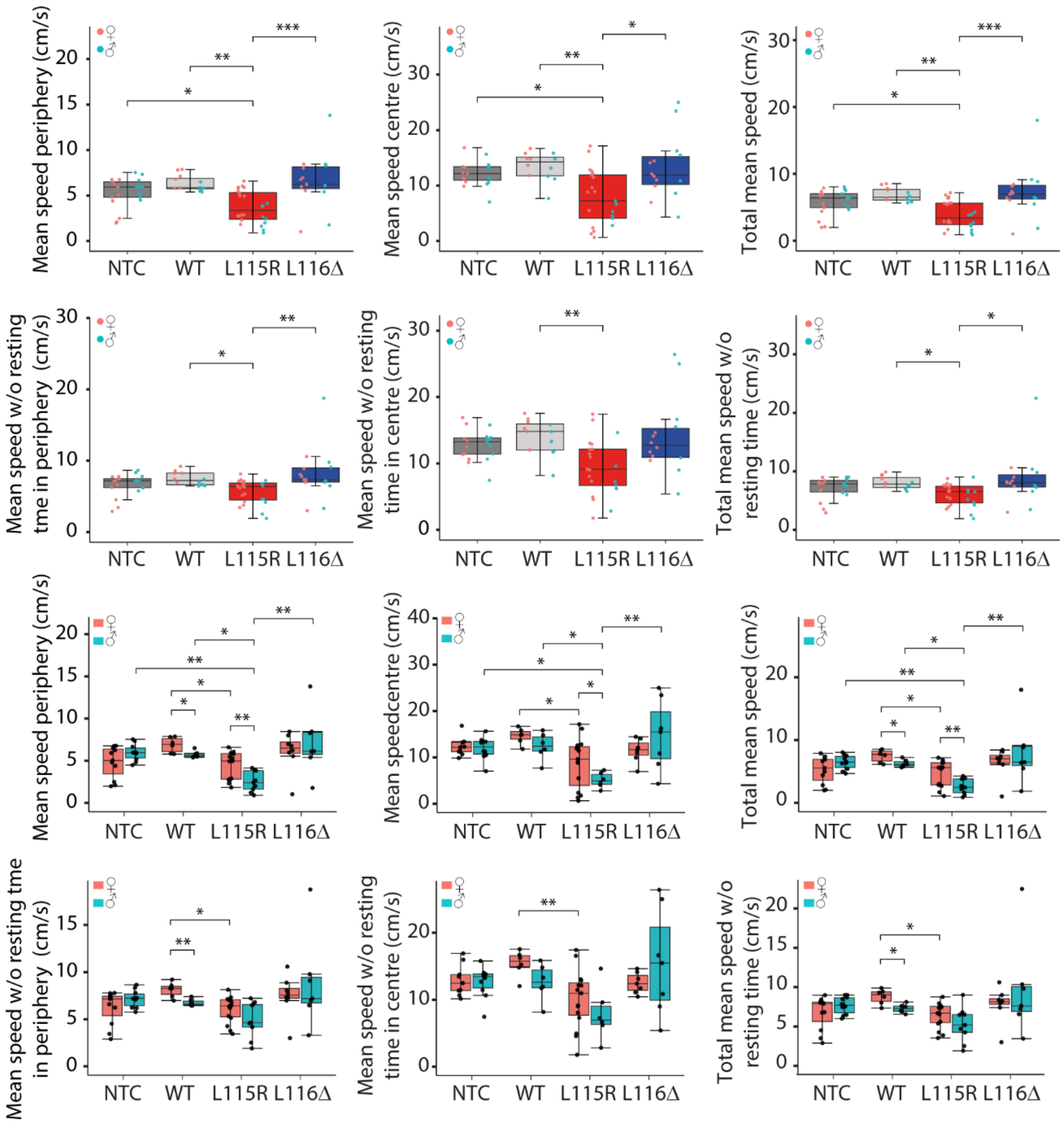

Figure S21. Speed in the open field test for NTC, Thy1-GFP-CSPα-WT, Thy1-GFP-CSPα-L115R and Thy1-GFP-CSPα-L116Δ mouse lines. The figure illustrates the speed-related parameters measured in the open field test to evaluate locomotor activity in mice over a 15-minute period. **Upper rows:** combined results for both sexes. **Lower rows:** separated results for females and males. There are differences in speed of Thy1-GFP-CSPα-L115R mice that move at lower speeds compared with other groups, especially in males. However, those differences become smaller or disappear when the resting time is not computed. Data are presented as mean ± SEM. Two-way ANOVA and Tukey's post-hoc test or Kruskal-Wallis and Dunn's post-hoc test (*$P < 0.05$; **$P < 0.01$; ***$P < 0.001$). Upper panels displaying total speed and total speed without resting time are the same panels displayed in Fig 7E.

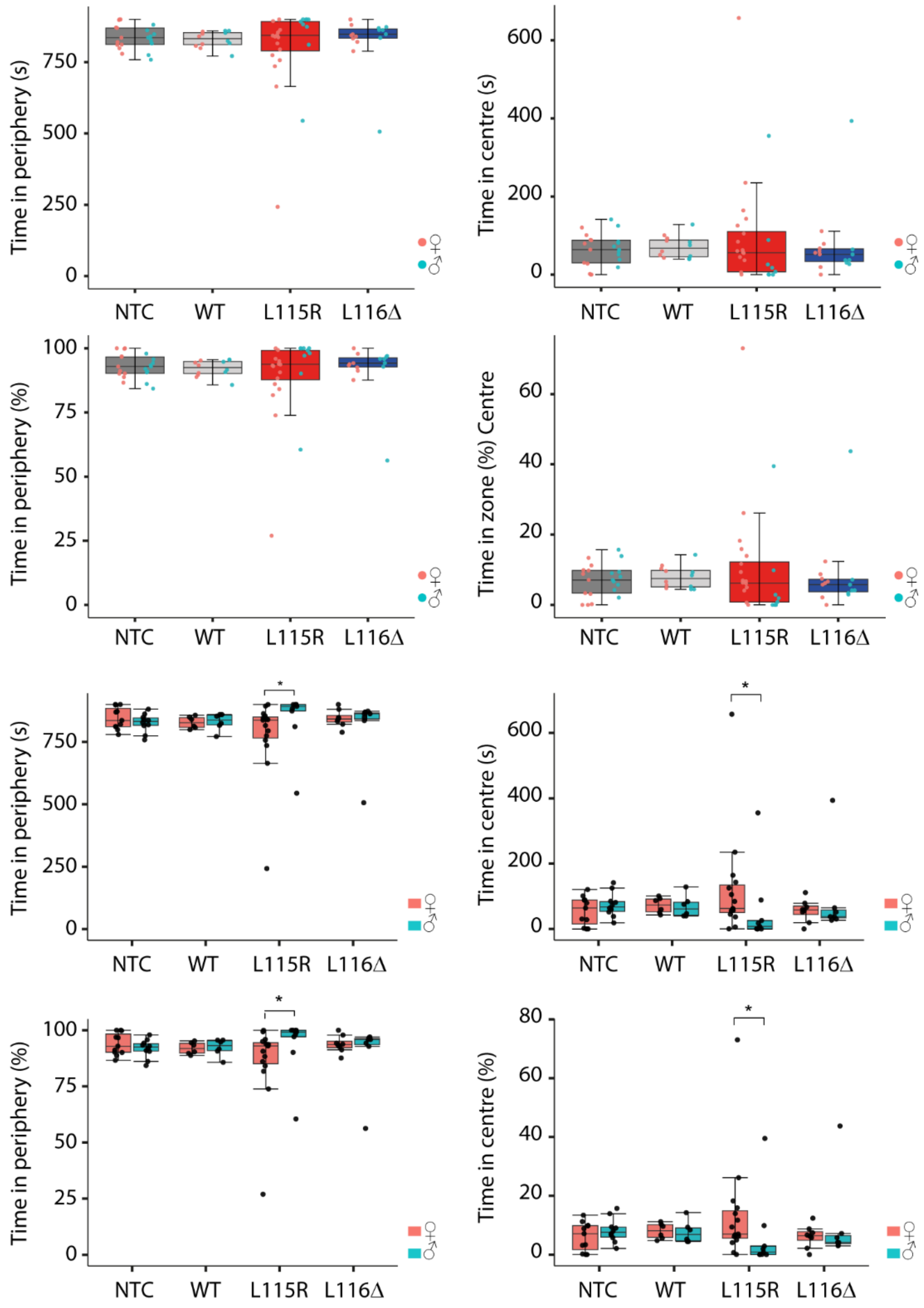

Figure S22. Time spent in center and periphery in the open field test for NTC, Thy1-GFP-CSPα-WT, Thy1-GFP-CSPα-L115R and Thy1-GFP-CSPα-L116Δ mouse lines. The figure illustrates the time spent in different zones of the arena over a 15-minute period. **Upper rows:** combined results for both sexes. **Lower rows:** separated results for females and males. There are no differences between groups, except that Thy1-GFP-CSPα-L115R male mice spent longer time in the periphery. Data are presented as mean ± SEM. Two-way ANOVA and Tukey's post-hoc test or Kruskal-Wallis and Dunn's post-hoc test (*$P < 0.05$).

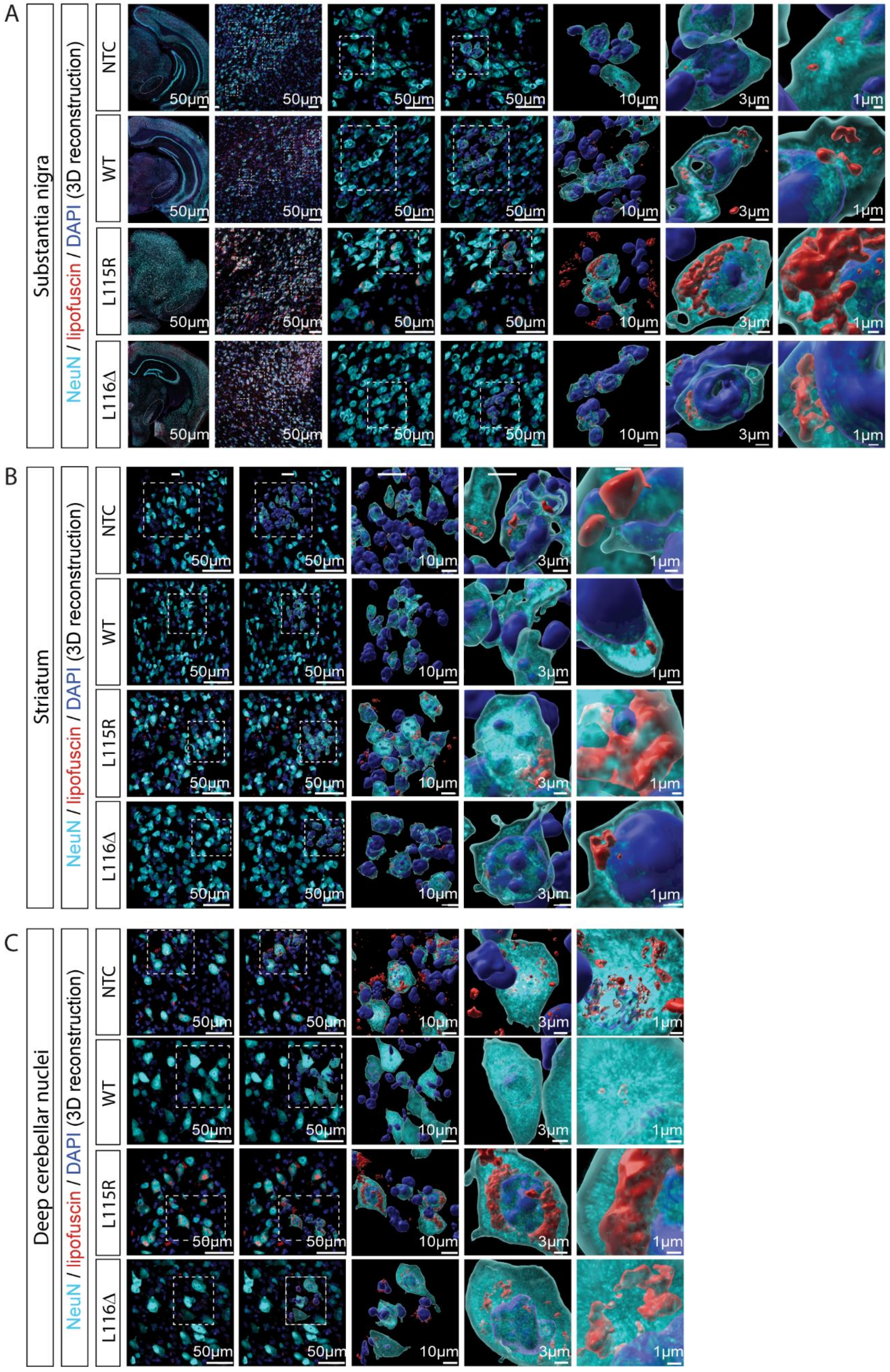

A
Substantia nigra
NeuN / lipofuscin / DAPI (3D reconstruction)
NTC
WT
L115R
L116Δ
50µm
10µm
3µm
1µm
B
Striatum
NeuN / lipofuscin / DAPI (3D reconstruction)
NTC
WT
L115R
L116Δ
C
Deep cerebellar nuclei
NeuN / lipofuscin / DAPI (3D reconstruction)
NTC
WT
L115R
L116Δ

Figure S23. Neuronal lipofuscinosis in brain areas of Thy1-GFP-CSPα-L115R and Thy1-GFP-CSPα-L116Δ transgenic mice involved in motor control. Imaris 3D reconstruction carried out from representative images of brain sections (1-year-old mice) labeled with antibodies against NeuN (cyan) to identify neurons. DAPI (blue) used to identify nuclei. Autofluorescence at 546nm to identify lipofuscin (red). Each row represents a genotype, from the top to the bottom: non-transgenic mice (NTC), Thy1-GFP-CSPα-WT (WT), Thy1-GFP CSPα-L115R (L115R) and Thy1-GFP-CSPα-L116Δ (L116Δ) mice. Each column shows different object magnification of Imaris 3D reconstruction. From the left to the right: overall view before tridimensional reconstruction, overall view after 3D reconstruction with a group of selected neurons (white square), magnification of neurons from white square; magnified selected neuron, and magnification of lipofuscin particles inside a neuron in substantia nigra (**A**), striatum (**B**) and deep cerebellar nuclei (**C**). N = 3 mice/group, n = 6 images/mouse.

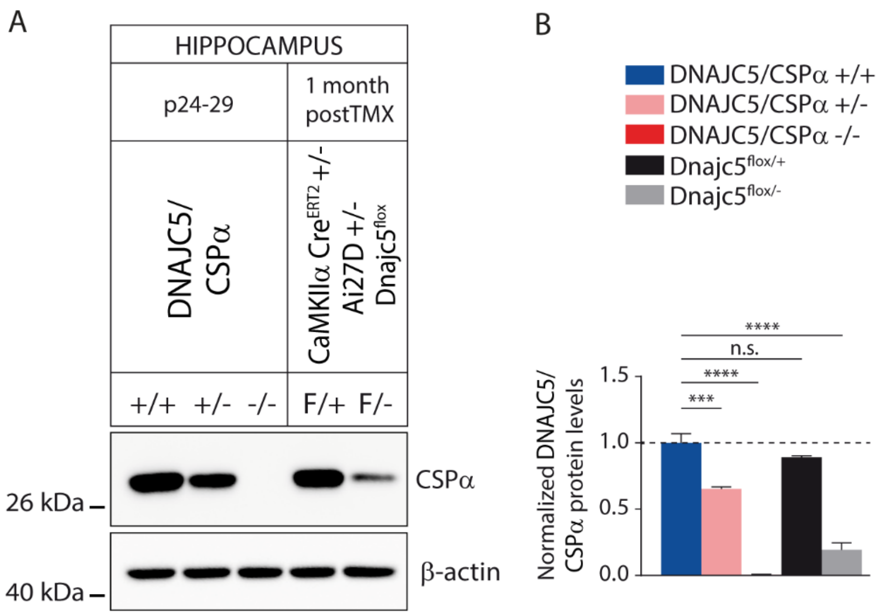

Figure S24. Reduced CSPα/DNAJC5 protein levels in hippocampus in conventional and in glutamatergic-specific conditional CSPα/DNAJC5 KO and heterozygous mice. A. Western blot of hippocampal extracts obtained from CSPα/DNAJC5 WT (+/+), heterozygous KO (+/-) and homozygous KO (-/-) mice at P24-P29 postnatal age and from 3-months-old CaMKIIα^{CreERT2}:Ai27D:Dnajc5$^{flox/+}$ and CaMKIIα^{CreERT2}:Ai27D:Dnajc5$^{flox/-}$ mice at 1 month after completing the tamoxifen-treatment. **B.** Quantitation of protein levels using beta-actin as a loading control reveals, as expected, reduction in the levels of CSPα/DNAJC5 in CSPα/DNAJC5 KO and heterozygous mice and in conditional CaMKIIα^{CreERT2}:Ai27D:Dnajc5$^{flox/-}$ mice.

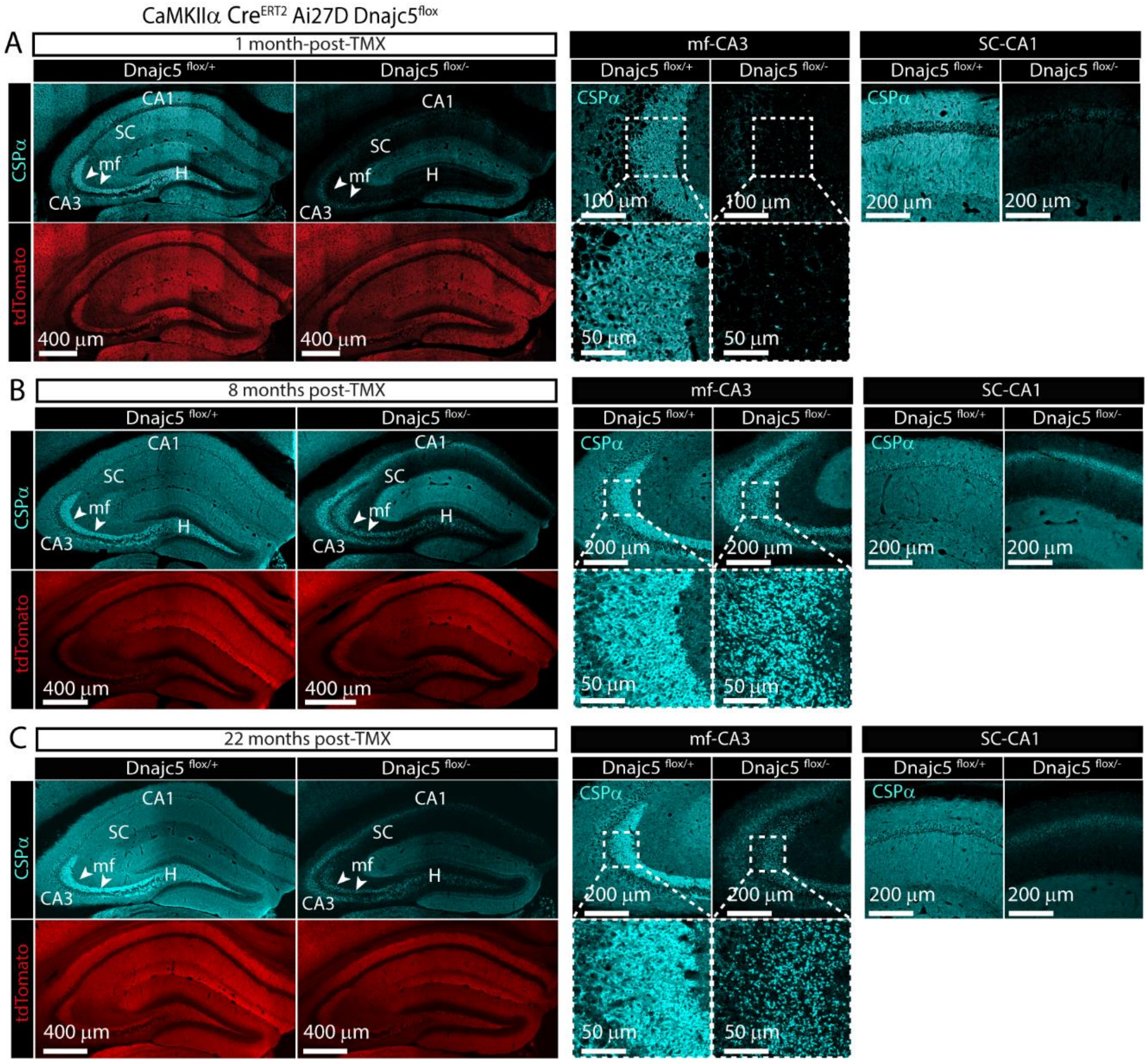

Figure S25. Selective genetic removal of CSPα/DNAJC5 from hippocampal glutamatergic neurons upon tamoxifen administration to CaMKIIα^{CreERT2}:Ai27D:Dnajc5$^{flox/-}$ mice. A. Three-months old CaMKIIα^{CreERT2}:Ai27D:Dnajc5$^{flox/+}$ and CaMKIIα^{CreERT2}:Ai27D:Dnajc5$^{flox/-}$ mice were fed with tamoxifen during 30 days and analyzed at 1-month post-tamoxifen (TMX) by immunolabeling with fluorescently labeled antibodies. Hippocampal sections from CaMKIIα^{CreERT2}:Ai27D:Dnajc5$^{flox/+}$ and CaMKIIα^{CreERT2}:Ai27D:Dnajc5$^{flox/-}$ conditional KO mice stained with antibodies against CSPα/DNAJC5 (cyan) and against tdTomato (red). Left panel, remarkable decrease of CSPα/DNAJC5 expression in pyramidal neurons at hippocampal CA1, CA3 regions and mossy fibers (mf). Right panel, magnification of mf-CA3 and SC (Schaffer collaterals)-CA1 synaptic layers reveals the strong decrease in CSPα/DNAJC5 levels. **B.** As in A, but mice were analyzed at 8-months post-tamoxifen (TMX). In contrast to the results obtained in mice analyzed at 1-month post-tamoxifen (TMX), the CSPα/DNAJC5 punctate staining at the stratum lucidum in the CA3 region likely corresponds to novel mossy fibers originated from granule cells that were born through adult

neurogenesis once the tamoxifen diet was discontinued. Cre-recombinase was never activated in these new granule cells and therefore CSPα/DNAJC5 expression proceeded normally. **C.** As in A, but mice were fed with tamoxifen at two months of age and analyzed at 22-months post-tamoxifen (TMX). As in B, CSPα/DNAJC5 expression is detected in a subpopulation of mossy fibers coming from newly born granule cells.

SUPPLEMENTARY METHODS

Table S2. Primary antibodies used for immunofluorescence and for western blot

Antigen	***Reference***	***Concent. Immunofluo-rescence***	***Concent. Western blot***
CSPα/DNAJC5	ADI-VAP-SV003-E, Enzo Life Sciences	1:500	1:10000
GFP	#GFP-1020, Aves Lab	1:500	1:1000
Synaptoporin	#102011, Synaptic System	1:500	X
ATP5G	#ab96655, Abcam	1:1000 Antigen Retrieval	1:5000
SNAP25	#836301 SMI81, BioLegend.	1:5000	1:10000
IBA1	Ab178847, Abcam	1:500	N/A
Neun	MAB377, Millipore	1:500	
Neun	#ABN78, Millipore	1:500	N/A
b-actin	#A2228, Sigma Aldrich	N/A	1:10000
Hsc70	#149011, Synaptic System	1:500	1:2000
tdTomato	#CPCA-mCherry EnCor Biotech	1:1000	N/A
pPERK	#sc-32577, Santa Cruz	1:800	N/A
CK1d	#PA5-32129, ThermoFisher	1:400	N/A
LIMP2	#NB400-129, Novus Biologicals	1:500	N/A
Alpha-Synuclein	Q698, TCS	1:500	N/A
Saposin D	#431003, Synaptic Systems	1:500	N/A
AT8	#MN1020, ThermoScientific	1:1000	N/A
AT100	#MN1060, ThermoScientific	1:1000	N/A
AT180	#MN1040, ThermoScientific	1:1000	N/A
GFAP	#60190, Proteintech	1:1000	N/A
CD68	#ab201340, Abcam	1:500	N/A

Table S3. Secondary antibodies used for immunofluorescence and for western blot.

Secondary antibody	*Reference*	*Concent. immunoflu-orescence*	*Concent. western blot*
Anti-Rabbit Alexa Fluor 488	#711-545-152 Jackson ImmunoResearch	1:500	N/A
Anti-Rabbit Cy3	#711-165-152 Jackson ImmunoResearch	1:500	N/A
Anti-Rabbit Alexa Fluor 647	#111-605-144 Jackson ImmunoResearch	1:500	N/A
Anti-Rabbit Biotin-SP	#.711-065-152 Jackson ImmunoResearch	1:1000	N/A
Anti-Mouse Alexa Fluor488	#715-545-151 Jackson ImmunoResearch	1:500	N/A
Anti-Mouse Cy3	#715-165-151 Jackson ImmunoResearch	1:500	N/A
Anti-Mouse Alexa Fluor 647	#115-605-146 Jackson ImmunoResearch	1:500	N/A
Anti-Mouse Alexa Fluor 790	#115-655-146 Jackson ImmunoResearch	1:500	N/A
Anti-Chicken Alexa Fluor488	#703-545-155 Jackson ImmunoResearch	1:500	N/A
Rhodamine Red anti Chicken	#703-295-155 Jackson ImmunoResearch	1:500	N/A
Alexa Fluor 647 anti-Chicken	#703-605-155 Jackson ImmunoResearch	1:500	N/A
Anti-Mouse HRP	#115-035-166 Jackson ImmunoResearch	N/A	1:10000
Anti-Rabbit HRP	#111-035-144 Jackson ImmunoResearch	N/A	1:10000
Anti-Chicken HRP	#103-035-155 Jackson ImmunoResearch	N/A	1:10000

Table S4. Primers used for cloning

Primer name	*Sequence (5'-3')*
ALR-2011-12	GTTTGTGGCCGCCTCACCTGCT
ALR-2011-13	AGCAGGTGAGGCGGCCACAAAC
ALR-2011-14	GTTTGTGGCCTCACCTGCT
ALR-2011-15	AGCAGGTGAGGCCACAAAC

Table S5. Primers used for genotyping

Gene	Primer name	Sequence 5' - 3'	Primer type	Product size
Dnajc5 (conventional KO)	25938	CAAGAATGCAACCTCAGATGAC	Wild-type Forward	KO = 150 bp<br>HET = 150/336 bp<br>WT = 336 bp
	25939	CTTTTAAGTGTGTTTACTTTTTGGTG	Common	
	Oimr8574	GAGCGCGCGCGGCGGAGTTGTTGAC	Mutant Forward	
Dnajc5$^{flox/flox}$ (conditional KO)	FMS-2014-14	TATCGGTAAGCAGCCGTGTTAACC	Transgene Forward	Homozygous = 900 bp<br>Hemizygous = 900/678 bp<br>WT = 678 bp
	FMS-2014-15	TATAGCATTCACTCCTGCCAACCC	Transgene Reverse	
GFP-CSPα transgene	ALR-2015-01	CTGACCTGTAGCTTTCCCCA	Transgene Forward	Transgene = 192bp
	ALR-2015-02	GCTGAACTTGTGGCCGTTTA	Transgene Reverse	
ChR2-tdTomato	oIMR9020	AAGGGAGCTGCAGTGGAGTA	Wild type Forward	Mutant = 315bp<br>WT = 297bp
	oIMR9021	CCGAAAATCTGTGGGAAGTC	Wild type Reverse	
	oIMR9103	GGCATTAAAGCAGCGTATCC	Mutant Forward	
	oIMR9105	CTGTTCCTGTACGGCATGG	Mutant Reverse	
CaMKIIα CreERT2	ERT2-1	GGTTCTCCGTTTGCACTCAGGA	Wild type allele	Mutant = 375bp<br>HET = 375/290bp<br>WT = 290bp
	ERT2-2	CTGCATGCACGGGACAGCTCT	Wild type allele	
	ERT2-3	GCTTGCAGGTACAGGAGGTAGT	Cre-ERT2 insert	
WT	SLB_24_16	CAAGACTAAGGGGCCTGTCTTC	WT endo	NTC = 315bp<br>Het = 315/656bp<br>Homo= 656bp
	SLB_23_17	CTCTGCCTTTTGCCCTCTG	Trangene cassette	
	SLB_24_21	CACCAAACGCAGACACTATTGC	WT_endo_left_arm	
L115R	SLB_24_10	CAATGTGCCTGTGTTTAGTTTC	WT endo	Left arm. NTC = 729 bp Het = 729bp/393 bp Homo = 393bp
	SLB_24_22	GGATAATATATCCAGCAAAACTCTC	L115R_endo_left_arm	
	SLB_23_31	GGACTCCTGCCCATATGTCCTAAG	Transgene cassette	
	SLB_23_30	CTGAACTCAGGCTGTCAGGCTTGC	Transgene cassette	Right arm. NTC = 642 bp<br>Het = 642/219 bp<br>Homo = 219 bp
	SLB_24_13	GTGGTCCATCCAATAGCTGAC	WT endo	
	SLB_24_23	GGTAACTGGAAGAAGCTGCAAC	L115R_endo_right_arm	

L116Δ	SLB_24_14	CAGGGCATCAGGACTTTGTTC	WT endo	Left arm. NTC = 180 bp Het = 180bp/608 bp Homo = 608 bp
	SLB_23_31	GGACTCCTGCCCATATGTCCTAAG	Transgene cassette	
	SLB_24_24	CTGTAAAGATAAAAAGGTATGCAAGG	L116_endo_left_arm	
	SLB_24_01	GAGAATATTAGAGTATTATTCCTCCCC	L116_endo_right_arm	Right arm. NTC = 933 bp<br>Het = 933/822 bp<br>Homo = 822 bp
	SLB_23_31	GGACTCCTGCCCATATGTCCTAAG	Transgene cassette	
	SLB_24_03	CTGTAGAAACTTGATGCCCCAG	WT endo	

Table S1 (Data, Excel file)

Quantitative data related to Figures 1, 2, 3, 4, 8, 9, 10, S1, S3, S10, S11, S12, S13, S24 and data related to the survival of CaMKIIαCreERT2Ai27D:Dnajc5flox/- conditional KO mice.

Movie S1-GFP-CSPα-WT

Video showing Thy1-GFP-CSPα-WT mice (mice numbers 189,188,193, 181) placed individually on the rotarod, with a rotation speed gradually increased in a stepwise manner (1 rpm every 5 seconds from 5 to 40 rpm for 3 minutes) until the mouse fell off the rod or clung to it. Rotarod experiments were performed in mice at 10-15 months of age.

Movie S2-GFP-CSPα-L115R

Video showing Thy1-GFP-CSPα-L115R mice (mice numbers 144,153,162, 161) placed individually on the rotarod, with a rotation speed gradually increased in a stepwise manner (1 rpm every 5 seconds from 5 to 40rpm for 3 minutes) until the mouse fell off the rod or clung to it. Rotarod experiments were performed in mice at 10-15 months of age.
